# Proceedings of the 24th Annual Meeting of the Portuguese Society of Human Genetics (SPGH – *Sociedade Portuguesa de Genética Humana*)

**DOI:** 10.1097/MD.0000000000023585

**Published:** 2021-01-29

**Lisbon, 20th November 2020**

**Figure:**
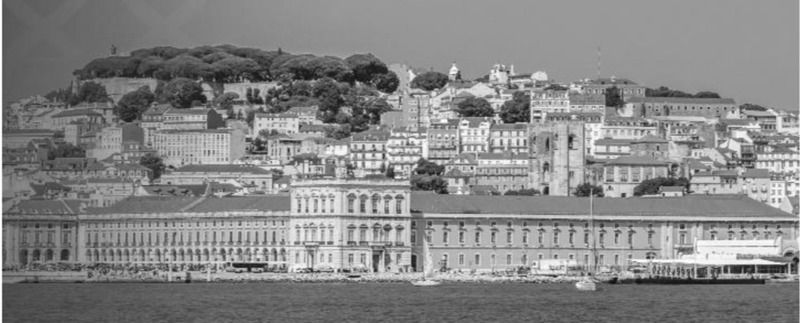


**LETTER FROM THE SPGH PRESIDENT**

In a year of surprises and extraordinary challenges, regardless of where we live or work, we have been facing an extremely small entity and fighting against it, although we do not have the tools and the natural defences sufficiently developed yet.

Bearing in mind that the “dream is a constant in life” and that SPGH has a past full of widely recognized scientific events, the SPGH board of Directors together with its Scientific Commission, facing the pandemic that has deeply disturbed us, reacted with determination and persistence keeping in 2020 the main activity of the Portuguese Society of Human Genetics, its annual scientific meeting.

Aiming at providing SPGH members and all Portuguese researchers working in Human Genetics the opportunity to present their research and at offering them relevant talks in updated scientific topics, internationally recognized researchers were invited to the 24th Annual Meeting of SPGH. We deeply thank Prof. Kym Boycott and Prof. Jean-Laurent Casanova, for having accepted our invitation to give a lecture on the topics in which they are experts, “Rare Genetic Diseases” and “Inborn errors of immunity to Sars-CoV-2”, respectively.

SPGH members and other researchers, from different Portuguese institutions, contributed with high quality abstracts, encompassing fundamental research, diagnosis, and genetic counselling.

SPGH is proud to provide to all those who submitted their work to the 24th Annual Meeting of our Society (www.spgh.net), the publication of the abstracts in the journal Medicine, thus contributing to a wide dissemination of the research in Human Genetics performed in Portugal.

***João Gonçalves***

SPGH President

**ORAL PRESENTATIONS**

**BASIC RESEARCH**

**OP1 –** GENE EDITED IPSC-DERIVED CARDIOMYOCYTES REVEAL RNA SPLICING DEFECTS IN HYPERTROPHIC CARDIOMYOPATHY

Marta Furtado^1^; Sandra Martins^1^; Teresa Carvalho^1^; Marta Ribeiro^1^; João Proença^2^; Vasco Barreto^3^; Maria Carmo-Fonseca^1^

^1^Instituto de Medicina Molecular João Lobo Antunes, Faculdade de Medicina, Universidade de Lisboa; ^2^Instituto de Medicina Molecular João Lobo Antunes, Faculdade de Medicina, Universidade de Lisboa. Centro de Estudos de Doenças Crónicas, Faculdade de Ciências Médicas, Universidade NOVA de Lisboa; ^3^Centro de Estudos de Doenças Crónicas, Faculdade de Ciências Médicas, Universidade NOVA de Lisboa.

**Introduction:** Familial hypertrophic cardiomyopathy (HCM) is the most common inherited heart disease and the leading cause of sudden cardiac death, namely in young athletes. The disease is caused by heterozygous mutations in genes that encode cardiac sarcomere-associated proteins, and *MYPBC3* is one of the most frequently affected genes. Most *MYBPC3* mutations are classified as nonsense, frameshift or splice site mutations, but several missense mutations have also been reported. Recent analysis of MYBPC3 mRNA in patient samples showed that exonic variants classified as missense in fact caused exon skipping [1]. The aim of this study was to determine whether the *MYBPC3* c.1090G > A pathogenic variant currently annotated as missense interferes with normal splicing in cardiomyocytes differentiated *in vitro* from human induced pluripotent stem cells (iPSC-CMs).

**Methodology:** CRISPR/Cas9 was used to generate isogenic wild type and mutant iPSC lines. The iPSC-CMs were characterized by immunofluorescence, RT-PCR, and Western Blot.

**Results:** Compared to wild type iPSC-CMs at day 30 of differentiation, the mutant iPSC-CMs showed increased cellular size, multinucleation, and disorganized sarcomeres, thus recapitulating HCM-specific features. Reduced levels of *MYBPC3* mRNA and myosin binding protein-C were detected in mutant cells. Sequencing analysis revealed skipping of exon 12 in mutant transcripts. Exon 12 skipping causes frameshift and introduces a premature termination codon. Treatment with cycloheximide confirmed that mutant transcripts are targeted for non-sense mediated decay (NMD).

**Discussion:** We demonstrate that the *MYBPC3* c.1090G > A pathogenic variant is indeed a mutation that induces exon skipping, degradation of mutant transcripts by NMD, and reduced protein levels, thus causing disease by a mechanism of haploinsufficiency. In conclusion, this study underscores the need of revising the classification of HCM- associated missense mutations in the *MYBPC3* gene.

**References:** [1] Ribeiro M, Furtado M, Martins S, Carvalho T, Carmo- Fonseca M (2020) RNA splicing defects in hypertrophic cardiomyopathy: implications for diagnosis and therapy. DOI: 10.3390/ijms21041329.

**OP2 –** PERMANENT INACTIVATION OF THE *ATXN3* GENE: A POSSIBLE THERAPEUTIC APPROACH FOR MACHADO-JOSEPH DISEASE

Sara M. Lopes^1^; Miguel M. Lopes^2^; Rui J. Nobre^1^; Clévio Nóbrega^3^; Carlos A. Matos^3^; Ana Vasconcelos-Ferreira^4^; Neville E. Sanjana^5^; Patrick D. Hsu^5^; F Ann Ran^5^; Lukasz Swiech^5^; Le Cong^5^; Feng Zhang^5^; Luís Pereira de Almeida^4^

^1^Center for Neuroscience and Cell Biology (CNC), University of Coimbra, Portugal; Institute for Interdisciplinary Research (IIIUC), University of Coimbra, Portugal; ^2^Center for Neuroscience and Cell Biology (CNC), University of Coimbra, Portugal; ^3^Center for Neuroscience and Cell Biology (CNC), University of Coimbra, Portugal; Department of Biomedical Sciences and Medicine, University of Algarve, Faro, Portugal; Center for Biomedical Research, CBMR, University of Algarve, Faro, Portugal; ^4^Center for Neuroscience and Cell Biology (CNC), University of Coimbra, Portugal; Faculty of Pharmacy, University of Coimbra, Portugal; ^5^Broad Institute of Massachusetts Institute of Technology and Harvard, Cambridge, Massachusetts, USA.

**Introduction:** Machado-Joseph disease (MJD) is an autosomal dominantly inherited neurodegenerative disorder, caused by an over-repetition of the polyglutamine- codifying region in the *ATXN3* gene. Therapeutic strategies based on gene silencing represent promising approaches for MJD, although gene suppression is only partial and transient. Recently, genome editing technologies, such as TALENs and CRISPR-Cas9 systems, have been successfully used to interfere with disease-related genes, assuring a permanent gene inactivation at the most upstream level.

**Material & Methods:** Here, a panel of customized sequences were designed and constructed aiming at permanently suppressing the human *ATXN3* gene expression. Functional characterization was performed in HEK293T cells, through the surveyor mutation detection assay. One sequence of each system was selected and intracranially delivered to an *in vivo* lentiviral mouse model of MJD. Neuropathological markers were assessed 4 weeks after surgery.

**Results:** Surveyor mutation detection assay revealed the editing capability of our customized nucleases both in HEK293T cells and in striatal samples of the mouse model. We observed a reduction in the levels of wild-type ataxin-3 in human cells and a drastic reduction of the mutant protein in the mouse model, in a dose-dependent manner. Immunohistochemical analysis of mouse brain sections revealed the same tendency for the reduction of aggregates in the striatum.

**Conclusions:** This work provides the first *in vivo* evidence of the efficacy of gene editing-based approaches to permanently inactivate the *ATXN3* gene, supporting their potential as putative therapeutic avenues in the context of MJD.

This work was funded by the ERDF through the Regional Operational Program Center 2020 and National Funds through FCT - CENTRO-01- 0145-FEDER-000008, CENTRO-01-0145-FEDER-022095, POCI-01-0145-FEDER-016719, POCI-01-0145-FEDER-029716 and PTDC/BBB-NAN/0932/2014, by SynSpread, ESMI and ModelPolyQ under the EU Joint Program - Neurodegenerative Disease Research; National Ataxia Foundation, the American Portuguese Biomedical Research Fund and the Richard Chin and Lily Lock Machado-Joseph Disease Research Fund.

**OP3 –** THE TUMOR SUPPRESSOR *P53* ACQUIRES ONCOGENIC FUNCTIONS DUE TO A TRANSLATIONAL SWITCH DURING INTEGRATED STRESS RESPONSE

Rafaela Lacerda^1^; Inês Fonseca costa^1^; Maria López-Iniesta^2^; Luísa Romão^3^; Marco Candeias^2^

^1^MaRCU — Molecular and RNA Cancer Unit; Departamento de Genética Humana, Instituto Nacional de Saúde Dr. Ricardo Jorge, Lisboa, Portugal; BioISI — BioSystems & Integrative Sciences, Faculdade de Ciências, Universidade de Lisboa, Lisboa, Portugal; ^2^MaRCU — Molecular and RNA Cancer Unit; Graduate School of Medicine, Kyoto University, Kyoto, Japan; ^3^Departamento de Genética Humana, Instituto Nacional de Saúde Dr. Ricardo Jorge, Lisboa, Portugal; BioISI — BioSystems & Integrative Sciences, Faculdade de Ciências, Universidade de Lisboa, Lisboa, Portugal

To cope with the stress stimuli to which they are often exposed, eukaryotic cells have developed adaptive pathways that restore cellular homeostasis. Under stress conditions there is an overall decrease of protein synthesis, and a concomitant induction of alternative mechanisms of mRNA translation initiation.

The tumour suppressor protein *p53* has been considered the guardian of the genome and a master regulator of many cellular functions. However, apart from the full-length *p53* (FLp53), several *p53* isoforms have been described so far. Some functions of shorter *p53* isoforms have already been elucidated and they are different from and complement FLp53 activity, the most mutated gene in cancer.

Here we show that the integrated stress response (ISR) leads to the specific induction of Δ160*p53* isoform. Using bicistronic constructs we confirmed the presence of an Internal Ribosome Entry Site (IRES) in *p53* mRNA that controls Δ160p53 isoform translation. Subjecting cells to Human Genetics, Univ Leuven, Leuven, Belgium; ^6^Laboratory of Human Genetics, GIGA-Institute, University of Liège, Liège, Belgium; Center of Genetics, University Hospital (CHU), Liège, Belgium; European Reference Network on Genetic Tumour Risk Syndromes (ERN GENTURIS); ^7^Clinical Genetics department, University Hospital of Ghent, Ghent, Belgium; European Reference Network on Genetic Tumour Risk Syndromes (ERN GENTURIS); ^8^Service de Génétique Oncologique, Institut Curie, Paris, France; ^9^Service de Génétique Oncologique, Institut Curie, Paris, France; 5European Reference Network on Genetic Tumour Risk Syndromes (ERN ^10^ endoplasmic reticulum stress showed that eIF2α GENTURIS); Département de Génétique, GH Pitié- Salpêtrière, Paris, phosphorylation is a key event leading to cap-independent expression of Δ160p53 during ISR.

Additionally, cancer-specific mutations in p53 also enhanced cap-independent translation of Δ160p53 via Δ160p53IRES. An antisense morpholino oligo targeting Δ160IRES efficiently reduced Δ160p53 protein levels and significantly impaired oncogenic functions of Δ160p53.

Our data support a model in which an IRES structure in the France; ^11^Institute of Human Genetics, University Hospital Bonn, Bonn, Germany; European Reference Network on Genetic Tumour Risk Syndromes (ERN GENTURIS); ^12^Department of Internal Medicine I, University Hospital Bonn, Bonn, Germany; National Center for Hereditary Tumor Syndromes, University Hospital Bonn, Bonn, Germany; ^13^TU Dresden, Medical Faculty CGC, Institute for Clinical Genetics, Dresden, Germany; ^14^TU Dresden, Medical Faculty CGC, Institute for Clinical Genetics, Dresden, Germany; European Reference Network on Genetic Tumour Risk Syndromes (ERN GENTURIS); ^15^Medizinische Klinik und Poliklinik IV, Klinikum der Universität München, Munich, Germany; Medizinisch Genetisches Zentrum, Munich, Germany; European Reference Network on Genetic Tumour Risk ^16^coding region of p53 is activated under stress conditions, Syndromes (ERN GENTURIS); Biosciences Laboratory, Istituto Scientifico leading to the expression of the oncogenic shorter Δ160p53 isoform, whose structure is affected by cancer-specific Romagnolo per lo Studio e la Cura dei Tumori (IRST) IRCCS, Meldola, Italy; ^17^Department of Biology and Biotechnology “Lazzaro Spallanzani; ^18^Institute of Genomic Medicine, Catholic University of the mutations in the p53 gene. A better understanding of Sacred Heart, Rome, Italy; ^19^Genomed Diagnósticos de Medicina Molecular, Δ160p53IRES structure and function may be advantageous for a more efficient therapeutic targeting of p53.

**CLINICAL RESEARCH**

**OP4 –** GENOTYPE-PHENOTYPE ASSOCIATIONS PROVIDE A RATIONAL TO IDENTIFY POTENTIALLY ACTIONABLE VUS

José Peláez^1^; Rita Monteiro, Silvana Lobo, Liliana Sousa & Hugo Pinheiro^1^; Sergio Castedo^2^; Luzia Garrido^3^; Manuel Teixeira^4^; Genevieve Michils^5^; Vicent Bours^6^; Robin de Putter^7^; Lisa Golmard & Maud Blanluet^8^; Chrystelle Colas^9^; Patrick Benusiglio, Camille Desseignés & Coulet Florence^10^; Stefan Aretz & Isabel Spier^11^; Robert Hüneburg^12^; Laura Gieldon^13^; Evelyn Schrock^14^; Elke Holinski-Feder & Verena Steinke^15^; Daniele Calistri & Gianluca Tedaldi^16^; Guglielmina Ranzani^17^; Maurizio Genuardi^18^; Catarina Silveira & Inês Silva^19^; Mateja Krajc & Ana Blatnik^20^; Srdjan Novacovik^21^; Ana Patiño-García^22^; José L. Soto^23^; Conxi Lázaro & Gabriel Capellá^24^; Joan Brunet-Vidal^25^; Judith Balmaña^26^; Elena Domínguez-Garrido^27^; Marjolin Ligtenberg^28^; Eleanor Fewings^29^; Rebecca Fitzgerald^30^; Emma Woodward^31^; Gareth Evans^32^; Helen Hanson^33^; Kristina Lagerstedt-Robinson^34^; Svetlana Bajalica- Lagercrantz^35^; Conceição Egas^36^; M. Isabel Tejada^37^; Karim Dahan & Damien Feret^38^; Nicoline Hoogerbrugge^28^; Marc Tischkowitz^39^; Carla Oliveira^40^

^1^IPATIMUP, i3S, Porto, Portugal; ^2^IPATIMUP, i3S, Porto, Portugal; Centro Hospitalar Universitário São João (CHUSJ), Porto, Portugal & Faculty of Medicine of the University of Porto (FMUP), Porto, Portugal; ^3^Centro Hospitalar Universitário São João (CHUSJ), Porto, Portugal; ^4^IPO, Instituto Português de Oncologia do Porto, Porto, Portugal; European Reference Network on Genetic Tumour Risk Syndromes (ERN GENTURIS); ^5^Centre for Human Genetics, Univ Leuven, Leuven, Belgium; ^6^Laboratory of Human Genetics, GIGA-Institute, University of Liège, Liège, Belgium; Center of Genetics, University Hospital (CHU), Liège, Belgium; European Reference Network on Genetic Tumour Risk Syndromes (ERN GENTURIS); ^7^Clinical Genetics Department, University Hospital of Ghent, Ghent, Belgium; European Reference Network on Genetic Tumour Risk Syndromes (ERN GENTURIS); ^8^Service de Génétique Oncologique, Institut Curie, Paris, France; ^9^Service de Génétique Oncologique, Institut Curie, Paris, France; European Reference Network on Genetic Tumour Risk Syndromes (ERN GENTURIS); ^10^Département de Génétique, GH Pitié- Salpêtrière, Paris, France; ^11^Institute of Human Genetics, University Hospital Bonn, Bonn, Germany; European Reference Network on Genetic Tumour Risk Syndromes (ERN GENTURIS); ^12^Department of Internal Medicine I, University Hospital Bonn, Bonn, Germany; National Center for Hereditary Tumor Syndromes, University Hospital Bonn, Bonn, Germany; ^13^TU Dresden, Medical Faculty CGC, Institute for Clinical Genetics, Dresden, Germany; ^14^TU Dresden, Medical Faculty CGC, Institute for Clinical Genetics, Dresden, Germany; European Reference Network on Genetic Tumour Risk Syndromes (ERN GENTURIS); ^15^Medizinische Klinik und Poliklinik IV, Klinikum der Universität München, Munich, Germany; Medizinisch Genetisches Zentrum, Munich, Germany; European Reference Network on Genetic Tumour Risk Syndromes (ERN GENTURIS); ^16^Biosciences Laboratory, Istituto Scientifico Romagnolo per lo Studio e la Cura dei Tumori (IRST) IRCCS, Meldola, Italy; ^17^Department of Biology and Biotechnology “Lazzaro Spallanzani; ^18^Institute of Genomic Medicine, Catholic University of the Sacred Heart, Rome, Italy; ^19^Genomed Diagnósticos de Medicina Molecular, S.A., Lisbon, Portugal; ^20^Department of Molecular Diagnostics, Institute of Oncology Ljubljana, Ljubljana, Slovenia; European Reference Network on Genetic Tumour Risk Syndromes (ERN GENTURIS); ^21^Department of Molecular Diagnostics, Institute of Oncology Ljubljana, Ljubljana, Slovenia; ^22^Clinical Genetics Unit and CIMA LAB Diagnostics, University Clinic of Navarra, Pamplona, Spain; ^23^Molecular Genetics Laboratory, Elche University Hospital, Elche, Spain; ^24^Hereditary Cancer Program, Catalan Institute of Oncology, IDIBELL and CIBERONC, Barcelona, Spain; European Reference Network on Genetic Tumour Risk Syndromes (ERN GENTURIS); ^25^Hereditary Cancer Programme, Catalan Institute of Oncology (ICO), Bellvitge Institute for Biomedical Research (IDIBELL) and Biomedical Research Institute (IDIBGI), Barcelona-Girona, Spain; European Reference Network on Genetic Tumour Risk Syndromes (ERN G); ^26^Hospital Vall d’Hebron and Universitat Autonoma de Barcelona, Barcelona, Spain; European Reference Network on Genetic Tumour Risk Syndromes (ERN GENTURIS); ^27^Molecular Diagnostics Laboratory, Fundación Rioja Salud, Logroño, Spain; ^28^Human Genetics, Radboud University Medical Center, Nijmegen, Netherlands; European Reference Network on Genetic Tumour Risk Syndromes (ERN GENTURIS); ^29^Medical Genetics, University of Cambridge, Cambridge, UK; ^30^MRC Cancer Unit, Hutchison-MRC Research Centre, University of Cambridge, Cambridge, UK; ^31^Division of Evolution and Genomic Sciences, University of Manchester, Manchester, Greater Manchester, UK; NW Genomic Laboratory hub, Manchester Centre for Genomic Medicine, Manchester, UK; European Reference Network on Genetic Tumour Risk Syndromes (ERN G); ^32^Manchester Centre for Genomic Medicine, Manchester, UK; European Reference Network on Genetic Tumour Risk Syndromes (ERN GENTURIS); ^33^St Georges NHS Foundation Trust, London, UK; ^34^Karolinska Institutet and Department of Clinical Genetics, Karolinska Univ Hospital, Stockholm, Sweden; ^35^Karolinska Institutet and Department of Clinical Genetics, Karolinska Univ Hospital, Stockholm, Sweden; European Reference Network on Genetic Tumour Risk Syndromes (ERN GENTURIS); ^36^Biocant- Transfer Technology Association, Cantanhede, Portugal; ^37^Biocruces Health Research Institute, Barakaldo, Spain; ^38^Ctr Human Gen, Institut de Pathologie et de Génétique, Gosselies, Belgium; ^39^Medical Genetics, University of Cambridge, Cambridge, UK; European Reference Network on Genetic Tumour Risk Syndromes (ERN GENTURIS); ^40^Expression Regulation in Cancer, i3S & Ipatimup & FMUP & ERN-GENTURIS & CDH1-ClinGen working Group, Porto, Portugal

Germline *CDH1* Pathogenic (P) and Likely Pathogenic (LP) variants are actionable variants in Hereditary Diffuse Gastric Cancer (HDGC), predisposing for diffuse gastric cancer (DGC) and lobular breast cancer (LBC). While asymptomatic P/LP variant carriers undergo intensive stomach/breast surveillance and prophylactic surgery to avoid disease, the clinical management of carriers of *CDH1* variants of unknown significance (VUS) remains unsolved. Almost all missense variants are classified as VUS, while predicted truncating variants and large rearrangements are more often classified as P/LP. We aimed to study, at using genotype-phenotype analysis, *CDH1*-associated disease spectrum and age-of- disease onset to improve VUS classification. Among European Reference Network ERN-GENTURIS collaborators from 10 European countries, we collected and registered phenotypes from 854 families carrying *CDH1* germline variants. We classified all variants according to the *CDH1*-ACMG clinical classification. From 854 families, 194 carried truncating variants, from which 88% were P/LP and 12% were VUS, 71% fulfilling clinical criteria. Among 607 phenotypes from P/LP variants, DGC was the most prevalent phenotype (37%) followed by LBC (10%), both at an average of onset (AOO) below 51. Families carrying truncating-P/LP and truncating-VUS showed an equivalent phenotype distribution and frequency of fulfillment of clinical criteria, suggesting a potentially clinical actionability for these VUS, that we called ‘hot VUS’. In contrast, in families carrying missense-VUS (433 probands + relatives), 92% lacking clinical criteria, DGC and LBC together accounted for < 10% of the phenotypes. This phenotype distribution overlapped that of benign/likely benign carrier families, and for this, they were called ‘cold VUS’. Our study encloses the largest dataset of *CDH1* variant carriers ever studied for genotype-phenotype analysis and the first formally demonstrating potential differences in clinical actionability of *CDH1* VUS, supported by phenotypical presentations. *CDH1* missense VUS are most likely cold- VUS, that rather than causal of HDGC, may represent important phenotype modifiers.

**OP5 –** EFFECT OF *FMR1* ALLELIC COMPLEXITY COMBINATIONS IN X-CHROMOSOME INACTIVATION SKEWING: TESTS ON COHORTS OF INFERTILE FEMALES AND OOCYTE DONORS

Bárbara Rodrigues^1^; Emídio Vale-Fernandes^2^; Nuno Maia^1^; Flávia Santos^1^; Isabel Marques^1^; Rosário Santos^1^; António J A Nogueira^3^; Paula Jorge^1^

^1^Molecular Genetics Unit, Centro de Genética Médica Jacinto Magalhães (CGM), Centro Hospitalar Universitário do Porto (CHUP); Unit for Multidisciplinary Research in Biomedicine (UMIB), Institute of Biomedical Sciences Abel Salazar (ICBAS), UP, Porto, PT; ^2^Centre for Medically Assisted Procreation/PublicGameteBank, Centro Materno-Infantil do Norte Dr. Albino Aroso (CMIN), Centro Hospitalar Universitário do Porto (CHUP); UMIB-ICBAS-UP, Porto, PT; ^3^Center for Environmental and Marine Studies (CESAM), Department of Biology, UA, Aveiro, PT

**Introduction –***FMR1* gene premutations have been implicated in fragile X-associated primary ovarian insufficiency and, more recently, specific normal-sized alleles were implicated in ovarian function impairment, although only some studies support that these “low zone” (CGGn < 26) alleles exert negative effects. We speculate that disregard of AGG interspersion patterns may explain the contradicting results. To better understand the role of AGGs, we developed a mathematical model that depicts the *FMR1* repeat substructure of each allele. The outcome, designated *allelic score*, measures the allelic complexity including AGG burden. We evaluated the *FMR1 allelic score* and the X-chromosome inactivation (XCI) pattern in cohorts of infertile females and oocyte donors.

**Methods –** Cohorts of 25 infertile females and 25 oocyte donors were used. *Allelic score* was calculated for each allele. XCI was analysed by HUMARA using *Hha*I a methylation-specific endonuclease (methylation is determined using allele peak heights and normalized to the corresponding undigested height).

**Results –***Allelic scores* ranged from 23 to 825 and from 11 to 229 in the infertile and donors cohorts, respectively. *FMR1* sub-genotypes distribution revealed that 32% of the infertile females had an allele with CGG > 34 and 36% had CGG < 26, as opposed to 16% and 44%, respectively, in the oocyte donor cohort. The number of samples with XCI skewing [90:10] was higher among infertile females (n = 5, 20%) as compared to that of oocyte donors (n = 2, 8%). The correlation between the *allelic score* of each allele revealed that these two samples belong to the *dissimilar* group, which is enriched with “low zone” heterozygous samples.

**Conclusion –** Although exploratory, our study suggests that the AGGs (decoded in the *allelic score*) relate to the XCI pattern. XCI skewing is regarded as a putative mechanism underlying ovarian dysfunction, explaining the incomplete penetrance of this impairment. Replication of these results is mandatory using larger cohorts. Application of our mathematical model and testing *FMR1 allelic scores* will confer a different perspective on *FMR1* characterization and its impact in female fertility.

**OP6 –** CELL-FREE DNA: A TOOL FOR THE DIAGNOSIS AND FOLLOW-UP OF ORAL CANCER?

Ivana Martins^1^; Leonor Barroso^2^; Inês Tavares^1^; Luís M. Pires^1^; Francisco Marques^3^; Joana B. Melo^1,4,5,6^; Isabel M. Carreira^1,4,5,6^; Ilda P. Ribeiro^1,4,5,6^

^1^Cytogenetics and Genomics Laboratory, Faculty of Medicine, University of Coimbra, Portugal; ^2^Maxillofacial Surgery Department, Coimbra Hospital and University Centre, CHUC, EPE, Portugal; ^3^Department of Dentistry, Faculty of Medicine, University of Coimbra, Portugal; ^4^Cytogenetics and Genomics Laboratory, Faculty of Medicine, University of Coimbra, Portugal; ^4^iCBR-CIMAGO – Coimbra Institute for Clinical and Biomedical Research-Centre of Investigation on Environment, Genetics and Oncobiology, Faculty of Medicine, University of Coimbra, Portugal; ^5^CIBB – Centre for Innovative Biomedicine and Biotechnology, University of Coimbra, Portugal; ^6^CACC – Clinical Academic Center of Coimbra, Coimbra, Portugal

**Introduction:** Liquid biopsies are a complementary or alternative approach to tissue biopsies for the diagnosis and monitoring of cancer in a less invasive way. These consist in the detection of tumor-released components, such as circulating tumor DNA (ctDNA), in biofluids. Oral cancer patients are usually diagnosed in late stages and, besides that, they are frequently affected by recurrence, which contributes to their low survival. However, the clinical impact of liquid biopsies in this type of cancer is limited, requiring further studies. Thus, the aim of this pilot study was to explore the potential of liquid biopsies for the diagnosis and follow-up of oral cancer patients.

**Methodology:** A total of 59 and 57 samples of plasma and urine were collected from 16 oral cancer patients before and at several timepoints after treatment initiation. Plasma and urine samples from controls were also collected. After cell- free DNA (cfDNA) isolation, concentrations were determined, compared between patients and controls, and monitored throughout the patients’ clinical course. Furthermore, the ctDNA mutational profile of 2 patients was compared with the profile of corresponding tumor tissues and monitored at different timepoints using Next Generation Sequencing (NGS). The results were correlated with the patients’ clinicopathological data.

**Results:** The levels of plasma cfDNA seem to increase right after treatment initiation in most patients, before decreasing. Sequencing of ctDNA revealed mutations in important genes such as *TP53* and *PIK3CA* and allowed to identify new alterations during the treatment course.

**Discussion:** Our results reveal that it is possible to isolate ctDNA from plasma and urine of oral cancer patients and that the integrated analysis of liquid and tissue biopsies allows a more comprehensive characterization of the tumor profile. Moreover, following cfDNA levels and mutational profile during treatment might have value to monitor disease and treatment response. More studies including more patients and a longer follow-up period will be important to confirm the potential of liquid biopsies for the diagnosis and monitoring of this cancer.

**CLINICAL CASES**

**OP7 –** A CASE OF MOWAT-WILSON SYNDROME DUE TO A MISSENSE VARIANT – CASE REPORT AND REVIEW OF THE LITERATURE

Susana L. Ferreira^1^; Mafalda S. Melo^1^; Teresa Kay^1^; Marta Amorim^1^

^1^Serviço de Genética Médica, Centro Hospitalar Universitário de Lisboa Central

**Introduction:** Mowat-Wilson syndrome (MWS) (OMIM # 235730) is a rare cause of intellectual disability (ID) (moderate/severe) with speech impairment but comprehension preserved and typical facial features. Other congenital anomalies commonly associated are seizures, microcephaly, genitourinary abnormalities, congenital heart defects, Hirschsprung disease or chronic constipation, and growth restriction. In most cases, MWS results of *de novo* heterozygous truncating *ZEB2* variants or deletions encompassing the gene which are responsible for the characteristic phenotype. However, in less than 5% of reported cases, atypical or milder forms of MWS spectrum are the result of missense, splice site or in-frame variants. In these cases, milder degrees of ID and some suggestive features can be present, with or without other associated congenital malformations.

**Case report:** A 5-year-old male presented with development delay, dyspraxia, facial dysmorphisms (synophrys, open-mouth expression, uplifted earlobes and prominent chin), hypospadias and history of constipation, stature and weight above 95th percentile and head circumference between 50-75th percentile. EEG showed parietal epileptiform focus activity without seizures, so far. Karyotype, FRAXA and array-CGH were normal. NGS panel for ID presented a missense variant in *ZEB2* c.3141G > C (p.(Arg1047Ser)), initially classified as of uncertain significance. Analysis concluded that it is a deleterious variant and parental segregation confirmed it is *de novo*. The reclassification of this missense variant as likely pathogenic established the diagnosis of MWS with an atypical phenotype (including macrosomia, not previously described).

**Conclusion:** MWS has a characteristic phenotype, distinct facial features and it is genetically homogeneous. Rarer missense variants result in atypical/milder phenotypes and data from these patients can help understand the range of this syndrome. This case continues to expand the spectrum of MWS and highlights that it can be a less obvious diagnosis. From a practical standpoint, it provided an appropriate genetic counseling and better surveillance to this patient and family.

**OP8 –** CUBILIN VARIANTS IN PATIENTS WITH CHRONIC KIDNEY DISEASE INCLUDING END- STAGE

Rita Quental^1^; Sofia Quental^2^; Ana Grangeia^1^; Sérgio Castedo^3^; João P. Oliveira^4^

^1^Serviço de Genética Médica, Centro Hospitalar Universitário de São João, Porto, Portugal; ^2^Ipatimup - Instituto de Patologia e Imunologia Molecular da Universidade do Porto, Porto, Portugal; i3S - Instituto de Investigação e Inovação em Saúde, Universidade do Porto, Porto, Portugal; ^3^Serviço de Genética Médica, Centro Hospitalar Universitário de São João, Porto, Portugal; Ipatimup - Instituto de Patologia e Imunologia Molecular da Universidade do Porto, Porto, Portugal; i3S - Instituto de Investigação e Inovação em Saúde, Universidade; ^4^Serviço de Genética Médica, Centro Hospitalar Universitário de São João, Porto, Portugal; i3S - Instituto de Investigação e Inovação em Saúde, Universidade do Porto, Porto, Portugal

Cubilin mediates tubular reabsorption of many proteins filtered by glomerular capillary, such as albumin. Biallelic pathogenic variants in *CUBN* gene, coding for cubilin, cause Imerslund-Gräsbeck Syndrome characterized by megaloblastic anemia and variable proteinuria. More recently, *CUBN* deleterious variants, particularly in the C- terminal domains, have been associated with chronic isolated proteinuria. So far, the clinical evolution of these patients suggests an indolent course, although this remains unclear.

We report 4 patients (3 families) with suspected hereditary renal disease and persistent proteinuria. In two patients, proteinuria was incidentally detected in infancy whereas in the other two (siblings) diagnosis was made in adulthood, one of them with end-stage kidney disease as the inaugural manifestation. A next-generation sequencing approach was used to study genes associated with renal disorders. In the two siblings, only shared variants have been searched for. In all patients we have detected *CUBN* variants potentially implicated in the phenotype. Pt 1 presented the c.6928_6934del p. (Glu2310Cysfs∗3) in compound heterozygosity with c.5597C > T p.(Pro1866Leu). Pt 2 was a compound heterozygote for c.6007A > T p.(Arg2003∗) and c.5840C > A p.(Ser1947Tyr). Joint analysis of the siblings (Pts 3 and 4) revealed variants c.1512C > G p.(Ile504Met) and c.7379T > C p.(Ile2460Thr). In this case, the patients’ parents were not available for genetic study.Four variants herein reported have not been described before.

The characteristics of Pts 1 and 2 are in accordance with previous descriptions of CUBN-affected individuals: were diagnosed in infancy, present a stable clinical state, and have *CUBN* variants in the C-terminal domains. In contrast, Pts 3 and 4 have a more severe kidney disease, an uncommon finding for *CUBN* patients. One of their variants is in one of the N-terminal domains. This, however, does not exclude its clinical relevance, as similar variants have been described as a cause of proteinuria when combined with a C-terminal pathogenic alteration. In this family, additional studies are being performed to investigate the potential pathogenicity of these variants.

**OP9** – *TBCD*-RELATED ENCEPHALOPATHY: A PATIENT WITH A SEVERE NEONATAL PRESENTATION

Marta P. Soares^1^; Márcia Rodrigues^1^; Ana Berta Sousa^1^

^1^Serviço de Genética, Hospital de Santa Maria, Centro Hospitalar Universitário Lisboa Norte, Centro Académico de Medicina de Lisboa, Lisbon, Portugal

**Introduction:** Neonatal encephalopathy is a heterogeneous condition characterized by respiratory insufficiency, decreased consciousness, tone and reflexes, and seizures. It can be caused by hypoxia-ischemia, sepsis, metabolic, thromboembolic and genetic disorders. Prognosis is poor, including developmental delay (DD), epilepsy, cerebral palsy, and death.

**Methods:** We describe a female infant with severe encephalopathy born to non-consanguineous parents with no relevant family history. Pregnancy was complicated by fetal akinesia at 36 weeks+, leading to emergency C- section. She presented with respiratory depression and was resuscitated and ventilated (Apgar score 2/4/4). Neurological examination revealed severe neurological impairment (miosis, no response to painful stimuli, and tetraplegia). Neuroimaging showed dilated lateral ventricles, hypoplastic cerebellar vermis, enlarged cisterna magna, and diffuse subcortical and cortical atrophy (CA). Muscle biopsy revealed small muscle fibres and PAS- positive inclusions. Further investigation (electromyography, metabolic studies, genetic testing for spinal muscular atrophy, Steinert myotonic dystrophy and a NGS multigene panel for hereditary myopathies) was normal. Her clinical condition deteriorated with death at 3 months.

**Results:** Whole exome sequencing (trio approach) detected two compound novel heterozygous variants in *TBCD*: c.2917C > T, p.(Leu973Phe) and c.2828_2830del, p.(Gly943del). *TBCD* encodes one of five co-chaperones required for microtubule assembly dynamics. Biallelic pathogenic variants in *TBCD* cause PEBAT (early-onset progressive encephalopathy with brain atrophy and thin corpus callosum). PEBAT (MIM #617193) is characterized by DD/developmental regression, hypotonia and/or spasticity, epilepsy, CA, secondary hypomyelination and thin corpus callosum.

**Discussion:** Both variants are located in a functional domain, were not found in controls, and are highly specific for the phenotype, allowing classification as likely pathogenic. Molecular diagnosis allowed proper genetic counselling, including recurrence risk (25%) and informed reproductive choices. The couple was referred for pre- implantation genetic diagnosis.

**POSTER PRESENTATION**

**BASIC RESEARCH**

**P1 –** GENETICS AS PART OF HEALTH SCIENCE EDUCATION: REFLECTING ON A CHANGING APPROACH

Olga Amaral^1^; Filipa Ferreira^1^

^1^Instituto Nacional de Saude Ricardo Jorge

Genetics goes beyond the human species and this should be the paramount power of genetics education. The current wave of interest in Science should be the force towards a change in the way information reaches the public. The prior underestimation of the understanding capabilities of the student and non-student populations resulted in a loss of engagement in science and might lead to misinformation. In the past decades, INSA has had an approach to communication in Health Science that depends on strong views and areas of expertise, various programmed activities and, as in research, it depends on expectations. Along the line of community involvement, GENEtic.COMunication (GENE.COM) is a project submitted to a competitive call launched by Ciencia Viva. With that project, the focus is on a niche of knowledge with great impact on Health, in society, as well as on personal and public budgets: the area of the rare genetic metabolic diseases. The project intended to contribute to the dissemination of information about Human Genetics, particularly in the area of rare genetic diseases. The aim was to communicate knowledge in an easily understandable way; inform health professionals, patients, students, teachers, as well as patient associations. This contact could provide the establishment of a dialogue between professionals working in the field and different publics. Furthermore, the switch to virtual tools relies on easier access to the information while implying diminished associated costs, such as transportation, time and space limitations. The problem may be how to make passive learning more engaging. As we know, assessment drives learning and there are simple and quick ways to assess the efficacy of the “take home message”, which may improve engagement and provide the needed feedback. Attempts to contribute to a more informed society should be disclosed and supported as they can lead to more positive attitudes and empowered choices. By providing digital contents in a way that they can be followed by various target audiences we also aim at reaching audiences that would not typically seek us.

**P2 –** A FIRST STEP TO OPEN THE NEURONAL BOX OF GAUCHER DISEASE: NEURAL PROGENITOR CELLS

Diogo Ribeiro^1^; Ana Joana Duarte^1^; Renato Santos^2^; Olga Amaral^3^

^1^Instituto Nacional de Saúde Doutor Ricardo Jorge (INSA, IP), Departamento de Genética Humana, Unidade de Investigação e Desenvolvimento, Porto; ICBAS, Universidade do Porto, Porto; CECA, ICETA, Universidade do Porto, Porto; ^2^Instituto Nacional de Saúde Doutor Ricardo Jorge (INSA, IP); ^3^Instituto Nacional de Saúde Doutor Ricardo Jorge (INSA, IP), Departamento de Genética Humana, Unidade de Investigação e Desenvolvimento, Porto; CECA, ICETA, Universidade do Porto, Porto.

Gaucher disease (GD) is a common lysosomal storage disorder usually associated with defective activity of the lysosomal glucocerebrosidase (encoded by GBA1, OMIM#606463). Of all known types of GD, types 2 and 3 GD are well known as neuronopathic forms.

This work focuses on the differentiation and gene expression characterization of neural progenitor cells obtained from human induced pluripotent cells (hiPSCs) reprogrammed from type 3 GD (GD3) fibroblasts.

GD3 patient fibroblasts (from an international cell bank) were cultured and reprogramed as previously described (https://doi.org/10.1016/j.scr.2019.101595). The resulting hiPSCs were differentiated into pre-neuronal cells and, at this stage, they were examined.

The cDNA obtained from GD3 fibroblasts, GD3 hiPSCs and resulting neural progenitor cells was used in Real Time qPCR for gene expression analysis. We used FAM probes for genes encoding cytoskeleton control proteins, transcription factors involved in stemness, transcription factor involved in growth and development and genes involved in neurogenesis.

Regarding the control genes expression, we found that there was no difference in the expression of the *GAPDH* gene between GD3 hiPSCs and GD3 neural progenitor cells. We also verified that the *TUBB3* gene expression was very similar in GD3 fibroblasts and GD3 hiPSCs, but we saw decreased expression of this gene in GD3 neural progenitor cells. The *OCT4* and *SOX2* gene expression in GD3 neural progenitor cells was comparable to the gene expression in GD3 patient fibroblasts. The *ZEB2* gene expression was higher in GD3 fibroblasts than in GD3 neural progenitor cells. The gene expression behavior of all neurogenesis genes (*NES*, *MAP2* and *OTX2*) was similar but higher expression was observed in GD3 hiPSCs than in GD3 neural progenitor cells.

With this work, we can conclude that, when working with hiPSCs in the process of creating disease-specific cell models it is most important to carry out a general gene expression characterization of the different cell lines involved in all stages.

This work was financed by FCT (PTDC/BIM-MEC/4762/2014) and INSA-DGH.

**P3 –***IN SILICO* DETECTION OF COPY-NUMBER VARIANTS IN HEREDITARY CANCER SYNDROMES USING NGS PANEL DATA AND PANELCN.MOPS

Pedro Rodrigues^1^; Vanessa Carvalho^2^; Patrícia Theisen^3^; Inês Franscisco^4^; Bruno Filipe^4^; Patrícia Silva^4^; Catarina Sílvia^5^; Dina Carpinteiro^5^; Luís Vieira^5^; Cristina Albuquerque^4^; João Gonçalves^5^

^1^Human Genetics Department, National Institute of Health Dr Ricardo Jorge, Lisbon, Portugal; ^2^Human Genetics Department, National Institute of Health Dr Ricardo Jorge, Lisbon, Portugal; University of Trás-os-Montes and Alto Douro, Vila Real, Portugal; ^3^Human Genetics Department, National Institute of Health Dr Ricardo Jorge, Lisbon, Portugal; ^4^Molecular Pathobiology Research Unit, Portuguese Institute of Oncology Francisco Gentil, Lisbon, Portugal; ^5^Human Genetics Department, National Institute of Health Dr Ricardo Jorge, Lisbon, Portugal; Center for Toxicogenomics and Human Health, Nova Medical School, Lisbon, Portugal.

**Introduction**: Genetic diagnosis of Hereditary Cancer Syndromes (HCS) using next-generation sequencing (NGS) is an outstanding technology to detect single- nucleotide variants (SNVs) and small deletion/insertions, with faster turnaround time and lower costs compared to Sanger sequencing. However, detection of copy-number variants (CNVs) from NGS data remains a challenge and multiplex-ligation dependent probe amplification (MLPA) is routinely used to screen for CNVs. The aim of this work was to evaluate a CNV detection tool, panelcn.MOPS(a), using NGS data from Hereditary Coloretal Cancer (HCRC) and Hereditary Breast/Ovarian Cancer (HBOC) samples.

**Methods:** 304 samples (117 HBOC and 187 HCRC, with 16 CNVs previously detected by MLPA) were analyzed using the TruSight Cancer (TSC) panel and MiSeq platform (Illumina). Panelcn.MOPS (designed for TSC data) was used for CNV detection in 24 genes (HBOC and HCRC related), comprising 379 exons/Regions Of Interest (ROI).

**Results/Discussion:** As panelcn.MOPS includes several quality control (QC) steps, it was not possible to analyze all 379 ROI, because some were marked with low quality (LQ) due to high GC content or issues related with enrichment or mappability of reads. Among the 304 samples, 272 fulfilled all the QC criteria (360 ROI were analyzed), 29 were tagged in the QC last step (with 352 ROI analyzed), and 3 samples were excluded due to overall LQ. CNVs were called in 37 samples (8 HBOC and 29 HCRC) by panelcn.MOPS, 15/16 CNVs had been previously validated by MLPA. One CNV (*PMS2* exon 14 deletion) was not detected because it matched to one ROI excluded for analysis due to LQ. 22 additional CNVs were detected (3 already validated by MLPA, 19 awaiting confirmation). When no SNVs are identified in HCS by NGS, MLPA is performed, but this method is time consuming and costly. Despite some limitations of NGS data, panelcn.MOPS proved to be highly useful as a first CNV screening test, decreasing the number of MLPA tests required in a diagnostic setting.

**(a)**-Povysil G et al 2017, DOI: 10.1002/humu.23237.

**Acknowledgements:** FCT/MCTES, Toxicogenomics and Human Health (UIDB/00009/2020).GenomePT project (POCI-01-0145-FEDER- 022184).

**P4 –** TRANSLATIONAL REGULATION OF THE HUMAN PERK BY UPSTREAM OPEN READING FRAMES

Rafael Fernandes^1^; Pedro Lopes^2^; Luísa Romão^1^

^1^Instituto Nacional de Saúde Doutor Ricardo Jorge; University of Lisboa, Faculty of Sciences, BioISI - Biosystems & Integrative Sciences Institute; ^2^Instituto Nacional de Saúde Doutor Ricardo Jorge

Upstream open reading frames (uORFs) are *cis*-acting elements located within the 5’ leader sequence (5’UTR) of transcripts, which can regulate translation of the correspondent main open reading frame (mORF). During endoplasmic reticulum (ER) stress, the accumulation of unfolded proteins activates the ER-resident PKR-like ER kinase (PERK), which results in phosphorylation of eIF2á to inhibit global mRNA translation, while allowing the selective uORF-mediated translation of downstream effectors responsible for stress resolution or, ultimately, cell death. The dual role of PERK in regulating cell fate was implicated in human diseases, like diabetes, neurodegenerative disorders and cancer. Moreover, mutations in the EIF2AK3 gene (encoding PERK) were associated to the rare genetic disease, Wolcott-Rallison Syndrome (WRS). In this work, we aimed to study the translational regulatory role of 5 AUG- and 3 non-AUG- uORFs identified in the *PERK* 5’UTR and assess its biological relevance. While uORF2 and the non-AUG- uORFs 5, 6 and 7 (numbered according to their distance to the 5’ end of the mRNA) do not seem to have a regulatory role, uORF1, uORF3, uORF4 and uORF8 together present a strong repressive effect over mORF translation in basal conditions. Curiously, we found that when PERK is overexpressed, it leads to the spontaneous activation of a portion of PERK in the absence of any stress stimulus, possibly highlighting the biological relevance of its uORF- mediated translational regulation. Conversely, during ER stress, increased bypass of uORF1 results in a modest degree of translational de-repression, which may help to counterbalance the increased rate of PERK protein turnover observed in these conditions. We also observed that alteration of the PERK uORFs by mutations found in WRS patients modify mORF expression, providing a possible link to the disease. Altogether, we highlight the importance of including 5’UTRs in the screening of disease-related mutations and the necessity of functional studies to assess their role in pathogenesis.

**P5 –** AN ALGORITHM THAT PREDICTS THE EFFECT OF miRNAS TRANSPORTED BY EXTRACELLULAR VESICLES IN RECIPIENT CELLS

Joaquin Jurado Maqueda^1^; Sara Rocha^2^; Carla Oliveira^3^

^1^BIOINF2BIO, LDA. i3S - Instituto de Investigação e Inovação em Saúde, Universidade do Porto & Ipatimup – Institute of Molecular Pathology and Immunology of the University of Porto; ^2^i3S - Instituto de Investigação e Inovação em Saúde, Universidade do Porto & Ipatimup – Institute of Molecular Pathology and Immunology of the University of Porto; ^3^BIOINF2BIO, LDA. i3S - Instituto de Investigação e Inovação em Saúde, Universidade do Porto & Ipatimup – Institute of Molecular Pathology and Immunology of the University of Porto.Faculty of Medicine, University of Porto.

Extracellular vesicles (EVs) are membrane-coated nanovesicles that represent an important mode of intercellular communication by serving as vehicles for transfer of biomolecules between cells. Notably, EVs can play an essential role in immune response, tumour promotion and progression and tissue regeneration. Although it is established that the cargo of EVs produces a transcendent biological effect in recipient cells uptaking them, there is currently no way to predict that effect. We aimed at developing a pipeline to predict and explore the effect of miRNAs transported by EVs in recipient cells, namely protein expression profile and related networks.

A dataset of differentially expressed EV-miRNAs between two biological conditions was used as input of the workflow. miRNA-target interactions (MTIs) were downloaded from miRTarBase. Only miRTarBase strong targets (proven by reporter assays) were considered. The correlation between MTIs and differentially expressed miRNAs was represented in a network using the R package *igraph*. Cytoscape was used to depict the resulting network.

We successfully created an algorithm that correlates miRNA-target interactions with related expression. The results of this correlation allowed building a network where driver microRNAs appear linked to their protein targets, according to the real expression of miRNAs and predicted expression modulation of proteins targets. This visual interface allows to identify the miRNA-protein nodes that are most likely impacted by the uptake of a specific EV cargo.

Our approach responds to a wide research need related to the prediction of EVs-miRNA mediated effect in recipient cells. This novel bioinformatic pipeline highlights the most likely set of proteins or protein networks to be modified after interacting with miRNAs transported by EVs, opening the way for exploring not only the EV-cargo but mainly its biological effects in any cell type.

**Acknowledgements**: TRAIN-EV has received funding from the EU's Horizon 2020 research and innovation programme under the Marie Sklodowska-Curie grant agreement No 722148.

GenomePT–National Laboratory for Genome Sequencing and Analysis | POCI-01-0145-FEDER-022184

**P6 –** MOLECULAR CHARACTERIZATION OF REGULATORY ELEMENTS IDENTIFIED IN THE 5’- AND 3’- UTRS OF *BMPR1A* TRANSCRIPTS

Joel Silva^1^; Márcio Simão^2^; Leonor Cancela^3^

^1^Centre of Marine Sciences (CCMar), Universidade do Algarve; ^2^Department of Biomedical Sciences and Medicine (DCBM); Centre of Marine Sciences (CCMAR); Universidade do Algarve; ^3^Department of Biomedical Sciences and Medicine (DCBM); Centre of Marine Sciences (CCMAR); Algarve Biomedical Centre (ABC) and Centre for Biomedical Research (CBMR); Universidade do Algarve

Bone morphogenetic proteins are an important group of cytokines involved in several processes including cell cycle regulation and differentiation. Their signal is transduced through serine/threonine kinase receptors, BMPRI (A or B) and BMPRII. BMP signalling is involved in bone metabolism and depends on the balance between bone formation and resorption. BMPRIA is a mediator in bone metabolism and subjected to several levels of regulation. The objective of this study was to characterize Mus musculus Bmpr1a 5’- and 3’-UTRs regulatory regions, including upstream open reading frames (uORFs) that regulate the translation efficiency, polyadenylation sites and constitutive decay element (CDE), involved in transcript stability.

For molecular validation of regulatory elements identified in silico, the sequences of 5’- and 3’-UTRs containing uORFs and CDE signatures were amplified, cloned into a reporter luciferase plasmid (pGL3) and transfected into HEK293 cells to determine their impact in luciferase transcription.

The analysis of the available Bmpr1a transcripts allowed the identification of three 5’-UTR uORFs. Two different 5’ UTR lengths were analysed and results showed that Bmpr1a expression was affected in constructs with a longer 5’-UTR version. The impact of uORFs on Bmpr1a expression suggests that expression of 5’-UTR with different lengths may affect the fidelity of ribosome translation under different cellular conditions.

Analysis of the Bmpr1a 3’-UTR identified a putative conserved CDE. Two versions of this region were inserted into the 3’-end of a reporter luciferase plasmid: a shorter version without CDE, identified from a pre-osteoblast MC3T3 cells; and a longer version including a CDE, identified from a cDNA library of mouse liver tissue. The results obtained indicate that Bmpr1a transcripts are subject to a complex regulation depending on the cellular context. In proliferating MC3T3 cells, the 3’- UTR identified was shorter and should favour Bmpr1a translation. Longer versions of 3’-UTR identified in liver tissue suggest a distinct Bmpr1a regulation in differentiated hepatocytes. Additional studies are required to understand its impact on translation.

**P7 –** INTERPLAY BETWEEN GLYCEMIA AND THE GENETICS OF *ENOS* AND *ACE* GENES FOR THE SUSCEPTIBILITY TO THE ONSET AND DEVELOPMENT OF HYPERTENSION ON THE PORTUGUESE POPULATION

Laura Aguiar^1^; Joana Ferreira^2^; Andreia Matos^2^; Mário Rui Mascarenhas^3^; Luiz Menezes Falcão^4^; Paula Faustino^5^; Manuel Bicho^2^; Ângela Inácio^1^

^1^Instituto de Investigação Científica Bento da Rocha Cabral; ^2^Laboratório de Genética, Faculdade de Medicina da Universidade de Lisboa; ^3^Clínica de Endocrinologia, Diabetes e Metabolismo de Lisboa, Lisboa, Portugal; ^4^Departamento de Medicina Interna, Hospital de Santa Maria; ^5^Departamento de Genética Humana, Instituto Nacional de Saúde Doutor Ricardo Jorge

**Introduction:** Hypertension is a multifactorial condition of anthropometric, physiologic, metabolic, genetic, and environmental nature. In Portugal, the mean prevalence of hypertension in the population is 45.5%.

**Objective:** The aim of this study is to evaluate the contribution of anthropometric, physiological, and genetic factors (e*NOS* and *ACE*) to the development of hypertension in a Portuguese population.

**Methods:** A case-control study was conducted in a sample of 377 individuals, 243 hypertensives, and 134 normotensives. The polymorphic analyses of intron 4 VNTR in the *eNOS* gene and the insertion/deletion (I/D) in *ACE* gene were performed by polymerase chain reaction (PCR).

**Results:** High body mass index (BMI) values, high glycemia levels, and the 4a allele of the *eNOS* were associated with hypertension. Among the hypertensive group, the allele 4a (*eNOS*) was associated with high levels of HbA1c, and the D allele (*ACE*) with glycemia.

**Conclusion:** Our results highlight the contribution of *eNOS* and *ACE* genes as important players for the onset and development of hypertension in the Portuguese population. We believe that a combinatory clinical approach including the traditional anthropomorphic and physiological parameters together with genetic studies can be more elucidative in establishing a susceptibility profile on multifactorial conditions as hypertension.

**P8 –** RARE SUBSTITUTION IN THE 3’UTR OF THE *CYP21A2* GENE ASSOCIATED WITH A MILD NON- CLASSIC FORM OF CONGENITAL ADRENAL HYPERPLASIA

Susana Gomes^1^; Iris Pereira-Caetano^1^; Catarina Limbert^2^; Rosa Pina^2^; Diana Antunes^3^; Joana Rosmaninho-Salgado^4^; Luisa Cortez^5^; Catarina Senra Moniz^6^; Alberto Galvão Telles^7^; João Gonçalves^1,8^

^1^Human Genetics Department, Instituto Nacional de Saúde Doutor Ricardo Jorge, Lisboa, Portugal; ^2^Pediatric Endocrinology Service, Hospital Dona Estefânia, Lisboa, Portugal; ^3^Clinical Genetics Service, Hospital Dona Estefânia, Lisboa, Portugal; ^4^Clinical Genetics Service, Centro Hospitalar e Universitário de Coimbra, Portugal; ^5^Endocrinology Service, Hospital Curry Cabral, Lisboa, Portugal; ^6^Endocrinology Service, Hospital do Divino Espírito Santo de Ponta Delgada, Açores, Portugal; ^7^Endocrinology, Consultório, Rua Marquês de Fronteira 113-4°, Lisboa, Portugal; ^8^ToxOmics, Faculdade de Ciências Médicas, Universidade NOVA de Lisboa, Portugal

**Introduction**: Congenital Adrenal Hyperplasia (CAH) can be due to one of seven different enzymatic defects affecting cortisol biosynthesis. CAH due to 21-hydroxylase deficiency (21-OHD) is responsible for 90 – 95% of the CAH cases. Clinical symptoms of CAH, due to 21-OHD caused by *CYP21A2* gene alterations, are directly dependent of 21-OH residual activity. CAH comprises a less severe phenotype, the non-classic (NC) form of the disease.NC associated genotypes are characterized by the presence of a mild pathogenic variant in one allele of the gene. While these variants are usually present in the coding regions (exons 1 to 10) of *CYP21A2*, rare variants in the 5’ or 3’ untranslated region (UTR) of *CYP21A2* should also be considered in a diagnostic setting.

**Methods**: Molecular analysis of *CYP21A2* requires additional precautions due to its highly homologous pseudogene. Our *CYP21A2* strategy of analysis includes a specific expand long-PCR amplification, nested PCR (covering exons 1 to 10, the flanking intronic sequences, and the 5’ and 3’ UTRs) and Sanger sequencing.

**Results/Discussion**: Here we report the results of the 3’UTR of *CYP21A2* analysis, obtained in seven patients with a clinical diagnosis of NC-CAH. The rare variant c.∗13G > A was identified in compound heterozygosity in all patients. In six of them, the second variant present in the other allele, was the mild missense variant c.844G > T p.(Val282Leu), while the other patient had the severe splicing variant c.293-13C > G (usually causing SW or SV) in the other allele. The variant c.∗13G > A was already described associated to reduced *CYP21A2* mRNA expression (25%-30%) and a decreased mRNA stability, as well as to a mild form of NC-CAH(a). The genotype/phenotype correlation in our patients, in general, are in agreement with moderate manifestations of NC 21- OHD, thus supporting previous in vitro studies and associated disease severity. This study reinforces the importance of analysis of *CYP21A2* UTRs, contributing for a better characterization of the molecular basis of mild NC- CAH due to 21-OHD, improving genetic counselling and patient's clinical management.

**Bibliography:** (a)-Menabò S *et al* (2012), doi: 10.3275/7680.

**P9 –** THE USE OF DOMINANT NEGATIVE STRATEGY IN ZEBRAFISH TO STUDY CDKL5 DEFICIENCY DISORDER

Tatiana Varela^1^; Gil Martins^1^; Débora Varela^1^; Marta Vitorino^2^; Natércia Conceição^1^; M. Leonor Cancela^1^

^1^University of Algarve/CCMAR, Faro, Portugal; ^2^Centre for Biomedical Research (CBMR), University of Algarve, Faro, Portugal

CDKL5 deficiency disorder (CDD) is an X-linked neurodevelopmental condition caused by mutations in cyclin-dependent kinase-like 5 (*CDKL5*) gene resulting in the loss-of-function of its encoded protein - a serine/threonine kinase- essential for normal brain development and function. Patients suffering from this rare disorder present early-onset seizures, which begin in the first months of life and a regression in neurological and motor development. Although a genetic cause for this condition was identified, the association between the type/location of mutations and patient's phenotype and the mechanisms responsible for its onset remain unclear. Mouse models developed to mimic CDD are unable to develop seizures, a central feature of this condition in humans. Therefore, the use of other models such as zebrafish represent an alternative tool to study this syndrome. In this work we investigated the effect of mutant CDKL5 using a dominant negative approach in zebrafish thus expecting to contribute to unveil the molecular pathways involved in the onset of CDD. For that, we produced *CDKL5* RNA harboring the mutation c.1708G > T found in a CDD patient presenting recurrent seizures, gross motor hypotonia and poor eye contact, and injected it into zebrafish embryos to look for possible phenotypes analogous to those observed in the human clinical condition. The resulting mutant protein (p.E570X) is truncated and lacks the C-terminus motifs responsible for its nuclear localization and exportation. Bioinformatic analysis confirmed the high degree of conservation between human and zebrafish *CDKL5* gene structure and protein sequence. Motor behavior was evaluated using the Zantiks equipment and a seizure behavior was observed in wildtype 5dpf larvae exposed to PTZ, a seizure-inducing drug. We are currently performing this behavioral analysis in larvae expressing the CDKL5 dominant negative form. Altogether, our results support the use of zebrafish as a valid alternative model to study CDD.

**Funding**: MDBR-19-104-CDKL5 and FCT through UIDB/04326/2020. T.V, G.M and D.V are recipients of FCT PhD grants (SFRH/BD/144230/2019, SFRG/BD/1463378/2019 and SFRH/BD/141918/2018, respectively).

**P10 –** TNFα INHIBITS SODIUM-IODIDE SYMPORTER EXPRESSION IN THYROID CELLS THROUGH NF-κB ACTIVATION

Márcia Faria^1^; Rita Domingues^2^; Francisca Paixão^3^; Maria João Bugalho^4^; Ana Luísa Silva^2^; Paulo Matos^5^

^1^Instituto Nacional de Saúde Doutor Ricardo Jorge, Lisboa, Portugal; BioISI – Biosystems and Integrative Sciences Institute, Faculdade de Ciências da Universidade de Lisboa, Portugal; Serviço de Endocrinologia, Diabetes e Metabolismo, do CHULN - Hospital; ^2^Serviço de Endocrinologia, Diabetes e Metabolismo, do CHULN - Hospital Santa Maria, Lisboa, Portugal; ISAMB - Instituto de Saúde Ambiental, Faculdade de Medicina da Universidade de Lisboa, Lisboa, Portugal; ^3^ISAMB - Instituto de Saúde Ambiental, Faculdade de Medicina da Universidade de Lisboa, Lisboa, Portugal; ^4^Serviço de Endocrinologia, Diabetes e Metabolismo, do CHULN - Hospital Santa Maria, Lisboa, Portugal; Faculdade de Medicina da Universidade de Lisboa, Lisboa, Portugal; ^5^Instituto Nacional de Saúde Doutor Ricardo Jorge, Lisboa, Portugal; BioISI – Biosystems and Integrative Sciences Institute, Faculdade de Ciências da Universidade de Lisboa, Portugal

The sodium-iodide symporter (NIS) mediates transport of iodide across the basolateral membrane of thyroid follicular cells. NIS expression in thyroid cancer (TC) cells allows the use of radioactive iodine (RAI) as a diagnostic and therapeutic tool, namely for metastatic disease. Still, a significant proportion of patients with advanced forms of TC became refractory to RAI therapy and no effective alternative therapies are available. Defective NIS expression is the main reason for impaired iodide uptake in TC and NIS downregulation has been associated with several pathways linked to malignant transformation. NF- κB signaling is among these pathways but its impact on NIS expression remains elusive. Interestingly, NIS expression can be downregulated by TNF-α, a bona fide activator of NF-κB with a central role in thyroid autoimmunity. Here, weinvestigated if the negative impact of TNF-α on NIS expression in thyroid cells involved activation of NF-κB. NIS transcript levels were analyzed by RT-qPCR in the non-transformed thyroid cell lines PCCL3 and FRTL5 and a non-radioactive iodide influx assay was used to monitor NIS-mediated iodide uptake.Treatment with TNF-α led to downregulation of TSH-induced NIS expression in both thyroid cell models. A similar effect was observed after treatment with phorbol-myristate-acetate (PMA), another inducer of NF-κB activation that acts independently of ligand-receptor specificity. TNF-α and PMA downregulation of NIS expression was reverted when NF- κB-dependent transcription was blocked, demonstrating the requirement for NF-kB activity. TNF-α and PMA were shown to inhibit NIS-mediated iodide uptake, consistent with the observed downregulation of NIS expression. Overall, our results support the involvement of NF-κB- directed transcription in the modulation of NIS expression. A better understanding of the mechanisms underlying NIS expression in the context of normal thyroid physiology may guide the development of pharmacological strategies to increase the efficiency of iodide uptake and improve the response to RAI therapy in refractory TC.

**Funding**: FCT grant PTDC/BIAMOL/31787/2017

**P11 –** REGULATION OF *ZNF687* BY THE BONE-RELATED TRANSCRIPTION FACTORS DLX5 AND HOXD13 THROUGH BIDING SITES OVERLAPPING CpG SITES

Débora Varela^1^; Natércia Conceição^1^; M. Leonor Cancela^1^

^1^University of Algarve/CCMAR, Portugal, Faro

*ZNF687* gene encodes a protein that belongs to the C2H2 zinc finger protein family. Mutations in *ZNF687* have been recently associated with severe cases of Paget's Disease of Bone (PDB), the second most common metabolic bone condition. Affected bones of PDB patients are disorganized, enlarged and deformed thus prone to fracture, due to increased bone reabsorption followed by abnormal and excessive bone formation. Although very little is known regarding ZNF687 function, *in vitro* and *in vivo* studies suggest its involvement in bone metabolism. Accordingly, it was upregulated throughout the differentiation of osteoblast and osteoclast and overexpressed in peripheral blood mononuclear cells derived from PDB-affected individuals. Thus, it its crucial to gain insights into the mechanisms involved in the *ZNF687* regulation to better understand the pathogenesis of the PDB. Therefore, the objective of this work was to obtain novel knowledge regarding *ZNF697* transcriptional and epigenetic regulation by analyzing the functionality of binding sites for the bone- related transcription factors DLX5 and HOXD13 overlapping CpG sites on *ZNF687* promoter and the effect of CpG methylation on its transcription.Through an *in silico* analysis, we identified three *ZNF687* promoter regions, upstream from exons 1A, 1B and 1C, as well as several putative binding sites for DLX5 and HOXD13, overlapping the CpG sites. Transfections of the promoter regions cloned into a reporter vector and analysis of luciferase activity showed that all promoters are functional. Co-transfections of the promoter regions with HOXD13 or DLX5 suggest that both transcription factors are positive regulators of *ZNF687* transcription though the overlapping CpG binding sites in promoter regions upstream exon 1A and 1B. We are currently evaluating if the methylation status of CpG sites affects the transcription of *ZNF687* by the identified transcription factors.

**Acknowledgements**: This work received national funds from the Portuguese Foundation for Science and Technology (FCT) through the project UIDB/04326/2020 (CCMAR). D Varela is recipient of PhD fellowship from FCT, ref: SFRH/BD/141918/2018.

**P12 –** CYTOTOXICITY ASSESSMENT OF ENDODONTIC SEALERS: METABOLIC ACTIVITY, MORPHOLOGY AND CHROMOSOMAL ALTERATIONS

Inês Moura Tavares^1^; Ilda P. Ribeiro^2^; Cláudia Pais^3^; Mafalda Laranjo^4^; Ana Jardim^3^; Alexandra Mascarenhas^3^; Mónica Zuzarte^5^; Miguel Cardoso^6^; Henrique Girão^5^; Maria Filomena Botelho^4^; Joana B. de Melo^2^; Isabel M. Carreira^2^; Rita Noites^6^

^1^Cytogenetics and Genomics Laboratory, Institute of Cellular and Molecular Biology, FMUC, Portugal; ^2^Cytogenetics and Genomics Laboratory, Institute of Cellular and Molecular Biology, FMUC, Portugal; iCBR- CIMAGO, FMUC, Portugal; Center for Innovative Biomedicine and Biotechnology (CIBB), UC, Portugal; Clinical Academic Center of Coimbra (CACC), Coimbra, Portugal; ^3^Cytogenetics and Genomics Laboratory, FMUC, Portugal; University of Coimbra, Coimbra Institute for Clinical and Biomedical Research (iCBR) and Center of Investigation on Environment Genetics and Oncobiology (CIMAGO), Faculty of Medicine, Coimbra, Portugal; ^4^iCBR-CIMAGO, FMUC, Portugal; Center for Innovative Biomedicine and Biotechnology (CIBB), UC, Portugal; Clinical Academic Center of Coimbra (CACC), Coimbra, Portugal; Institute of Biophysics, Faculty of Medicine, Coimbra, Portugal; ^5^Center for Innovative Biomedicine and Biotechnology (CIBB), UC, Portugal; Clinical Academic Center of Coimbra (CACC), Coimbra, Portugal; Coimbra Institute for Clinical and Biomedical Research (iCBR) FMUC, Portugal; ^6^Faculty Dental Medicine - Universidade Católica Portuguesa, Center for Interdisciplinary Research in Health (CIIS)

**Introduction**: Endodontic treatment aims to eliminate infection of the root canals and fill the dental pulp space, being, the obturation of root canals an important step. The study of the toxicity/biocompatibility of the sealers used to fill the root canals is crucial since they are applied into direct contact with periradicular tissues.There are several types of sealers, categorized according to their main chemical constituents. The aim of this study was to evaluate the cytotoxicity of three root canal sealers, AH Plus, Bio MTA+ and Bio C, on immortalized human gingival fibroblasts.

**Methods:** To study the cytotoxicity of the sealers we performed a Methyltetrazolium (MTT) assay, a study of cell's morphology and a cytogenetic study. Cells were placed in contact with material-conditioned media, for 24 h, at three different concentrations (1, 10 and 100 mg/ml) for the MTT assay. Cell morphology and cytogenetic studies were performed at 100 mg/ml. Cells in normal culture medium were analyzed as control group.

**Results**: MTT assay revealed a cytotoxic effect of Bio MTA+ and Bio C with a growing decrease of metabolic activity with increasing compound concentration, reaching 50% with 100 mg/ml. Regarding the cells morphology, Bio C was the compound that showed a more drastic effect, with a decrease in cell confluence and several morphological changes. AH Plus and Bio MTA+ did not seem to affect the cell confluence, however morphology changes were observed, as compromised cell membranes and loss of cell content. Cytogenetic study was thus far only performed with AH Plus. Since there was a severe decrease of mitotic index after treatment, it was not yet possible to obtain sufficient metaphases, even after several cytogenetic harvesting procedures, but, so far, no relevant structural or numerical changes were observed.

**Discussion**: This preliminary study allowed us to verify that these root canal sealers exhibit some cytotoxicity, depending on the concentration used. Although more studies are still needed, this work could be important to both, help in the selection of the most appropriate compounds for clinical practice and to determine the maximum recommended amounts of each sealer.

**P13 –** EPIGENETIC AND TRANSCRIPTIONAL ANALYSIS OF *TSC2* IN TUBEROUS SCLEROSIS PATIENTS

Sara Vasconcelos^1^; Liliana Capela^2^; C. Joana Marques^1^; Susana Fernandes^1^; Ana Grangeia^3^; Miguel Leão^3^

^1^Genetics Unit, Department of Pathology, Faculty of Medicine, University of Porto, Portugal; i3S – Instituto de Investigação e Inovação em Saúde, University of Porto, Portugal; ^2^Genetics Unit, Department of Pathology, Faculty of Medicine, University of Porto, Portugal; ^3^Genetics Unit, Department of Pathology, Faculty of Medicine, University of Porto, Portugal; Department of Medical Genetics, São João Hospital University Centre, - CHUSJ, Porto

**Introduction**: Tuberous sclerosis (TSC) is an autosomal dominant disorder presenting *TSC1* or *TSC2* gene variants that result in increased cellular proliferation. It is characterized by the formation of benign tumors in multiple organs but also neurological manifestations such epilepsy, autism spectrum disorder and intellectual disability. TSC has a variable penetrance and result in heterogeneous clinical manifestations.1 DNA methylation regulates gene expression and can represent epimutations that are often ignored and can contribute to the clinical heterogeneity. Hence, we here analysed transcriptional levels and DNA methylation of *TSC2* in blood cells from TSC patients carrying *TSC2* pathogenic variants.

**Methodology:** We analysed peripheral blood from 10 control individuals and 14 *TSC2* paediatric patients (10 from the Tuberous Sclerosis Alliance Biosample Repository and 4 from Hospital São João), carrying different *TSC2* pathogenic variants. Transcript levels of *TSC2* were measured by quantitative RT-PCR and methylation levels of 58 CpGs at *TSC2* gene promoter were studied by bisulfite sequencing.

**Results**: We observed decreased expression of *TSC2* in blood cells from TSC patients, comparing to controls. However, no changes in methylation levels at the gene promoter were observed, with this region presenting an hypomethylated state in both controls and TSC patients.

**Discussion**: Our results suggest that methylation at *TSC2* promoter is not likely to contribute to the variability of clinical manifestations observed in TSC patients; however, it would be important to analyse the most affected cells, like brain cells where *TSC2* is highly expressed, and also other gene regulatory regions in order to better understand the pathophysiology of this genetic disease.

**References**: 1. Volpi A, *et al*. *J Nephrol*. 2019; 32(3):355-363

**Acknowledgments**: This work was funded by the Tuberous Sclerosis Alliance (TSA, USA). SV and CJM are funded by FCT (SFRH/BD/147440/2019 and CEEC/IND/00371/2017, respectively)

**P16 –** FUNCTIONAL CHARACTERISATION OF RARE *LDLR* VARIANTS – QUANTITATIVE HIGH- THROUGHPUT MICROSCOPY

Rafael Graça^1^; Magdalena Zimon^2^; Ana Catarina Alves^1^; Rainer Pepperkok^2^; Mafalda Bourbon^1^

^1^Unidade de I&D, Grupo de Investigação Cardiovascular, Departamento de Promoção da Saúde e Prevenção de Doenças Não Transmissíveis, Instituto Nacional de Saúde Doutor Ricardo Jorge, Lisbon, Portugal; ^2^Cell Biology and Cell Biophysics Unit, EMBL Heidelberg, Heidelberg, Germany.

**Aim:** Mutations in the Low Density Lipoprotein receptor (*LDLR*) gene are the major cause of familial hypercholesterolaemia (FH), with over 2300 variants described in clinical FH patients. However, less than 15% of these variants have functional evidence to prove their pathogenicity. The aim of the present work is to stablish a quantitative high-throughput *in vitro* microscopy approach to functional characterise rare *LDLR* variants.

**Methods:** Wild type or mutant *LDLR* variants were overexpressed in *LDLR*-deficient CHO-ldlA7 cells. LDLR expression at cell surface and functional activity were quantified by multiparametric analysis of images acquired by high-content automated microscopy. A total of 40 variants were studied, 20 previously characterised (controls) used for assay validation, and 20 rare missense variants identified in Portuguese patients, with a clinical FH diagnose. The latter were classified as variants of unknown significance (VUS) according to the ACMG/AMP guidelines.

**Results:** Analysis of control variants confirmed the effectiveness of this approach to correctly classify *LDLR* variants according to their pathogenicity. Moreover, this work allowed to identify 13 functionally abnormal missense variants and 7 functionally normal missense variants that do not affect LDLR activity among studied VUS. Moreover, this procedure is performed in 1/3 of the time needed with the reference method for functional characterisation (flow cytometry).

**Conclusions:** Distinguish disruptive rare variants from silent rare variants is a fundamental challenge of contemporary genetics. We have established a time and cost-effective high-throughput assay to functionally profile *LDLR* variants, that can be scaled-up to higher number of variants. This strategy allows to firmly discriminate the biological effects and likely disease relevance of rare *LDLR* missense variants, contributing to an improved variant classification, and consequently to a better diagnosis and patients’ prognosis.

**P17 –** INDUCED PLURIPOTENT STEM CELLS DERIVED CARDIOMYOCYTES FROM A PATIENT WITH FABRY DISEASE: A WORK IN PROCESS CELL MODEL

Ana Joana Duarte^1^; Diogo Ribeiro^1^; José Bragança^2^; Olga Amaral^1^

^1^Instituto Nacional de Saúde Ricardo Jorge; ^2^University of Algarve

Fabry disease (FD) is one of the commonest Lysosomal Storage Disorders (LSDs) and is caused by mutations in the alpha-galactosidase A gene (GLA) from which results a deficient activity of the lysosomal hydrolase alpha- galactosidase A (α-Gal A). This deficiency leads to progressive multisystemic accumulation of glycolipids, namely, globotriaosylceramide (Gb3) and globotriaosylsphingosine (lyso-Gb3), in plasma and in a wide range of cells, particularly in the relevant cells affected by the disease like vascular endothelial cells, podocytes, cardiomyocytes, and arterial smooth muscle cells. Taking FD as a cardiac disorder, our aim was to differentiate induced pluripotent stem cells (iPSCs) reprogrammed from FD patients’ fibroblasts into cardiomyocytes, a cellular type usually targeted by this disease. For this purpose, firstly we reprogram FD patients’ cells and a normal control cell line using the non-integrative Sendai Virus method. After achieving the iPSCs state, the cells were submitted to differentiation using specific cardiomyocytes differentiation effectors. After 14 days in culture, we obtained functional iPSC-cardiomyocytes (iPSC-CMs) that express relevant physiological markers, present contractility, and can be subsequently maintained in culture using the appropriate maintenance medium. The resulting iPSC-derived cells will be analyzed against the initial FD fibroblast to see if the disease features are replicable in the new cell lines. Although our results are promising, we still have to design and execute a protocol that allow our iPSC-CMs to achieve the maturation state. Since only mature cardiomyocytes can recapitulate the same disease phenotypes present in vivo and serve as an accurate disease model, we have to surpass the immature state to achieve a complete disease model.

This work was financed by FCT (PTDC/BIM-MEC/4762/2014) and carried out at INSA-DGH.

**P18 –** MITIGATING THE PROGEROID PHENOTYPE: A GENE DELIVERY STRATEGY TO IMPROVE PROGERIN-ASSOCIATED ABNORMALITIES

Rafael G. Costa^1^; Célia Aveleira^2^; Clévio Nóbrega^1^

^1^Centre for Biomedical Research (CBMR), Universidade do Algarve; ^2^Center for Neuroscience and Cell Biology (CNC), Universidade de Coimbra.

**Introduction:** Lamins compose the nuclear lamina and confer integrity to the nucleus. Mutations in different lamins’ genes are associated with several diseases [1], including Hutchinson-Gilford Progeria syndrome (HGPS). HGPS is an incurable rare disease characterized by aging acceleration towards a premature death. A mutation in the lamin A/C-encoding gene (*LMNA*) produces a truncated protein, termed progerin, which accumulates in the nuclear periphery [2]. Consequently, HGPS cells exhibit age- associated markers, such as enhanced DNA damage [3], and senescence [4]. Previous work in our group showed that a specific RNA-binding protein (RBPX) modulates the levels of different transcripts. Due to HGPS molecular pathogenesis, we hypothesized that RBPX could interfere with progerin processing and thus attenuate the age- associated markers.

**Methodology:** HEK293T cells were transfected with progerin or co-transfected with progerin and RBPX. After

48 hours, the cells were harvested for fluorescence microscopy and immunoblot analyses to assess the nuclear circularity index and progerin levels, respectively. Furthermore, we transduced human HGPS fibroblasts with lentiviral vectors encoding RBPX where the DNA damage was assessed by the phospho-Histone H2A.X.

**Results:** RBPX overexpression in progerin-expressing cells showed (i) an increase in the nuclear circularity index, (ii) a decrease in progerin protein levels, and (iii) a reduction in DNA damage.

**Discussion:** The ability of RBPX in reducing progerin levels makes it a promising therapeutic target for HGPS. In this light, we suggest RBPX gene delivery as an innovative therapeutic approach to mitigate the HGPS phenotype. Nevertheless, further research is needed to assess safety and efficacy *in vivo* models.

**References:** [1] Worman et al., 2020, Cold Spring Harb Perspect Biol, 2:a000760. [2] Goldman *et al.*, 2004, 101:8963. [3] Gonzalo and Kreienkamp, 2015, Current opinion in cell biology, 34:75. [4] Benson et al., 2010, J Cell Sci, 123:2605.

**Acknowledgements:** CN laboratory is supported by the AFM-Téléthon, the Ataxia UK and the Fundação para a Ciência e Tecnologia.

Declaration of Interest: The authors declare no conflict of interest.

**P19 –** AN RNA BINDING PROTEIN ALLEVIATES PATHOLOGY IN TWO DIFFERENT MOUSE MODELS OF POLYGLUTAMINE DISEASES

André Conceição^1^; Rebekah Koppenol^2^; Sandra Tomé^3^; Sara Carmo-Silva^3^; Carlos A. Matos^2^; Luís Pereira de Almeida^4^; Clévio Nóbrega^2^

^1^Centre for Biomedical Research, Universidade do Algarve, Faro, Portugal; ^2^Centre for Biomedical Research, Universidade do Algarve, Faro, Portugal; Department of Biomedical Sciences and Medicine, Universidade do Algarve, Faro, Portugal; Center for Neuroscience and Cell Biology, Universidade de Coimbra, Coimbra, Portugal; ^3^Center for Neuroscience and Cell Biology, Universidade de Coimbra, Coimbra, Portugal; ^4^Center for Neuroscience and Cell Biology, Universidade de Coimbra, Coimbra, Portugal; Faculty of Pharmacy, University of Coimbra, Coimbra, Portugal.

**Introduction:** Polyglutamine (PolyQ) diseases are incurable neurodegenerative disorders characterized by an abnormal CAG expansion in the respective disease associated gene [1]. The mutant gene product are proteins bearing an overexpanded PolyQ tract. Affected patients display progressive motor symptoms that ultimately culminate in premature death [2]. Spinocerebellar ataxia type 2 (SCA2) and type 3 (SCA3) are PolyQ diseases in which the Ataxin-2 and Ataxin-3 proteins, respectively, contain an abnormal polyQ tract prone to aggregate [3]. Previously, our group showed that a specific RNA binding protein (RBPX) modulates mRNA dynamics, regulating their translation. Thus, we hypothesised that RBPX could regulate Ataxin-2 and Ataxin-3 expression alleviating disease associated phenotypes.

**Methods:** N2a cell were co-transfected with mutant Ataxin-2 and RBPX or mutant Ataxin-3 and RBPX. Ataxin- 2 and Ataxin-3 expression levels and protein aggregation were assessed by western-blot and fluorescence microscopy, respectively.

Lentiviral mouse models of SCA2 and SCA3 were injected into the striatum with lentiviral particles encoding for RBPX protein. Ataxin-2 and Ataxin-3 aggregation and neurodegeneration were assessed by microscopy.

**Results:** RBPX reduces expression levels and aggregation of mutant Ataxin-2 and Ataxin-3 *in vitro* and *in vivo*. Mouse models of SCA2 and SCA3 showed reduced neurodegeneration when injected with lentiviral particles encoding for RBPX protein. RBPX protein also displayed a positive safety profile.

**Discussion:** RBPX overexpression in two different PolyQ mouse models, SCA2 and SCA3, reduced Ataxin-2 and Ataxin-3 aggregation and safely rescued disease associated neurodegeneration. Therefore, we propose RBPX gene delivery as novel and safe therapy to treat different PolyQ diseases.

**Acknowledgements:** This work was supported by the AFM-Téléthon, the Ataxia UK, and the Fundação para a Ciência e Tecnologia.

**References:** [1] Matos et al. 2018 Adv Exp Med Biol; [2] Paulson et al. 2017 Nat Rev; [3] Nóbrega et al. Brain 2015.

Declaration of interests: The authors declare no conflict of interest.

**P20 –** IDENTIFICATION OF mTOR AND AGO1 IRES TRANS-ACTING FACTORS

Rita R. Marques^1^; Rafaela Lacerda^1^; Luísa Romão^1^

^1^Department of Human Genetics, Instituto Nacional de Saúde Doutor Ricardo Jorge, Av. Padre Cruz, 1649-016 Lisbon, Portugal; Biosystems and Integrative Sciences Institute (BioISI), Faculdade de Ciências, Universidade de Lisboa, Lisbon, Portugal

Cancer is the second leading cause of death globally; therefore, its study is crucial to discover new therapies. Under stress, the regular process of protein synthesis (canonical translation) is impaired, while a back-up mechanism mediated by internal ribosome entry sites (IRES) continues to function, allowing the synthesis of proteins that maintain cellular viability. This also happens in cancer cells, contributing for their survival and consequent tumorigenesis. IRES-mediated translation and its regulation by IRES *trans*-acting factors (ITAFs) has been correlated to metastasis and chemotherapeutic drug resistance. Therefore, our main goal was to validate ITAFs and assess their significance in cancer onset, thus becoming candidates as novel therapeutic targets. A bicistronic reporter system containing a first cistron translated via canonical translation and a second one translated by mTOR1 or AGO12 IRES was used to test IRES-driven translation initiation activity. Experiments were carried out in which several proteins (hnRNPs) were silenced by specific siRNAs to analyse their function as ITAFs of mTOR and AGO1 IRESs. Also, distinct drugs were applied to simulate endoplasmic reticulum (ER) or hypoxia stress, to evaluate their effect on IRES activity. The relative IRES activity was assessed by luminescence tests and the protein levels by Western blot. In general, knockdown of hnRNPK and hnRNPU seems to decrease the IRES activity by ∼60% and ∼30% respectively, while hnRNPC knockdown does not show a significant effect. Regarding the ER stress, hnRNPK knockdown seems to decrease even more the IRES activity, while hnRNPU depletion induces a significant increase. On the other hand, under hypoxia, the hnRNPs knockdowns do not significantly affect IRES activity. These results indicate that hnRNPK and hnRNPU may function as ITAFs of mTOR and AGO1 IRES activity in cells under ER stress. Our data can be decisive for a better understanding of carcinogenesis and suggest new therapeutic targets for cancer treatment.

1. Marques-Ramos, A., et.al. 2017. RNA. 23, 1712-1728

2. Lacerda, R. 2016. Faculdade de Ciências e Tecnologia da Universidade NOVA de Lisboa.

**P21 –** REGULATION OF IRES-MEDIATED TRANSLATION IN *P53*

Inês Fonseca Costa^1^; Rafaela Lacerda^2^; Maria López-Iniesta^3^; Luísa Romão^4^; Marco M. Candeias^3^

^1^MaRCU — Molecular and RNA Cancer Unit; Departamento de Genética Humana, Instituto Nacional de Saúde Dr. Ricardo Jorge, Lisboa, Portugal; ^2^MaRCU — Molecular and RNA Cancer Unit; Departamento de Genética Humana, Instituto Nacional de Saúde Dr. Ricardo Jorge, Lisboa, Portugal; BioISI — BioSystems & Integrative Sciences, Faculdade de Ciências, Universidade de Lisboa, Lisboa, Portugal; ^3^MaRCU — Molecular and RNA Cancer Unit; Graduate School of Medicine, Kyoto University, Kyoto, Japan; ^4^Departamento de Genética Humana, Instituto Nacional de Saúde Dr. Ricardo Jorge, Lisboa, Portugal; BioISI — BioSystems & Integrative Sciences, Faculdade de Ciências, Universidade de Lisboa, Lisboa, Portugal.

The tumor microenvironment is characterized by several stresses impairing canonical translation. However, specific mRNAs harboring internal ribosome entry sites (IRES), such as several tumour suppressors and oncogenes, can overcome this impairment. The tumor suppressor *TP53* gene, an important transcription factor that ensures cellular homeostasis, is frequently mutated in human cancers. Over the years, several p53 isoforms have been identified, which in some cases result from alternative initiation of translation regulated by an IRES. Recently, we have associated mutant p53 “gain-of-function” cancer phenotype, such as enhanced cell survival, invasion, proliferation, and adhesion, with the expression of higher levels of shorter p53 isoforms, such as Δ160p53 isoform.^1^ Here, we used a bicistronic system containing two reporter luciferases (renilla luciferase and firefly luciferase) to assess IRES-mediated translation. Several *p53* mRNA elements were tested in this system and, interestingly, we have found an inhibitory element of IRES-mediated translation. Overall, IRES-mediated translation in malignant cells is used to translate specific proteins that promote cancer progression. Thus, inhibiting translation of oncogenes via IRES could prevent the formation of tumor cells and their adaptation to unfavourable conditions in the tumor microenvironment.

1. Candeias, M. M., Hagiwara, M. & Matsuda, M. Cancer-specific mutations in p53 induce the translation of Δ160p53 promoting tumorigenesis. EMBO Rep. 17, 1542–1551 (2016).

**P22 –** IDENTIFICATION OF NEUROTRANSMISSION AND SYNAPTIC RISK GENES AND DISRUPTED BIOLOGICAL PROCESSES IN AUTISM SPECTRUM DISORDER

Joana Vilela^1^; Hugo Martiniano^1^; Ana R Marques^1^; João Santos^1^; Célia Rasga^1^; Guiomar Oliveira^2^; Astrid Vicente^1^

^1^Departamento de Promoção da Saúde e Doenças não Transmissíveis, Instituto Nacional de Saúde Doutor Ricardo Jorge, Lisboa, Portugal; ^2^Unidade de Neurodesenvolvimento e Autismo (UNDA), Serviço do Centro de Desenvolvimento da Criança, Centro de Investigação e Formação Clínica, Hospital Pediátrico, Centro Hospitalar e Universitário de Coimbra, Coimbra, Portugal.

**Introduction:** Autism Spectrum Disorder (ASD) is a neurodevelopmental disorder characterized by communication deficits and repetitive behaviors, with a strong genetic component. However, the genetics underlying the disease is unclear. Objective: To identify neurotransmission and synaptic (NS) genes with a role in ASD as there is evidence that the disruption of these mechanisms is implicated in the disease etiology.

**Methods:** We defined 1216 NS candidate genes by filtering for ‘neurotransmitter’ and ‘synapse’ in Gene Ontology, Reactome and KEGG; we overlapped the gene list obtained with the databases SynaptomeDB and SynSysNet, and with a gene list obtained from a literature review. We searched for ultra-rare high-confidence loss of function (LoF) Single Nucleotide Variants (MAF < 0, 1%) in these genes in the Whole Exome Sequencing dataset from the Autism Sequencing Consortium (N = 3938 cases) and in gnomAD (N = 60146 controls). Genes with variants of interest were used to construct a network of protein-protein interactions (PPI) from the STRING database. Network clusters were identified using the Leiden community detection.

**Results:** We selected 358 LoF variants in 209 genes and constructed a PPI network. We identified 59 genes as being part of a main network that interconnects different biological processes affecting several of the cases analysed. We identified 7 biological communities involved in cellular processes at different levels. Some of these genes encode molecules previously associated to ASD, such as subunits from calcium channels (CACNB4; CACNA2D1; CACNA1C; CACNA1S), or implicated in processes suspected to have a role in neuronal development, such as the immune system (IRAK2; IL1B; TRAF6; IRAK3; IRAK4).

**Discussion:** The main network genes are distributed by 7 biological processes that may underlie the genetic component of ASD, some were already proposed as source of synaptic dysregulation, such as cell adhesion molecules and axon guidance. This work reinforces the idea that basic cellular processes may compromise neuronal development and synaptic signaling such as cell signaling at the immune system level (interleukin function), MAP kinase or G alpha signaling.

**P23 –** AUTOPHAGY IMPAIRMENT IN SPINOCEREBELLAR ATAXIA TYPE 2: FIRST EVIDENCES AND A POTENTIAL THERAPEUTIC STRATEGY

Adriana Marcelo^1^; Ana Rosa^2^; Marta Santos^3^; Benedita Ferreira^3^; Rebekah Koppenol^1^; Carlos A. Matos^4^; Clévio Nóbrega^4^

^1^Center for Biomedical Research, University of Algarve, Faro, Portugal; Center for Neuroscience and Cell Biology, University of Coimbra, Coimbra, Portugal; PhD Program in Biomedical Sciences, Department of Biomedical Sciences and Medicine, University of Al; ^2^Faculty of Sciences and Technology, University of Algarve; ^3^Center for Biomedical Research, University of Algarve, Faro, Portugal; ^4^Center for Biomedical Research, University of Algarve, Faro, Portugal; Center for Neuroscience and Cell Biology, University of Coimbra, Coimbra, Portugal; Department of Biomedical Sciences and Medicine, University of Algarve, Faro, Portugal; Algarve Biome

**Introduction:** Spinocerebellar ataxia type 2 (SCA2) is a genetic neurodegenerative disorder, which belongs to the group of polyglutamine diseases, caused by an expansion of CAG trinucleotide repeats in the disease-causing genes. It is widely accepted that these disorders share similar mechanisms of pathogenesis, including protein aggregation and proteolytic cleavage [1]. Autophagy impairment has also been reported in several polyglutamine diseases, however there are no studies concerning the role of autophagy in SCA2 [2]. Therefore, in this work we aimed at evaluating the autophagic pathway in different models of SCA2. Moreover, we tested whether the molecular induction of autophagy could rescue the phenotype observed in models of this disorder.

**Methodology:** To evaluate the autophagic process, we used mouse *in vitro* and *in vivo* models of SCA2, as well as patients’ brain samples. We assessed the expression levels of several autophagic markers through western blot, immunohistochemistry and qRT-PCR. We also induced the autophagic process in the mouse models using Cordycepin and analyzed its potential therapeutic effect [3].

**Results:** Regarding the results obtained in the mouse models of SCA2, we observed an increase in protein and mRNA levels of SQSTM1 and a decrease in LC3B protein levels. Additionally, we observed the presence of SQSTM1 and LC3B inclusions in patients’ brain tissue. Moreover, molecular induction of autophagy led to a decrease in mutant protein levels.

**Discussion:** The results obtained revealed the first evidences of autophagy dysfunction in SCA2, as altered levels and accumulation of autophagic markers were observed in different models. Moreover, we observed that molecular activation of the autophagic pathway results in mitigation of neuropathological alterations in SCA2.

**References:** [1] Matos CA et. Al. *Adv Exp Med Biol.* 2018; 1049:395-438; [2] Cortes CJ, La Spada AR. *Mol Cell Neurosci.* 2015; 66(A):53-61; [3] Marcelo A et. Al. Hum Mol Genet. 2019; 28(1):51-63.

**Acknowledgments**: This work was supported by AFM-Téléthon, Ataxia UK and Fundação para a Ciência e Tecnologia.

Declaration of Interests: The authors declare no conflict of interest.

**CLINICAL RESEARCH**

**P25 –***SCRIB* AND *PIK3CB* AS A PREDICTOR OF WORSE PROGNOSIS IN ORAL SQUAMOUS CELL CARCINOMAS

Ilda P. Ribeiro^1,2,3,4+^, Luísa Esteves^1+^, Leonor Barroso^5^, Francisco Marques^2,4,6,7^, Francisco Caramelo^2,8^, Joana B. Melo^1,2,3,4^, Isabel M. Carreira^1,2,3,4^

^1^Cytogenetics and Genomics Laboratory, Institute of Cellular and Molecular Biology, Faculty of Medicine, University of Coimbra, Coimbra, Portugal; ^2^University of Coimbra, Coimbra Institute for Clinical and Biomedical Research (iCBR) and Center of Investigation on Environment Genetics and Oncobiology (CIMAGO), Faculty of Medicine, Coimbra, Portugal; ^3^University of Coimbra, Center for Innovative Biomedicine and Biotechnology (CIBB), Coimbra, Portugal; ^4^Clinical Academic Center of Coimbra (CACC), Coimbra, Portugal. ^5^Maxillofacial Surgery Department, Coimbra Hospital and University Centre, CHUC, EPE, Coimbra, Portugal ^6^Department of Dentistry, Faculty of Medicine, University of Coimbra, Coimbra, Portugal; ^7^Stomatology Unit, Coimbra Hospital and University Centre, CHUC, EPE, Coimbra, Portugal; ^8^Laboratory of Biostatistics and Medical Informatics, iCBR - Faculty of Medicine, University of Coimbra, Coimbra, Portugal; ^+^Both authors contributed equally to this work.

**Introduction:** Oral squamous cell carcinoma (OSCC) is an aggressive and lethal disease. This study aimed to perform a genome-wide characterization of OSCC patients and to identify the most common altered chromosomes, signalling pathways and genes and consequently to establish potential prognosis biomarkers.

**Methodology:** A whole genome characterization of tumor samples from 62 OSCC patients was performed using array comparative genomic hybridization. The identification of a genomic signature and prognosis biomarkers was carried out by applying several statistical methods.

**Results:** A total of 8507 chromosomal regions with copy number alteration were detected. The most frequently amplified chromosomes were 3q, 8q, 11q, 15q and 17q. The most frequently deleted chromosomes were 3p, 5q, 6p, 7q, 8p, 11q and 18q. The GO terms associated with the most frequently amplified (n = 14302) and deleted genes (n = 880) were determined, resulting in a set of 748 terms associated with the amplifications and 407 associated with the deletions (p < 0.01). Taking into account a set of terms closely associated with the carcinogenic process, further comparisons were drawn between these two sets of genes. This step resulted in 60 terms associated with the amplified genes and 7 terms associated with the deleted genes (p < 0.01). From the initial 21939 mapped genes in the 8507 regions, 472 genes are left after a 35% frequency filter. A signalling pathway analysis was performed, resulting in 15 signalling pathways that are overrepresented (p < 0.05). A total of 49 genes that were associated with those pathways were considered for further analyses. Multivariate analyses and logistic regression models identified both *SCRIB* (OR = 0.11, *p* = 0.015) and *PIK3CB* (OR = 0.041, *p* = 0.04) with a significant predictive value associated with the development of metastasis/relapse.

**Conclusion:** This study gave a step forward in the identification of prognosis biomarkers with great potential for patients’ management.

**P26 –** MULTIGENE STUDIES IN THE MOLECULAR DIAGNOSIS OF HUMAN DISORDERS OF SEXUAL DEVELOPMENT BY NEXT GENERATION SEQUENCING

Beatriz Oliveira^1^; Iris Pereira Caetano^1^; Pedro Rodrigues^1^; Rita S. Silva^2^; Ana Grangeia^3^; André Travessa^4^; Joana Mendonça^1^; Luís Vieira^5^; Francisco Pina Martins^6^; João Gonçalves^5^

^1^Human Genetics Department, National Institute of Health Dr Ricardo Jorge, Lisbon, Portugal; ^2^Paediatrics Service of Hospital de São João, Porto, Portugal; ^3^Medical Genetics Service of Hospital São João, Porto, Portugal; ^4^Medical Genetics Service of Hospital St^a^ Maria, Lisbon, Portugal; ^5^Human Genetics Department, National Institute of Health Dr Ricardo Jorge, Lisbon, Portugal; Center for Toxicogenomics and Human Health, Nova Medical School, Lisbon, Portugal; ^6^Department of Animal Biology, Faculty of Sciences, University of Lisbon, Lisbon, Portugal

**Introduction:** Disorders of sexual development (DSD) are congenital conditions in which the chromosomal, gonadal, or anatomical sex is atypical. Molecular studies of some genes, using PCR and *Sanger* sequencing (PCR&S), usually don’t detect pathogenic alterations in most patients with a clinical diagnosis of DSD. In such cases, affected individuals can remain, sometimes for many years, without a definitive diagnosis, appropriate clinical management, and treatment. The aim of this study was to validate the Next Generation Sequencing (NGS) analysis for a multigene panel designed for DSD in order to provide a faster molecular diagnosis with lower costs and contribute to a more effective diagnosis.

**Methodology:** A panel with 40 genes and 35 anonymized DNA samples with 90 known variants (SNVs and SNPs), was used for validation by NGS. In addition, 15 DNA samples from patients with DSD were analysed, after informed consent had been obtained. Some of these were analysed for a few genes, by PCR&S, and no molecular defects were identified. NGS included *Ampliseq*, *MiSeq* platform, and V*ariant Interpreter (*all *from* Illumina), and other bioinformatics tools (IGV, VEP, HSF, and VarSome).

**Results/discussion:** Through this approach, 86 variants were validated in 18 genes (validation rate: 95.6%). In the 15 samples where the classical methodology (PCR&S) was not able to find the molecular defect, NGS detected pathogenic variants in 2 of them (13%). In a first patient, two alterations (both in heterozygosity) were identified in *HSD17B3* (c.876_877dupAA; c.645A > T), which can explain the patient's phenotype. In a second patient a homozygous variant, c.277+4A > T, was also found in *HSD17B3*. These two 46,XY females, had a clinical suspicion of androgen insensitivity syndrome, although both were negative for variants in the *AR* gene. This study allowed us to validate the NGS approach for a wide number of genes and contributed to an increased detection rate of pathogenic variants in patients with DSD improving their clinical diagnosis.

**Acknowledgments:** Research partially supported by FCT/MCTES, Toxicogenomics, and Human Health (UIDB/00009/2020). GenomePT project (POCI-01-0145-FEDER-022184).

**P27 –** MOLECULAR CHARACTERIZATION OF A COHORT OF PATIENT'S WITH CADASIL

Inês Elias^1^; Ana Santos^1^; Gustavo Santo^2^; Maria R. Almeida^1^

^1^CNC - Center for Neuroscience and Cell Biology; ^2^Neurology Department, Coimbra University Hospital

Cerebral autosomal dominant arteriopathy with subcortical infarcts and leukoencephalopathy (CADASIL, OMIM #125310) is the most common inherited cerebral small vessel disease, with a recent new estimated prevalence of 1:300. It is caused by mutations in the *NOTCH3* gene which vary in their frequency across populations. More than 10 years ago, studies showed exons 4, 11, 18, and 19 to be the most frequently mutated in CADASIL Portuguese patients. However, since then, no further studies have been done in our CADASIL population. Therefore, our aim was to detail *NOTCH3* gene mutational spectrum in a cohort of CADASIL patients from the Center Region of Portugal. CADASIL patients were clinically evaluated at the vascular outpatient unit of the Neurology department of the Centro Hospitalar e Universitário de Coimbra (CHUC) and were referred to the Neurogenetics laboratory at the Center for Neuroscience and Cell Biology (CNC) for their molecular analysis. The genetic studied was performed by Sanger and/or NGS sequencing and the identified variants were further evaluated using an in-house bioinformatic pipeline. A total of 53 *NOTCH3* mutation carriers were identified. Of those, 20 were index cases from different families and 33 were relatives. The molecular analysis revealed 12 different heterozygous mutations in which eight were missense cysteine altering mutations (p.R110C, p.R153C, p.G420C, p.R427C, p.C446F, p.R558C, p.R607C, p.C1099Y), three were missense cysteine sparing mutations (p.S497L, p.S978R, p.V1952 M) and one was a novel nonsense mutation (p.R1893∗). Of note, all the missense mutations were previously reported in the literature. Interestingly, the majority of the *NOTCH3* gene mutations were located in exon 8, 11, 20 or 31 (18.9%, 47.2%, 11.3% and 9.4% respectively), while the remaining ones were dispersed over the entire *NOTCH3* coding sequence. Accordingly, our results suggest that sequencing of the entire coding region of the *NOTCH3* geneis relevant for CADASIL mutation analysis in the Portuguese population. Additionally, our study enlarged the pathogenic *NOTCH3* mutation spectrum, with a novel nonsense mutation identified.

**P28 –** HAPLOTYPE DISTRIBUTION AND GENOTYPIC DIVERSITY AMONG ANGOLAN CHILDREN WITH SICKLE CELL DISEASE

Mariana Delgadinho^1^; Brígida Santos^2^; Miguel Brito^1^

^1^H&TRC-Health & Technology Research Center, ESTeSL- Escola Superior de Tecnologia da Saúde, Instituto Politécnico de Lisboa; ^2^Hospital Pediátrico David Bernardino (HPDB) e Centro de Investigação em Saúde de Angola (CISA), Angola.

Sickle cell disease (SCD) is an inherited blood disorder that affects over 300,000 newborns worldwide every year. Despite being a monogenic disease, SCD shows a remarkably high clinical heterogeneity and analysis of the HBB gene cluster has revealed five distinct haplotypes: Senegal (SEN), Benin (BEN), Central African Republic (CAR), Cameroon (CAM) and Arab-Indian (ARAB). The aim of this study was to assess the frequency of HBB haplotypes, as well as to correlate other genetic predictors that could have an impact on SCD phenotype in an Angolan pediatric population.

This study analyzed clinical and biological data collected from 192 Angolan children. DNA was extracted from peripheral blood samples and paired-end sequencing was performed using the NextSeq 550. Haplotype classification was based on four previously described SNPs (rs3834466, rs28440105, rs10128556, and rs968857) and the genotype for the SNPs in *HBG2* (rs7482144*)*, *BCL11A* (rs4671393) and HBS1L-MYB (rs28384513, rs4895441) genes were also obtained.

The most prevalent haplotype was the CAR/CAR, detected in 91.7%, followed by the CAR/BEN in 5.7%. Surprisingly, all the patients had at least one CAR allele and an ANOVA test showed significant differences in fetal hemoglobin between all the haplotypes and in the patients with rs7482144 and rs4671393. Patients with rs4671393 also had considerable differences in the hemoglobin and neutrophils count. Considering rs28384513 polymorphism, a significant difference was observed in the neutrophils count and the reticulocytes count was statistically significant for rs4895441.

This study provides a relevant contribution to the Angolan's population genetic background, where CAR haplotype is undoubtedly the commonest HBB haplotype and significant differences were observed in several hematological parameters. We believe that the use of NGS approaches could expand our knowledge of SCD heterogeneity and related severity, since it allows the study of multiple variants. These results emphasize the importance of personalized health care for SCD.

**Acknowledgements**: This work was supported by FCT/Aga Khan (project n°330842553) and FCT/MCTES (UIDB/05608/2020 and UIDP/05608/2020).

**P29 –** PERSONALIZED MEDICINE IN RARE DISEASES: A FOCUS ON MITOCHONDRIAL DISEASES

Nogueira C^1,2^, Pereira C^2^, Silva L^1^, Leão Teles E^3^, Rodrigues E^3^, Campos T^3^, Janeiro P^4^, Gaspar A^4^, Dupont J^4^, Bandeira A^5^, Martins E^5^, Soares G^5^, Magalhães M^5^, Sequeira S^6^, Vieira JP^6^, Santos H^7^, Vilarinho L^1,2^

^1^Unidade I&D, Departamento de Genética Humana, Instituto Nacional de Saúde Doutor Ricardo Jorge, Porto, Portugal; ^2^Unidade de Rastreio Neonatal Metabolismo e Genética, Instituto Nacional de Saúde Doutor Ricardo Jorge, Porto, Portugal; ^3^Centro Hospitalar São João, EPE, Porto, Portugal; ^4^Centro Hospitalar Lisboa Norte, EPE, Lisboa, Portugal; ^5^Centro Hospitalar e Universitário do Porto, EPE, Porto, Portugal; ^6^Centro Hospitalar Lisboa Central, EPE, Lisboa, Portugal; ^7^Centro Hospitalar de Vila Nova de Gaia, EPE, Porto, Portugal.

**Background:** Mitochondrial diseases are a group of rare inherited disorders characterized by extreme phenotypic heterogeneity that can be transmitted by any mode of inheritance, with hitherto no effective therapy options. It is estimated that 1:5,000 individuals will develop a mitochondrial disease. The molecular diagnosis in mitochondrial disorders is a great challenge and compared with traditional diagnostic testing approaches, the recent evaluations of Next Generation Sequencing (NGS) for mitochondrial disorders have shown that this methodology is more likely to provide a diagnosis, being quicker and cheaper. Many of the newly nuclear gene *loci* linked to mitochondrial disease genes have been discovered with NGS methods as well as novel phenotypes associated to genes previously linked to mitochondrial disease.

**Objectives:** The purpose of our project∗ was to develop a NGS strategy to identify the genetic defects in 250 patients suspicious of mitochondrial disorders, to confirm the clinical and biochemical diagnosis of the disease.

**Methods:** NGS was performed in a MiSeq Illumina instrument using a custom gene panel with 209 nuclear genes involved in mitochondria metabolism, and the entire human mitochondrial genome, applying SureSelect QXT target enrichment and Nextera XT, respectively.

**Results:** A molecular diagnosis was attained in 62/250 (25%) of the studied patients that harbored 21 pathogenic variants, previously reported in the literature, 11 novel variants probably pathogenic and 54 novel variants of unknown significance. These mutations were confirmed by Sanger sequencing in the index cases and in their relatives.

**Discussion and Conclusion:** Our NGS approach revealed to be an useful strategy to provide a molecular diagnosis in a substantial fraction of patients with mitochondrial diseases of unclear etiology, expanding the mutational spectrum of these disorders. Undiagnosed patients will be selected for Exome Sequencing.

∗This Research Project was supported by FCT (Fundação da Ciência e Tecnologia) (PTDC/DTP-PIC/2220/2014) and by NORTE2020 (NORTE- 01-0246-FEDER-000014 DESVENDAR “DEScobrir, VENcer as Doenças rARas”).

**P30 –** WHAT THE MICROSCOPE DO NOT SEE, ARRAY-CGH CAN DETECT - TOWARDS A MORE RELIABLE PHENOTYPIC CORRELATION

Susana Isabel Ferreira^1^; Mariana Val^1^; Luis Miguel Pires^1^; Cláudia Pais^1^; Marta Pinto^1^; Ana Jardim^1^; Isabel Marques Carreira^2^; Joana Barbosa de Melo^2^

^1^Laboratório de Citogenética e Genómica – Faculdade de Medicina da Universidade de Coimbra, Portugal; ^2^Laboratório de Citogenética e Genómica – Faculdade de Medicina da Universidade de Coimbra, Portugal; CIBB- Centro de Inovação em Biomedicina e Biotecnologia, Universidade de Coimbra, Portugal; iCBR CIMAGO – Centro Investigação em Meio Ambiente Genética e Oncobiologia

The value of array-CGH in diagnostics is unquestionable, as it has enabled the detection of submicroscopic copy number variations (CNVs) of clinical significance, increasing the yield of clinical genetic diagnoses, when compared to cytogenetic analysis. Cytogenetically, rearrangements like inversions and translocations might appear balanced at the microscopic level, but sometimes present cryptic rearrangements in the chromosomes involved in the rearrangement or others.

In 32 samples, 7 prenatal and 25 postnatal, that presented inversions (10), translocations (16) deletions (5) and one ring chromosome characterized by cytogenetics, 180K oligonucleotide aCGH was performed.

In the inversions, six had no further imbalances after aCGH, one had imbalances in the chromosome with the cytogenetically visible inversion, and three had imbalances in chromosomes different from the one involved in the inversion.

In the deletion cohort, a cytogenetically visible chromosome 6 terminal deletion prenatally detected revealed to have an extra chromosome 3 paternal deletion after aCGH, and in the four prenatal samples, one also revealed a cryptic imbalance additional to the cytogenetic visible deletion.

In the 16 translocations, the two detected at prenatal and seven out of 25 postnatal were balanced at aCGH, 4 had imbalances on the chromosomes involved in the apparently balanced rearrangements, and 4 had cryptic imbalances in chromosomes other than the translocated.

The ring chromosome 21 detected in a prenatal sample, revealed to have a paternal duplication at chromosome 17, on a syndromic region with high clinical variability.

Of the 32 samples, in 18 aCGH revealed no further imbalances and in 14 submicroscopic imbalances were detected, nine in chromosomes different from the ones involved in the rearrangements and five in the chromosomes involved in the translocation or inversion.

These results show that after an apparently balanced rearrangement is observed in conventional cytogenetic, the phenotypic impact can only be correctly established after cryptic imbalances have been disregarded by aCGH, as it is likely that other imbalances might be present, contributing to phenotype.

**P31 –** COUNSELLING SUPERVISION: ARE WE PRACTISING IN A SAFE ENVIRONMENT AT GENETIC SERVICES?

Milena Paneque^1^; Lídia Guimarães^2^; Joana Bengoa^3^; Sara Pasalodos^4^; Christophe Cordier^5^; Irene Esteban^6^; Ramona Moldovan^7^; Carolina Lemos^8^; Clara Serra-Juhé^9^

^1^i3S – Instituto de Investigação e Inovação em Saúde; IBMC–Institute for Molecular and Cell Biology; Centre for Predictive and Preventive Genetics (CGPP), Universidade do Porto, Portugal; 2AAJUDE - Associação de Apoio à Juventude Deficiente; ^3^Hôpital Necker Enfants Malades, Paris, France; ^4^Department of Medical Genetics, Complejo Hospitalario de Navarra, Universidad Pública de Navarra (UPNA), Navarrabiomed-IdiSNA (Navarra Institute for Health Research), Pamplona, Navarra, Spain; ^5^Department of Genetics, SYNLAB Genetics, Lausanne, Switzerland; ^6^Clinical Genetics Department, Queen Elizabeth University Hospital. Glasgow, Scotland; ^7^Department of Psychology, Babeş-Bolyai University, Romania; Division of Evolution and Genomic Sciences, University of Manchester, United Kingdom; Manchester Centre for Genomic Medicine, Manchester University Hospitals NHS Foundation Trust, UK; ^8^i3S – Instituto de Investigação e Inovação em Saúde; UnIGENe, IBMC – Institute for Molecular and Cell Biology; ICBAS – Instituto de Ciências Biomédicas Abel Salazar, Universidade do Porto; ^9^Servei de Genètica, Hospital de la Santa Creu i Sant Pau.

Genetic testing is becoming more commonplace in general and specialist health care, and should always be accompanied by genetic counselling. Genetic counseling has consistently been shown to impact patients and families and, understandingly, counselors too. Based on these premises counselling supervision has emerged as a service to enable professionals who provide genetic counselling consultations to acknowledge whether and how their own experiences, thoughts or feelings could impact their work with patients, to explore and manage the impact the genetic counselling process could have for their work. Clinical and counselling supervision of practitioners plays a key role in quality assurance of practice and providing a safety environment for patients and professionals.

We conducted a study that aimed to explore the current status of genetic counseling supervision provision across Europe and to ascertain factors that might be relevant for the successful implementation of counselling supervision. A total of 100 practitioners responded to the online survey; respondents were from 18 countries, with the majority working in France (27%) and Spain (17%). Only 34 participants reported having access to genetic counselling supervision. Country of origin, the existence of a regulation system and years of experience were factors identified as relevant influencing access and characteristics of counselling supervision.

With this work we intend to invite portuguese genetics healthcare professionals to reflect on the relevance of counselling supervision assurance in our national services while providing a landscape of other European services. Although there is a growing number of genetic counsellors trained at European level, just a few countries have implemented and required as mandatory the access to genetic counselling supervision, which is essential to ensure a safe and effective genetic counselling. The importance of counselling supervision for the improvement of genetics healthcare services is not yet widely discussed or established and is equally relevant for medical geneticist and genetic counsellors’ professional groups.

**P32 –** INCIDENTAL DETECTION OF MATERNAL Xp22.31 DELETIONS AND DUPLICATIONS IN NONINVASIVE PRENATAL TESTING

Luís M. Pires^1^; Susana I. Ferreira^1^; Pedro Almeida^2^; Mariana Val^1^; Nuno Lavoura^1^; Fabiana Ramos^2^; Eulália Galhano^3^; Joana B. Melo^4^; Isabel M. Carreira^4^

^1^Laboratório de Citogenética e Genómica – Faculdade de Medicina da Universidade de Coimbra, Portugal; ^2^Serviço de Genética Médica, Centro Hospitalar e Universitário de Coimbra, Portugal; ^3^Centro de Diagnóstico Prénatal, Maternidade Bissaya Barreto, Centro Hospitalar e Universitário de Coimbra, Portugal; ^4^Laboratório de Citogenética e Genómica – Faculdade de Medicina da Universidade de Coimbra; CIBB-Centro de Inovação em Biomedicina e Biotecnologia, Universidade de Coimbra; iCBR CIMAGO –Centro Investigação em Meio Ambiente Genética e Oncobiologia, FMUC, P

**Introduction:** Non-invasive prenatal testing (NIPT) has been widely used to detect common fetal chromosome aneuploidies (T13, T18 and T21) and, eventually, sex chromosome aneuploidies. However, NIPT is only a screening method and therefore can lead to false positive or false negative results. (This is directly due to the NIPT techniques used that analyzes all the cfDNA circulating in the blood (maternal and fetal)). The free fetal cfDNA that circulates in maternal blood is of placental origin and may, in very rare situations, represent chromosomal alterations limited to the placenta, being the fetal karyotype normal. In the same way, maternal chromosomal abnormalities can also be detected, including X chromosome alterations with increased risks of pathogenic phenotype in male fetuses. In this work we report relevant sex maternal chromosomal abnormalities detected in our NIPTcohort.

**Methodology:** A retrospective analysis of 619 singleton pregnancies, with moderate risk for common fetal chromosome aneuploidies was performed.

**Results:** In this cohort, we identified 8 duplications in Xp22.31 and 1 deletion in the same region. This deletion is a 1.46M maternal Xp22.31 deletion, confirmed by aCGH, involving the *STS* ichthyosis gene linked to the X chromosome and reported on the OMIM Morbid Map. The child, at birth, showed signs of ichthyosis but, after 9 months of preprogrammed treatment he is currently healthy, apparently with normal development. The duplication in Xp22.31 region, were also confirmed by aCGH, with a frequency of 1.3% in our cohort. This is an interesting result, and the possibility of this variant having a high representation in the Portuguese population cannot be ruled out.

**Discussion:** We can conclude that NIPT is currently the best screening test to detect fetal common aneuploidies, with high specificity, but anomalies confined to the placenta, tumor abnormalities and incidental maternal genetic abnormalities can be a disadvantage leading to false positive or false negative results. For this reason, all abnormal NIPT results, including maternal incidental findings must always be confirmed by invasive diagnostic methods.

**P33 –** MUTATIONAL SPECTRUM AND GEOGRAPHIC DISTRIBUTION OF ALPHA-THALASSEMIA IN AN ADULT MICROCYTIC AND/OR HYPOCHROMIC POPULATION LIVING IN PORTUGAL: RESULTS FROM THE FIRST NATIONAL HEALTH EXAMINATION SURVEY (INSEF 2015)

Daniela Santos^1^; Irina Kislaya^2^; Pedro Lopes^1^; Carlos Matias- Dias^2^; Marta Barreto^2^; Paula Faustino^3^

^1^Departamento de Genética Humana, Instituto Nacional de Saúde Doutor Ricardo Jorge, Lisboa; ^2^Departamento de Epidemiologia, Instituto Nacional de Saúde Doutor Ricardo Jorge, Lisboa; Centro de Investigação em Saúde Pública, Escola Nacional de Saúde Pública, Universidade NOVA de Lisboa, Lisboa; ^3^Departamento de Genética Humana, Instituto Nacional de Saúde Doutor Ricardo Jorge, Lisboa; Instituto de Saúde Ambiental, Faculdade de Medicina, Universidade de Lisboa, Lisboa

Alpha-thalassemia (α-thal) is one of the most common monogenic disorders in the world. Its clinical severity varies from almost asymptomatic, mild microcytic hypochromic anemia, to a lethal hemolytic condition, depending on the number of affected α-globin genes (1 to 4). The disease is most commonly originated by deletions on 16p13.3. The aim of this study was to identify the molecular basis, geographic distribution and prevalence of mild forms of α-thal in Portugal.

This is a cross-sectional population-based study, based on the first Portuguese National Health Examination Survey (INSEF), which included individuals living in Portugal for more than 12 months, aged between 25 and 74 years old. For this INSEF sub-study, we analysed 4812 participants from whom a Complete Blood Count was performed and selected the 204 participants presenting red blood cell microcytosis (Mean Corpuscular Volume, MCV <80fL) and/or hypochromia (Mean Corpuscular Hemoglobin, MCH <27pg). DNA from these samples was used to search for deletions in *HBA* cluster by Gap-PCR and Multiplex Ligation-dependent Probe Amplification.

We found 52 individuals heterozygous for the −α^3.7kb^ deletion, one homozygous for this deletion and one heterozygous for the −α^4.2kb^ deletion. Two cases presented triplicated α-globin genes (ααα^anti 3.7kb^). Thus, α-thal was observed in 54 individuals (26.5%) of the analysed population. Carriers of the −α^3.7kb^ deletion have hypochromic red blood cells (MCH mean 26.0 ± 0.9 pg) but normal or borderline volume (MCV mean 81.4 ± 2.7 fL). The geographic distribution of affected participants showed two regions with highest prevalence of α-thal: LVT and RA Madeira.

Although the mild forms of α-thal themselves are of no clinical significance, their major importance is the modifying effect that they have on various severe forms of β-thalassemia and sickle cell disease. Furthermore, α-thal trait can be confused with iron deficiency anemia as the hematological parameters are quite similar. Therefore, iron status should be properly assessed to distinguish between the two conditions and α-thal confirmation at DNA level is necessary for a definitive diagnosis.

**P34 –** NON-INVASIVE PRENATAL TESTING IN PORTUGAL: RETROSPECTIVE STUDY IN LINE WITH THE 2020 ACOG/SMFM GUIDELINES

Isabel Alonso^1^; Isabel Miguel^1^; Vânia Reis^1^; Maria Lopes-de- Almeida^2^

^1^Genetyca-ICM, Porto, Portugal; ^2^Genetyca-ICM, Porto, Portugal and Unidade de Genética Médica, Hospital de Braga, Braga, Portugal

Non-invasive prenatal testing (NIPT) for fetal aneuploidies has emerged as an important tool in the antenatal care, particularly important in the management of high-risk pregnancies. The practice bulletin issued recently by the American College of Obstetricians and Gynecologists (ACOG) and the Society for Maternal-Fetal Medicine (SMFM) states that screening and diagnostic testing for chromosomal abnormalities should be offered early to all pregnant women irrespective of the maternal age and baseline risk. Here we present data collected between January 2018 and December 2019 that included 3130 pregnancies. In 42 (1.34%) of the cases an abnormal NIPT result was found; 29 fetuses with trisomy 21, 7 fetuses with trisomy 18 and 6 fetuses with sex chromosome aneuploidies. Chromosome 13 trisomy was not observed in this cohort. The NIPT result for trisomy 21 and 18 was confirmed in all cases by genetic testing of fetuses’ material obtained by amniocentesis or chorionic biopsy. Sex chromosome aneuploidies were confirmed in 3 of the 6 cases detected; one of the couples declined the diagnostic test and two X-chromosome monosomies were not confirmed. Interestingly, among the aneuploidies 18 and 21 identified, around 28% (10/36) occurred in women with less than 35 years old (8 fetuses with trisomy 21 and 2 fetuses with trisomy 18), 40% of the cases with no increased risk of fetal aneuploidy at the time of the test. PPV for trisomy 21 was of 100%, regardless the baseline fetal aneuploidy risk. The inconclusive rate, with no NIPT result after repeated sampling, was 0.19% (6 cases): in all but one the maternal age was above 35 years old and in half of the cases BMI was above 25. The inconclusive rate in our cohort is below the reported. Importantly, among the pregnancies with indication for NIPT due to a positive combined screening, more than 97% of the cases had a normal NIPT thus avoiding an invasive procedure. With the highest detection and lowest false-positive rates, cell-free DNA screening test proved to be a reliable prenatal test for chromosomal abnormalities with a positive outcome if applied to the general obstetric population.

**P35 –** MULTI-OMICS INTEGRATIVE PATHWAY ANALYSIS IN HEAD AND NECK SQUAMOUS CELL CARCINOMA

Luísa Esteves^1^; Ilda P Ribeiro^2^; Francisco Caramelo^3^; Isabel M Carreira^2^; Joana B Melo^2^

^1^1Cytogenetics and Genomics Laboratory, Faculty of Medicine, University of Coimbra, Coimbra, Portugal; ^2^1Cytogenetics and Genomics Laboratory, Faculty of Medicine, University of Coimbra, Coimbra, Portugal; 2iCBR, CIMAGO, Faculty of Medicine, University of Coimbra, Portugal; 3CIBB, Coimbra, Portugal, 4CACC, Coimbra, Portugal; ^3^2iCBR, CIMAGO, Faculty of Medicine, University of Coimbra, Portugal; 5Laboratory of Biostatistics and Medical Informatics, iCBR - Faculty of Medicine, University of Coimbra, Coimbra, Portugal

**Context:** Head and Neck Squamous Cell Carcinoma (HNSCC) is an aggressive disease that arises by molecular deregulation events including the accumulation of copy number alterations and changes in methylation profiles that result in modifications in gene expression levels and downstream signalling pathways. The study of these alterations is essential to understand the development and progression of HNSCC.

**Methods:** Copy number alteration (CNA), RNA-Seq and methylation data from tumor tissue from 416 HNSCC patients was downloaded from The Cancer Genome Atlas (TCGA). mRNA expression data for normal tumor- adjacent tissue was also retrieved. The genes contained in the regions altered in more than 40% of patients were extracted, for RNA-Seq data only the genes differentially expressed between tumour and normal tumor-adjacent tissue were used. A threshold of 0.3 was set for selecting methylated genes and those methylated in under 40% of patients were filtered out. The overrepresented pathways in each dataset were determined and those that were statistically significant (p < 0.01) and the respective genes altered in the cohort were analysed and compared between omics. R programming language was used for analysis.

**Results:** The top 3 most overrepresented signalling pathways were Pathways in Cancer, PI3K-Akt signaling pathway and Metabolic pathways when considering CNA data; Neuroactive ligand-receptor interaction, Olfactory transduction and Staphylococcus aureus infection in methylation data and Metabolic pathways, Axon Guidance and Cytokine-citokine receptor interaction when considering the RNA-Seq data. Two genes (*ADCY8* and *AGTR1*) were common to both CNA and methylation data pathways and *EGFR* was common to the latter and RNA-Seq data. For CNA and RNA-Seq data, a set of 8 genes was found to be common to the overrepresented signalling pathways.

**Conclusion:** This is a preliminary study that corroborates the complexity of molecular interactions that disrupt essential signalling pathways and give origin to HNSCC. The integration of multiple omics in the study of cancer is a hot topic that must be further explored in order to positively impact the clinical course of HNSCC patients.

**P36 –** THE PSYCHOSOCIAL EXPERIENCE OF MEMBERS OF FAMILIES WITH HEREDITARY AMYLOID TRANSTHYRETIN AMYLOIDOSIS WITH POLYNEUROPATHY: PRELIMINARY RESULTS OF A MIXED-METHODS SYSTEMATIC REVIEW

José D. Pereira^1^; Andreia Santos^2^; Eugenia Cisneros^3^; Intissar Anan^4^; Marina S. Lemos^5^; Milena Paneque^6^

^1^CGPP-Centro de Genética Preditiva e Preventiva and UnIGENe, IBMC- Instituto de Biologia Molecular e Celular, i3S-Instituto de Investigação e Inovação em Saúde, Universidade do Porto; Instituto de Ciências Biomédicas Abel Salazar, Universidade do Porto; ^2^Póvoa de Varzim, Porto, Portugal; ^3^Servicio de Medicina Interna, Hospital Universitario Son Llàtzer; Institut d’Investigació Sanitària Illes Balears, Hospital Universitario Son Espases; ^4^Institutionen för folkhälsa och klinisk medicin, Umeå universitet; ^5^Faculdade de Psicologia e de Ciências da Educação, Universidade do Porto; Centro de Psicologia, Universidade do Porto; ^6^CGPP-Centro de Genética Preditiva e Preventiva and UnIGENe, IBMC-Instituto de Biologia Molecular e Celular, i3S-Instituto de Investigação e Inovação em Saúde, Universidade do Porto

**Introduction:** Hereditary amyloid transthyretin amyloidosis with polyneuropathy, besides its chronicity and devastating progression, provokes a strong psychological impact on the life of these patients and their relatives. Thereby, genetic counsellors, psychologists and other health professionals are challenged to work together for the best possible care of those families. Aiming at promoting the development and delivery of clinically supportive services, we conducted a mixed-methods systematic review about the psychosocial experience of members of families with this condition.

**Methodology:** Manuscripts published between January 1992 and December 2019 were searched using 16 databases. The work includes a methodological quality assessment of selected studies, a postsynthesis sensitivity analysis, and an overall assessment of the thematic synthesis.

**Results:** Of 7,394 manuscripts identified, 220 were reviewed in full text and 70 met the eligibility criteria. Preliminary findings of the reviewed studies suggest that the disorder and its life implications may pose a significant psychosocial burden for the patients and their relatives. During their lifetime, members of these families generally became caregivers, implying changes in family roles, and parent's disease and death are frequent early in their life.

**Discussion:** Psychosocial experience of members of families with this disorder is not enough studied. Although scientific literature has described the life paths of these persons, further research on other key disease variables (e.g., including the psychosocial experience based on timing of clinical onset in the life cycle and effects of the most recent treatment interventions) can help fill research gaps and optimize health care services that support the families with the condition.

**Acknowledgments:** José D. Pereira has a doctoral grant (SFRH/BD/138012/2018), financed by the Fundação para a Ciência e a Tecnologia through the Human Capital Operational Programme, co- participated by the European Social Fund and by national funds from the Ministério da Ciência, Tecnologia e Ensino Superior.

Declaration of Interests: The authors declare no conflicts of interest.

**P37 –** A RARE MHC CLASS II HAPLOTYPE IS A MAJOR RISK FACTOR TO THE DEVELOPMENT OF EARLY ONSET TYPE I DIABETES IN A PORTUGUESE COHORT

Iris Caramalho^1^; Paula Matoso^1^; Dário Ligeiro^2^; Ana Laura Fitas^3^; Catarina Limbert^3^; Carlos Penha-Gonçalves^1^; Jocelyne Demengeot^1^

^1^Instituto Gulbenkian de Ciência, Oeiras, Portugal; ^2^Laboratório de Imunogenética, Centro de Sangue e Transplantação de Lisboa, Instituto Português de Sangue e Transplantação, Lisboa, Portugal; ^3^Unidade de Endocrinologia Pediátrica, Departamento de Pediatria, Hospital D. Estefânia, Lisboa, Portugal

Type 1 Diabetes (T1D) is a multifactorial disease with a strong genetic component that results from the immune- mediated destruction of pancreatic beta cells. Disease in preschool children, herein named Early-Onset (EO)T1D, is a rising and comparatively more severe clinical entity in the epidemiology of T1D in Western countries, including Portugal. The etiology of EOT1D remains unknown, which impedes the development of predictive diagnostic and the implementation of adequate preventive measures.

Genome-wide association studies established that approximately half of the T1D genetic risk is conferred by the Human Leukocyte Antigen (HLA) class II genes DRB1, DQA1 and DQB1 molecules. Moreover, several susceptibility and protective alleles/ haplotypes have been identified in T1D patients with an open-range age of disease onset. Whether the same or distinct risk alleles/haplotypes participate to the etiology of EOT1D remains, to the best of our knowledge, unknown.

The aim of this study was to examine the HLA-DR and DQ alleles as well as DRB1-DQA1-DQB1 haplotypes in 102 clinically well-characterized EOT1D patients (age at diagnosis ≤5 years), in comparison to a cohort of 100 T1D patients with disease onset between 8 and 35 years of age (Later onset (LaO)T1D). High-resolution HLA II typing was also performed in a cohort of 172 healthy controls.

Our results demonstrate that distinct HLA class II haplotypes contribute to the genetic landscape of EOT1D and LaOT1D. Moreover, we establish a major contribution of a rare HLA II haplotype, not previously associated to T1D in Europeans, to the development of T1D at a younger age in the Portuguese population.

**P38 –** RECOMMENDATIONS FOR *LDLR* VARIANT INTERPRETATION BY THE CLINGEN'S FAMILIAL HYPERCHOLESTEROLEMIA EXPERT PANEL

Joana Chora^1^; Michael Iacocca^2^; Lukas Tichy^3^; Hannah Wand^4^; Lisa C. Kurtz^5^; Heather Zimmermann^6^; Annette Leon Meredith^7^; Maggie Williams^8^; Steve E. Humphries^9^; Amanda J. Hooper^10^; Liam Brunham^11^; Alexandre da Costa Pereira^12^; Margaret Chen^13^; Jian Wang^14^; Mark Trinder^11^; Cinthia Elim Jannes^12^; Jessica Chonis^13^; Serra Kim^7^; Tina Pesaran^6^; Tami Johnston^6^; Alain Carrie^15^; Sarah Leigh^16^; Robert A. Hegele^17^; Eric Sijbrands^18^; Tomas Freiberger^19^; Joshua W. Knowles^20^; Mafalda Bourbon^1^

^1^Instituto Nacional de Saúde Doutor Ricardo Jorge, Lisbon, Portugal; BioISI, University of Lisbon, Lisbon, Portugal; ^2^Robarts Research Institute, Western University, London Ontario, Canada; Center for Inherited Cardiovascular Disease, Stanford University, Stanford California, USA; ^3^Center of Molecular Biology and Gene Therapy, University Hospital Brno, Brno, Czech Republic; ^4^Center for Inherited Cardiovascular Disease, Stanford University, Stanford California, USA; ^5^Department of Genetics, University of North Carolina, Chapel Hill, North Carolina, USA; ^6^Ambry Genetics, Aliso Viejo, California, USA; ^7^Color Genomics, Inc., Burlingame, California, USA; ^8^Bristol Genetics Laboratory, Bristol, United Kingdom; ^9^Centre for Cardiovascular Genetics, Institute of Cardiovascular Science, University College London, London, United Kingdom; ^10^Cardiovascular Genetics Laboratory, PathWest Laboratory Medicine, University of Western Australia, Perth, Australia; ^11^Department of Medicine, University of British Columbia, Vancouver, BC, Canada; ^12^Laboratory of Genetics and Molecular Cardiology, Instituto do Coração (InCor), Faculty of Medicine, São Paulo University, São Paulo, Brazil; ^13^GeneDx, Gaithersburg, Maryland, USA; ^14^Robarts Research Institute, Western University, London Ontario, Canada;; ^15^Hôpitaux Universitaires Pitié-Salpêtrière/Charles-Foix, Molecular and Chromosomal Genetics Center, Obesity and Dyslipidemia Genetics Unit, Sorbonne University, Paris, France; ^16^Genomics England, London, United Kingdom; ^17^Robarts Research Institute, Western University, London Ontario, Canada; ^18^Academic Medical Center, Erasmus University, Rotterdam, Netherlands; ^19^Centre for Cardiovascular Surgery and Transplantation, Brno, Czech Republic; ^20^Center for Inherited Cardiovascular Disease, Stanford University, Stanford California, USA; FH Foundation, Pasadena California, USA

Familial hypercholesterolemia (FH) is a highly prevalent (1:250) autosomal dominant genetic dyslipidaemia characterized by a lifelong exposure to elevated low-density lipoprotein cholesterol levels. The early identification and treatment of these patients is imperative for prevention of premature atherosclerotic cardiovascular disease.

With the widespread of affordable molecular genetic technologies, an increasing number of patients with a clinical suspicion of FH are being offered genetic testing as part of their diagnosis. Accordingly, the number of potentially disease-causing variants identified has also increased, and with it, the importance of correctly determine variant pathogenicity.

In 2015 the American College of Medical Genetics and Genomics and Association for Molecular Pathology (ACMG/AMP) published the standards for the interpretation of sequence variants. Shortly after, the Clinical Genome Resource has established expert panels to adjust these general guidelines to specific disease/gene pairs.

The ClinGen FH variant curation expert panel (VCEP) is composed of international expert clinicians, clinical laboratory diagnosticians, researchers and genomic medicine specialists working in FH. We have reviewed all scientific evidence, discussed each specification needed to thoroughly adapt the general ACMG/AMP guidelines to the FH and *LDLR* context and present here the consensus recommendations reached by all panel members.

The FH VCEP focused firstly on LDL receptor gene variants and specifications include indications for loss-of- function variant types; functional study criteria levels according to type of cells studied and thresholds to consider; definition of population data frequency values for benign and pathogenic; specific use and thresholds for *in silico* prediction tools; and co-segregation criteria specifications.

The FH VCEP has started classifying all ∼2800 *LDLR* variants currently published in ClinVar. Establishment of these guidelines as the new ‘gold standard’ in the FH community will help to achieve a consensus, accurate and standardized method for the clinical interpretation of variants identified in FH patients worldwide in a timely way.

**P39 –** GENETIC STATUS OF THE PORTUGUESE FH STUDY: VARIANT DESCRIPTION AND NEXT GENERATION SEQUENCING PANEL

Ana Margarida^1^; Ana Catarina Alves^1^; Mafalda Bourbon^1^

^1^Unidade de I&D, Departamento de Promoção da Saúde e Prevenção de Doenças Não Transmissíveis, Instituto Nacional de Saúde Doutor Ricardo Jorge; BioISI – Biosystems & Integrative Sciences Institute, Faculdade de Ciências, Universidade de Lisboa

**Introduction**: Familial Hypercholesterolemia (FH) is a common autosomal genetic disorder (1/250-1/500 worldwide). FH patients have high levels of cholesterol since birth with a family history of hypercholesterolemia and premature cardiovascular disease. Genetic diagnosis results from the study of 3 genes: *LDLR*, *APOB*, *PCSK9*. Recently, 5 genes have been associated with FH phenotype (*LDLRAP1*, *APOE*, *LIPA*, *ABCG5/8*) since pathogenic variants in those genes have been identified in clinical FH patients.

**Methodology**: Since 1999, 3106 individuals (1070 index cases with clinical FH diagnosis and 2036 relatives affected or non-affected) have been registered in the Portuguese FH Study. Until 2016, the genetic diagnosis was performed by Sanger sequencing of 3 FH genes. In 2017, an NGS panel of 8 genes (*LDLR*, *APOB*, *PCSK9*, *LDLRAP1*, *APOE*, *LIPA*, *ABCG5*, *ABCG8*) was implemented.

**Results:** We have identified 895 individuals (315 children and 580 adults) with a pathogenic or likely pathogenic in the FH genes: 885 heterozygous and 10 homozygous (3 true homozygous, 7 compound heterozygous). Additionally, variants were identified in other FH associated genes (*APOE*, 3 subjects) and in genes associated with recessive dyslipidemia: *LIPA* (4 homozygous, 13 heterozygous) and *ABCG5/8* (3 homozygous, 3 putative homozygous, 19 heterozygous). No pathogenic or likely pathogenic variants were identified in the remaining individuals.

**Discussion:** Overall, the Portuguese FH Study have identified 4.5% of the individuals expected to have FH in Portugal (prevalence of 1/500). A disease-causing variant was identified in 36% of the index-cases (381/1070). From these families, using a cascade screening approach, we identified 514 more subjects with FH. Cascade screening was performed in 73% of the families, but only 1 additional patient/index-case has been identified. This is an effective way to identify FH, but better cascade screening strategies need to be implemented in our country. In almost 1% of the index cases, a genetic cause for their dyslipidemia was found in FH associated genes (phenocopies) reinforcing the need to study these genes for a more accurate diagnosis and treatment of these individuals.

**P40 –** SILENCING *ATXN2* EXPRESSION USING CRISPR/CAS9

Ricardo António Afonso Reis^1^; Rebekah Koppenol^1^; Luís Pereira de Almeida^2^; Carlos A. Matos^1^; Clévio Nóbrega^1^

^1^Centre for Biomedical Research, Universidade do Algarve, Faro, Portugal; ^2^Center for Neuroscience and Cell Biology, Universidade de Coimbra, Coimbra, Portugal

**Introduction:** Spinocerebellar ataxia type 2 (SCA2) is a hereditary neurodegenerative disorder caused by expansion of CAG trinucleotide repeats present in the codifying region of the *ATXN2* gene. Mutant *ATXN2* product – atxn2 - displays a cytotoxic gain-of-function, leading to progressive neurodegeneration (1). No definitive therapy for this disease has yet been developed, but *ATXN2* knock-down represents a promising approach. The versatility of the CRISPR-Cas system enables gene silencing at a genomic level through an array of diverse mechanisms. Cas nucleases guided by a sgRNA produce modifications in precise regions of the DNA, and the system can be modified to direct transcriptional inhibitors such as the Krüppel associated box domain (KRAB) to particular genetic loci (2).

**Aims:** The aim of this study was to develop two *ATXN2* silencing strategies, utilizing dCas9-KRAB fusion protein to pre-transcriptionally repress *ATXN2* expression and catalytically active Cas9 to knock-down the *ATXN2* gene.

**Material and Methods:** One sgRNA targeting *ATXN2* promoter was cloned into plasmids encoding dCas9-KRAB, while another sgRNA targeting ATXN2 upstream of the CAG repeat sequence was cloned into a plasmid encoding Cas9. Plasmids were transfected into human embryonic kidney (HEK) 293 T cells and endogenous ataxin-2 mRNA and protein levels were analyzed by qPCR and Western blot.

**Results:** Cas9/sgRNA did not alter ataxin-3 mRNA and protein levels in comparison to controls. dCas9- KRAB/sgRNA did not alter ataxin-2 mRNA levels but displayed a tendency for decreasing ataxin-2 protein levels, relative to non-transfected cells (One-sample t test: *p* = 0.0534).

**Conclusion:** Our study suggests that the CRISPR-Cas9 system may be adapted for *ATXN2* gene silencing applications, but further improvements to our strategies will be necessary to produce significant alterations.

**References:** 1 – Velázquez-Pérez L *et al*. *Front Neurol*. 2017; 8:472; 2 –Gilbert L *et al*. *Cell.* 2013; 154:442–51.

**Acknowledgements**: CN laboratory is supported by AFM-Téléthon, Ataxia UK, the National Ataxia Foundation and the Fundação para a Ciência e Tecnologia.

Declaration of interests: The authors declare no conflict of interest.

**P42 –** SEMEN PARAMETERS, DNA FRAGMENTATION AND SPERM ANEUPLOIDY EVALUATION IN THE ETIOLOGY OF MALE INFERTILITY

Miguel Maia^1^; Carolina Almeida^2^; Mariana Cunha^3^; Ana Gonçalves^3^; Sandra Silva-Soares^4^; Milton Severo^5^; C. Joana Marques^2^; Mário Sousa^6^; Alberto Barros^7^; Sofia Dória^2^

^1^Unidade de Genética, Departamento de Patologia, Faculdade de Medicina, Universidade do Porto, Portugal; ^2^Unidade de Genética, Departamento de Patologia, Faculdade de Medicina, Universidade do Porto, Portugal; i3s – Instituto de Investigação e Inovação em Saúde, Universidade do Porto, Portugal; ^3^Centro de Genética e Reprodução Professor Alberto Barros, Porto, Portugal; ^4^Unidade de Medicina da Reprodução, Centro Hospitalar Universitário São João (CHUSJ), Porto, Portugal; ^5^EPIUnit - Instituto de Saúde Pública, Universidade do Porto, Portugal; Departamento de Ciências da Saúde Pública e Forenses e Educação Médica, Faculdade de Medicina, Universidade do Porto, Porto, Portugal; ^6^Departamento de Microscopia, Instituto de Ciências Biomédicas Abel Salazar, Universidade do Porto, Portugal; ^7^Unidade de Genética, Departamento de Patologia, Faculdade de Medicina, Universidade do Porto, Portugal; i3s – Instituto de Investigação e Inovação em Saúde, Universidade do Porto, Portugal; Centro de Genética e Reprodução Professor Alberto Barros, Porto

**Backgroud:** Infertility is caused by several factors. The study of the infertile couple is extremely important to achieve a correct diagnostic and, consequently, a better treatment. Considering male infertility, studies beyond sperm parameters evaluation such as sperm aneuploidies and sperm DNA fragmentation could also be important. Some studies were performed to understand the clinical utility of these DNA quality tests, however implementation in routine diagnosis have not yet been performed. The main goal of this study was to evaluate if sperm aneuploidies and sperm DNA fragmentation should be included as valid tests in the routine investigation of male infertility. Additionally, we aimed to define a cut-off value above which significantly increased sperm DNA fragmentation can compromise male fertility.

**Materials and Methods:** 835 infertile individuals (individual or referred as couple infertility) from 2007 to 2019 were included. Semen samples were investigated for conventional semen parameters, sperm DNA fragmentation using Terminal deoxynucleotidyl transferase dUTP nick- end labelling (TUNEL) and sperm aneuploidies by Fluorescence *in situ* Hybridization (FISH).

**Results and Discussion:** Male age seemed to trigger sperm DNA fragmentation. For oligozoospermic men and individuals with abnormalities in association, sperm DNA fragmentation analysis showed to be a relevant test. On the other hand, oligoteratozoospermic (OT) or oligoasthenoteratozoospermic (OAT) men seem to benefit from sperm aneuploidies testing. A statistically significant and positive association between sperm DNA fragmentation and sperm aneuploidies was also found. Additionally, a cut-off point of 18.8% of sperm DNA fragmentation was established, using TUNEL-assay by fluorescence microscopy.

**Conclusion:** This study helped to understand in which cases should be performed sperm aneuploidies or sperm DNA fragmentation tests for routine investigation of male infertility and allowed to recommend a new cut-off for sperm DNA fragmentation as the reference value above which male fertility status could be seriously compromised. This contributes for a better reproductive counselling to infertile couples.

**P43 –** THE EMERGING ROLE OF *CTNNA1* AS A HEREDITARY DIFFUSE GASTRIC CANCER PREDISPOSING GENE

Silvana Lobo^1^; Carla Oliveira^1^

^1^i3S - Instituto de Investigação e Inovação em Saúde

**Introduction:** Hereditary Diffuse Gastric Cancer (HDGC) predisposes for diffuse gastric cancer (DGC) and/or lobular breast cancer (LBC). HDGC is caused by *CDH1* inactivating mutations, and by *CTNNA1* truncating variants. *CTNNA1* is a recent HDGC-associated gene, encoding α-E-catenin, which associates with cadherins cytoplasmic domain. *CTNNA1* germline variants also cause patterned macular dystrophy-2.

**Methods:** We systematically searched the literature to identify families bearing *CTNNA1* germline variants with or without HDGC criteria, classified the variants according to the ACMG guidelines and analyzed genotype-phenotype associations.

**Results:** We found 41 families bearing *CTNNA1* germline variants, 13/41 fulfilling HDGC criteria. 10/13 (77%) families carried pathogenic (P) variants: 4 nonsense, 6 frameshift. All 10 probands had DGC (average ≈40y.o). From their 31 relatives: 14 had DGC, 1 LBC, 5 gastric cancer (GC), 2 breast cancer (BC) and 8 other cancer types. From the 28 families lacking HDGC criteria, only 14 (50%) carried P or likely pathogenic (LP) variants: 7 frameshift, 6 nonsense and 1 large deletion. Five out of 14 probands were healthy, 1 had ductal BC (DBC) and 8 had unspecified BC (average = 51y.o.). From their 21 relatives: 3 had GC (average > 60y.o.) and 18 had BC (average = 56y.o.). Six additional families lacking HDGC criteria presented truncating variants (5 frameshift, 1 nonsense) in the last exon of the gene classified as VUS. These 6 probands presented: 2 BC (average >60y.o.), 3 DBC (average = 45y.o.) and 1 was healthy.

**Discussion:** We confirm *CTNNA1* truncating P/LP variants as a HDGC predisposing factor. In families fulfilling HDGC clinical criteria, the frequency of P/LP variants is higher than in those lacking criteria, and the main phenotype is early-onset DGC. In contrast most families lacking criteria and carrying P/LP variants do not present HDGC-related phenotypes. These results hint to *CTNNA1* additional modifiers effects in triggering/protecting for HDGC-specific phenotypes. Because truncating variants in the last exon only appear in non-HDGC families supports the formation of partially functional truncated proteins escaping NMD.

**P44 –** UNCOVERING THE ETIOLOGY OF ISOLATED SHORT STATURE WITH MILD SKELETAL FINDINGS

André M. Travessa^1^; Patrícia Dias^1^; Lucia Sentchordi-Montané^2^; Miriam Aza-Carmona^2^; Silvia Modamio-Høybjør^2^; Catarina Ferreira^3^; Belinda Campos-Xavier^3^; Sónia Custódio^1^; Rosário Silveira-Santos^1^; Ana Sousa^1^; Andrea Superti-Furga^3^; Karen E. Heath^2^; Ana Berta Sousa^1^

^1^Medical Genetics Department, Hospital de Santa Maria, Centro Hospitalar Universitário Lisboa Norte, Centro Académico de Medicina de Lisboa, Lisbon, Portugal; ^2^Institute of Medical and Molecular Genetics (INGEMM) and Skeletal Dysplasia Multidisciplinary Unit (UMDE), Hospital Universitario La Paz, Universidad Autonóma de Madrid, IdiPAZ, and CIBERER, ISCIII, Madrid, Spain; ^3^Division of Genetic Medicine, Lausanne University Hospital (CHUV), University of Lausanne, Lausanne, Switzerland

**Introduction:** Besides *SHOX*, an increasing number of genes (such as *ACAN*, *FGFR3*, *IHH*, *NBAS*, *NPPC*, and *NPR2*) have been identified in patients with isolated short stature (SS) with or without mild skeletal findings.

**Objective:** To investigate the etiology of SS in patients with isolated SS and mild skeletal findings.

**Methodology:** In nine probands with height below the 3rd centile and mild skeletal findings who were negative for *SHOX* deletions, we performed *SHOX* Sanger sequencing and/or NGS-based strategies (skeletal dysplasia panel or whole-exome sequencing).

**Results:** We found monoallelic pathogenic or likely pathogenic variants in *IHH* in two probands, in *ACAN* in one proband, and in *SHOX* in another proband. In three cases, family history of SS was compatible with an autosomal dominant pattern of inheritance. Although *IHH*, *ACAN* and *SHOX* may present with specific skeletal findings, none were found in these patients. The variant identified in *ACAN* was described in another family with SS and *osteochondritis dissecans*, but the latter was not present in our patient and her family. The other variants are novel.

Moreover, we found biallelic pathogenic variants in *NBAS* in one patient. Patients with *NBAS* variants also usually present with additional findings. After molecular diagnosis, the patient was shown to have Pelger-Huet anomaly.

Of the negative cases (44%), two patients had a family history of SS, suggesting autosomal recessive inheritance in one and autosomal dominant inheritance in the other.

**Discussion:** In our cohort, NGS-based strategies led to a genetic diagnosis in 56% of patients with isolated SS and mild skeletal findings. This yield is high in comparison with the literature, probably due to the significant number of familial cases and the severity of SS. In four cases, molecular diagnosis would not have been possible only on the basis of the clinical characteristics. Besides patients with *SHOX* variants, some reports suggest benefits of growth hormone in patients with *ACAN* and *IHH* variants, but larger studies are needed to confirm this growth potential. Further research is required to understand the genetics of isolated SS with or without mild skeletal findings.

**P45 –** BLENDED PHENOTYPES: AN UNUSUAL EXPRESSION OF GENETIC HETEROGENEITY

João Parente Freixo^1^; Jorge Oliveira^1^; Rita Quental^2^; Paulo Silva^1^; Susana Sousa^1^; Daniel Gonçalves^3^; Carla Moura^2^; Miguel Leão^2^; Jorge Sequeiros^1^

^1^Centro de Genética Preditiva e Preventiva, Instituto de Biologia Molecular e Celular, Instituto de Investigação e Inovação em Saúde, Universidade do Porto; ^2^Serviço de Genética Médica, Centro Hospitalar Universitário de São João, Universidade do Porto, Porto, Portugal; ^3^Serviço de Pediatria, Centro Hospitalar Universitário de São João, Universidade do Porto, Porto, Portugal

**Introduction:** During recent years, DNA sequencing capacity has considerably improved genetic diagnostic rate, contributed to identification of new genetic causes of rare monogenic diseases, and challenged our perception about the extension and complexity of some phenotypes. Comprehensive genomic approaches, clinical exome and whole-exome sequencing (WES) have shown that, in a small subset, the presented phenotype is the product of concomitant monogenic disorders in a single patient, giving rise to so-called ”blended phenotypes”.

**Methodology:** We used a WES approach in 4,027 individuals referred for genetic diagnostic study at CGPP (2016-2019), for targeted disease specific multigene panels (n = 3,284); or for clinical exome or analysis of WES in a trio (n = 743), in clinically heterogeneous presentations. We systematically reviewed the genetic data of these 743 cases, either (singleton) clinical exome (n = 629) or WES in trio (patient and both parents) (n = 114). Patients showing variants classified as pathogenic or likely pathogenic, in at least two different genes, and data (at least partly) compatible with phenotype and known inheritance pattern for their disease, were reassessed.

**Results:** Five patients had two genetic conditions: two dominant diseases were found in 3 cases; 2 patients had an autosomal recessive and a dominant disease. Though parental studies were not possible for all cases, of note is that 2 patients had *de novo* variants occurring in two differentgene-pairs: *HUWE1* and *CHAMP1*, and *SETD5* and *KIF21A*. Frequency of the blended (overlapping or combined) phenotypes was ∼0.67% in our cohort (n = 743).

**Discussion:** This work further expands knowledge about blended phenotypes as five additional cases are reported. Despite its overall low frequency (likely to be underestimated), the awareness about such conditions is important for diagnostic and genetic counselling purposes.

**P46 –***CUL4B*-RELATED DISORDERS: FROM AN ATYPICAL PRESENTATION IN A MALE WITH CABEZAS SYNDROME TO NOVEL DESCRIPTIONS OF *CUL4B*-RELATED PHENOTYPES IN MALES AND A FEMALE

Célia Azevedo Soares^1^; Cláudia Falcão Reis^2^; Ana Maria Fortuna^1^; Gabriela Soares^3^; Natália Tkachenko^4^

^1^Serviço de Genética Médica, Centro de Genética Médica Jacinto Magalhães, Centro Hospitalar Universitário do Porto, Porto, Portugal & UMIB, ICBAS/UP, Porto, Portugal; ^2^Serviço de Genética Médica, CGM/CHUP, Porto, Portugal; Life and Health Sciences Research Institute (ICVS), School of Medicine, University of Minho, Braga, Portugal ICVS/3B's - PT Government Associate Laboratory, Braga/Guimarães, Portugal; ^3^Consulta de Genética Médica, Hospital S. Pedro, Centro Hospitalar de Trás-os-Montes e Alto Douro, Vila Real, Portugal; ^4^Serviço de Genética Médica, Centro de Genética Médica Jacinto Magalhães, Centro Hospitalar Universitário do Porto, Porto, Portugal

**Introduction***: CUL4B*, located on the X chromosome, codes cullin-4B a major regulator of key cellular functions by controlling protein degradation. *CUL4B* loss of function (LoF) variants are well established as causal of Cabezas syndrome (CS), an X-linked intellectual disability syndrome with dysmorphic features, known to affect only males. We aim to show the complexity of the phenotypes related to *CUL4B*, describing a male patient with an atypical phenotype for CS and novel phenotypes associated with *CUL4B*, beyond the classical description of CS.

**Methodology:** Clinical revision of four patients with relevant variants in *CUL4B* in our Medical Genetics unit.

**Results:** One male patient presented with a *CUL4B* deletion and typical CS features: severe intellectual disability, absent speech, seizures, dolichocephaly, and bitemporal narrowing. This patient presented additionally some atypical features: severe lipodystrophy, and disproportionate short stature. Further studies are ongoing to exclude a multiloci phenotype.

Two male brothers presented with *CUL4B* non-disruptive duplication inherited from their asymptomatic mother. qPCR revealed *CUL4B* overexpression. Both males presented with borderline intellectual disability and no major dysmorphic features, a phenotype less severe than that associated with *CUL4B* LoF.

A female patient presented with phenotypic features similar to CS: severe intellectual disability, absent speech, seizures, dolichocephaly, and bitemporal narrowing. ArrayCGH identified a *de novo* partial *CUL4B* gene deletion. X- chromosome inactivation studies were non-informative. Whole exome sequencing and 15q11-13 msMLPA were normal. This patient is part of an international collaborative effort to establish a new phenotype related to *CUL4B* LoF in females.

**Discussion:** These patients description highlights the complexity of *CUL4B*-related phenotypes. LoF is not the only mechanism associated with *CUL4B-*related intellectual disability, as shown by our patients with *CUL4B* duplication. Skewed X- chromosome inactivation is a putative mechanism for CS in a female. This cohort description opens the discussion for the consolidation of new CUL4B-related entities beyond CS.

**CLINICAL CASES**

**P47 –** DETECTION OF CHROMOSOME 3p DUPLICATION WITH ADJACENT TERMINAL DELETION IN PRENATAL DIAGNOSIS

Mariana Val^1^; Susana I. Ferreira^1^; Luís M. Pires^1^; Nuno Lavoura^1^; Alexandra Mascarenhas^1^; Cláudia Pais^1^; Isabel M. Carreira^2^; Joana B. Melo^2^

^1^Laboratório de Citogenética e Genómica, Faculdade de Medicina da Universidade Coimbra, Coimbra, Portugal; ^2^Laboratório de Citogenética e Genómica, Faculdade de Medicina da Universidade Coimbra, Coimbra, Portugal; iCBR-CIMAGO – Centro de Investigação em Meio Ambiente, Genética e Oncobiologia, Faculdade de Medicina da Universidade Coimbra, Coimbra, Portugal

**Introduction:** Chromosome 3pter-3p25 deletion syndrome is characterized by low birth weight, microcephaly, craniofacial dysmorphism, cardiac, renal and gastric anomalies, polydactyly, hypotonia and intellectual disability (ID). Regarding distal trisomy 3p, craniofacial dysmorphism, psychomotor delay, ID, seizures, cardiac, urogenital and brain anomalies are frequently observed. The clinical features in cases with distal trisomy 3p and adjacent 3pter-3p25 deletion, a very rare condition, should be a combination of both disorders. Case report: We present a 27-year-old primigravida, referred to prenatal diagnosis consultation due the detection of truncus arteriosus on fetal ultrasound performed at 21 weeks of gestation. Amniocentesis was performed. Rapid aneuploidy detection test was done by quantitative fluorescent PCR (QF-PCR). aCGH (Agilent 4x180K) and conventional cytogenetics were performed to search for chromosomal abnormalities.

**Results:** QF-PCR results revealed a male fetus and aneuploidies for chromosomes 13, 18, 21 were not detected.

aCGH analysis revealed a 9.76Mb pathogenic deletion at 3p26.3p25.3 and a contiguous 6.65Mb pathogenic duplication at 3p25.3p24.3. A 1.3Mb duplication of uncertain significance at 16q23.1 was also detected. The analysis of metaphases by GTG banding showed loss of material at 3pter and additional material at the adjacent 3p region of the same homologue chromosome 3. The pregnancy was subsequently terminated.

**Discussion:** According to literature, the majority cases with terminal deletion and concurrent adjacent duplication shows an inverted duplication, the most likely rearrangement occurring in the present case, although the orientation of duplication was not determined. The U-type exchange after a double strand break of the two sister chromatids is referred as the most frequent mechanism for these rearrangements. The present case, as the 3 cases reported with 3p inverted duplication and adjacent 3pter deletion, showed congenital heart defects, common in both 3pter-3p25 deletion syndrome and distal trisomy 3p. Moreover, our results highlight the importance of combining distinct techniques to study rare chromosomal rearrangements.

**P48 –** A PRENATAL CASE OF PARTIAL MONOSOMY 18(q21.3-q23), RESULTING FROM A MATERNAL INSERTIONAL TRANSLOCATION ins(12;18) UNCOVERED BY ARRAY-CGH AND KARYOTYPING

Ana Jardim^1^; Susana Ferreira^1^; Eunice Matoso^2^; Joana B. Melo^3^; Isabel M. Carreira^3^

^1^Laboratório de Citogenética e Genómica, Faculdade de Medicina da Universidade de Coimbra; ^2^Laboratório de Citogenética, Serviço Genética Médica, Hospital Pediátrico, CHUC (Centro Hospitalar e Universitário de Coimbra); iCBR– Area of Environment, Genetics and Oncobiology (CIMAGO); ^3^Laboratório de Citogenética e Genómica, Faculdade de Medicina da Universidade de Coimbra; iCBR – Area of Environment, Genetics and Oncobiology (CIMAGO), CIBB - Center for Innovative Biomedicine and Biotechnology

**Introduction:** Balanced insertional translocations (ITs) refer to the intercalation of a part of one chromosome into another non-homologous chromosome or into another part of the same chromosome 1. Therefore, the imbalances that result from segregation of a balanced, interchromosomal IT can be a pure segmental monosomy or trisomy1. Insertional rearrangements imply one of the highest reproductive risk, reaching theoretically 50%1. Cytogenetically visible ITs are rare chromosome rearrangements with an incidence of about 1:80,000 live births.

**Case Report:** We report a prenatal case referred because of single umbilical artery, ventriculomegaly and nasal bone length smaller than the 5th percentile. Array-CGH revealed a 17,3Mb intersticial deletion on the long arm of chromosome 18 and another interstitial deletion of approximately 489kb on the long arm of chromosome X. After array-CGH was done on the parents, both alterations were found to be *de novo*. Conventional cytogenetic evaluation of the parents allowed the diagnosis of a balanced insertional translocation of the segment 18(q21.3-q23) of the long arm of chromosome 18 in the long arm of chromosome 12 (band q24.31) in the mother.

**Discussion:** This case highlights the importance of the combination of two or more techniques and it emphasizes the relevance of parental testing to achieve a correct diagnosis. In this case only array-CGH and conventional karyotyping were needed but molecular cytogenetics (FISH) could also have been required. Achieving an accurate diagnosis in these cases is critical for a more precise genetic risk assessment, an improved management of future pregnancies, including a referral for preimplantation genetic diagnosis (PGD) and also allows the pursuit of further family studies.

^1^ Nowakowska BA, de Leeuw N, Ruivenkamp CA, et al. Parental insertional balanced translocations are an important cause of apparently *de novo* CNVs in patients with developmental anomalies. *Eur J Hum Genet*. 2012; 20:166–170.

**P49 –** PHELAN-MCDERMID SYNDROME AND NEUROFIBROMATOSIS TYPE 2: CONNECTED BY A RING CHROMOSOME 22

Mariana Tomásio Neves^1^; Patrícia Dias^1^; Juliette Dupont^1^; Oana Moldovan^1^; André Travessa^1^; Sónia Custódio^1^; Eva Rolo^1^; Raquel Rodrigues^1^; Rosário Santos^1^; Ana Sousa^1^; Ana Berta Sousa^1^

^1^Serviço de Genética, Departamento de Pediatria, Hospital de Santa Maria, Centro Hospitalar Universitário Lisboa Norte, Lisboa, Portugal

**Introduction:** Phelan-McDermid syndrome (PMS) is characterized by neonatal hypotonia, global developmental delay (DD), intellectual disability (ID), absent or severely delayed speech, autism spectrum disorder and minor dysmorphic facial features. PMS results from haploinsufficiency of *SHANK3* gene caused by a microdeletion in 22q13.3 or by a pathogenic sequence variant. In a minority of PMS patients there is an increased risk of Neurofibromatosis type 2 (NF2). This occurs when the chromosomal imbalance is associated with a ring chromosome resulting from the fusion of both arms at terminal deletion breakpoints, which leads to mitotic instability of the adjacent 22q12.2 region encompassing *NF2* gene. The development of NF2 in PMS patients is explained by a two-hit carcinogenesis model. Individuals with PMS associated with a terminal deletion should perform a karyotype to ascertain the presence of a ring chromosome.

**Methodology:** Four females (aged 9, 11, 15 and 29 years old) and one male (aged 15 months) were referred to medical genetics due to global DD/ID and behavioral problems. All performed aCGH as first line genetic test with the identification in each of a heterozygous de *novo* 22q12.3 deletion, involving *SHANK3* gene and compatible with a PMS diagnosis. Subsequently, karyotypes were performed to exclude the presence of a ring chromosome **22.**

**Results:** At physical examination none of the patients showed abnormalities suggestive of NF2. Two karyotypes are still ongoing. Karyotyping revealed a ring chromosome 22 in one of the remaining three patients.

**Discussion:** NF2 is an autosomal dominant inherited tumour predisposition syndrome associated with increased risk of schwannomas (mainly of the vestibular nerve causing deafness), meningiomas, ependymomas and cutaneous neurofibromas. Recommended surveillance includes periodical neurological examination and imaging of the central nervous system. Patients with PMS caused by a terminal deletion in 22q13.3 identified by aCGH should be evaluated with karyotype to exclude a ring chromosome. Carriers of a ring chromosome 22 should follow the protocols for NF2 surveillance.

**P50 –** A NIPT ANEUPLOIDY SUSPITION WITH NORMAL QF-PCR AND aCGH. KARYOTYPE GIVES THE ANSWER

Marta Pinto^1^; Luís M. Pires^1^; Susana Ferreira^1^; Patrícia Paiva^1^; Ana Jardim^1^; Joana B. Melo^2^; Isabel M. Carreira^2^

^1^Laboratório de Citogenética e Genómica, Faculdade de Medicina da Universidade de Coimbra, Portugal; ^2^Laboratório de Citogenética e Genómica, Fac. Med. Univ. Coimbra; iCBR-CIMAGO – Centro de Investigação em Meio Ambiente, Genética e Oncobiologia, Fac. Medicina Univ. Coimbra; CIBB - Centro de Inovação em Biomedicina e Biotecnologia, Fac. Med. Univ. Coimbra

**Introduction:** Advances in molecular tests brought greater capacity for genetic diagnosis. Nevertheless, even with these higher resolution tests, the identification of mosaics may still be a complex process, remaining the mosaicism a challenge for clinical diagnosis and genetic counselling. We report a mos 45,X/47,XXX fetus with an inconclusive result after a non invasive prenatal test (NIPT) and normal results by QF-PCR and oligoarray-CGH (aCGH).

**Case Report:** A pregnant woman was referred for NIPT due to an increased risk for trisomy after first trimester screening test. Normal results were obtained for chromosomes 13, 18 and 21 but were inconclusive for sexual chromosomes, indicating a possible aneuploidy involving the X chromosome in a female fetus. The geneticist decided to request QF-PCR for common aneuploidies and aCGH in an amniotic fluid sample, which revealed, in both tests, a female fetus with normal results. Since there was a strong suspicion of sexual aneuploidy, as laboratory internal quality control, metaphases were analysed revealing a mos 45,X[11]/47,XXX[9] karyotype.

**Discussion and Conclusions:** The NIPT result, although inconclusive for the sexual chromosomes, it was suggestive of an aneuploidy, partial or complete, involving the X chr in a female fetus. The karyotype result explains the inconclusive NIPT and also explains the normal results observed with the other two molecular techniques, since it identified a 50:50 X/XXX mosaic. The QF-PCR showed two alleles for the X chr and the percentage of each cell line explains why no aneuploidy was identified. The normal result by aCGH is, also, due to normalization of ratios between the patient and control samples.

This case point out the fact that despite we are in the genomic era, the karyotype is still an important tool and, in this particular case, it allowed a genetic diagnosis that, otherwise, would be missed.

The case also highlights the role of Clinical Laboratory Geneticist (CLG) to assist the Medical Geneticist in the best laboratory strategy of study in order to achieve the most accurate diagnosis and genetic counselling.

**P51 –** INTERSTITIAL 6q DUPLICATION - A SUPPORT FOR THE CANDIDACY OF *PLN* GENE AS A CAUSE OF CONGENITAL HEART DISEASE

Alexandra Mascarenhas^1^; Patrícia Paiva^1^; Mariana Val^1^; Isabel Marques Carreira^2^; Joana Barbosa de Melo^2^

^1^Laboratório de Citogenética e Genómica, Faculdade de Medicina, Universidade de Coimbra, Portugal; ^2^Laboratório de Citogenética e Genómica, Fac. Med., Univ. Coimbra; iCBR-CIMAGO–Centro de Investigação em Meio Ambiente, Genética e Oncobiologia, Fac. Med., Univ. Coimbra; CIBB-Centro de Inovação em Biomedicina e Biotecnologia, Fac. Med., Univ. Coimbra

**Introduction:** Congenital heart disease (CHD) is a very common birth defect and is often associated with other anomalies, such as dysmorphic features, and developmental delay, being a revelant cause of infant morbidity and mortality. Advancements in molecular techniques, namely, microarray comparative genomic hybridization analysis (aCGH) allow the identification of submicroscopic genomic rearrangements, detecting CNVs in patients with CHD. Interstitial duplication of the long arm of chromosome 6 is a rare event and phenotipically very variable, depending on the location, size and involving genes.

**Case Report:** We present a 2 year old girl with short stature, Pierre Robin sequence and cardiopathy. Genetic analysis by array aCGH showed a 312Kb dup(6)(q22.31), encompassing the non-coding gene *BRD7P3* and *PLN* and *CEP85L* genes, both described in the OMIM Morbid Map. Few smaller CNVs in duplication have been classified as benign and several similar or longer CNVs have been described as uncertain clinical significance. In the literature there is only one report, with a similar duplication, in a patient with transposition of the great arteries and atrial and ventricular septal defect, being the *PLN* gene suggested as a candidate for CHD.

**Conclusion:** Partial duplication of chromosome 6q with phenotypic manifestations is a rare event and with a great genomic heterogeneity. Recently *CEP85L* gene has been associated with neurologic defects. Mutations in *PLN* gene, that encodes de phospholamban protein, involved in calcium signalling and muscle contraction, are associated with hypertrophic and dilated cardiomyopathy. Some studies demonstrate that an increase in *PLN* expression is associated with heart defects.

The development of new technologies including copy number variants, and the correlation with phenotypic manifestations, are expanding our knowledge of genetic causes of heart anomalies. In this context, our study underlines the importance of *PLN* gene alterations as one of the candidates for CHD.

**P52 –** 3p26.3 REGION - WHAT LIES BENEATH

Ana Sousa^1^; Sónia Custódio^1^; Rosário Silveira-Santos^1^; Raquel Rodrigues^1^; João Alves^1^; Mariana Soeiro Sá^1^; André Travessa^1^; Patrícia Dias^1^; Oana Moldovan^1^; Ana Berta Sousa^1^

^1^Serviço de Genética Médica, Departamento de Pediatria, Hospital de Santa Maria, Centro Hospitalar Universitário Lisboa Norte EPE, Centro Académico de Medicina de Lisboa

**Introduction:** CNVs involving 3p26.3 region have been associated with neurodevelopmental disorders. This region contains genes encoding neuronal cell adhesion molecules which play a role in the formation of axon connections during nervous system development: *CHL1*, *CNTN6*, and *CNTN4*. We reviewed the clinical and genomic information of a 3p26.3 CNVs cohort in order to ascertain the region clinical burden.

**Methods:** We retrospectively reviewed eight patients carrying 3p26.3 CNVs, identified by *a*CGH: three deletions and six duplications (one patient harboured two duplications). Two deletions are single gene involving either *CHL1* or *CNTN6,* while the third one encompasses both *CNTN6* and *CNTN4.* In the duplication group, three are single gene, one involving *CNTN6 and* two involving *CNTN4*. The remaining three include a duplication encompassing *CNTN6 a*nd *CHL1,* and two contiguous duplications of *CNTN6* and *CNTN4*. One deletion and three duplications were inherited from unaffected parents.

**Results/Conclusion:** Deletion carriers presented mild/moderate ID (2/3), ASD (2/3), speech delay (1/3), epilepsy (1/3), and dysmorphic features (1/3). Duplication carriers exhibited a more heterogeneous phenotype, also including mild DD/ID (4/5), speech delay (2/5), epilepsy (1/5), and dysmorphic features (1/5). Single gene CNVs are particularly valuable for understanding the effects of gene dosage alterations: a milder phenotype was observed for the *CNTN6* duplication when compared to the reciprocal deletion; *CHL1* deletion is likely responsible for the observed ASD, speech delay and dysmorphisms; *CNTN4* duplications are associated with DD/ID and speech delay. Our data is comparable to previously reported cases and suggests that *CNTN6*, *CNTN4* and *CHL1* are dosage-sensitive genes with a central role in cognitive development. Incomplete penetrance is widely described justifying the existence of unaffected carrier parents. Variable expressivity and reduced penetrance are especially challenging in genetic counseling. To further investigate the genotype-phenotype correlation of *CNTN6*, *CNTN4* and *CHL1* CNVs, we propose putting together a national cohort to help clarify “what lies beneath” 3p26.3 region.

**P53 –** PRENATAL DIAGNOSIS OF 2q37 DELETION SYNDROME ASSOCIATED WITH A *DE NOVO* UNBALANCED KARYOTYPE

Joana Trindade^1^; Cláudia Alves^1^; Fernando Lopes^2^; Rita Monteiro^1^; Mafalda Lopes^1^; Fernanda Baltar^1^; Alina Queirós^1^; Maria João Oliveira^1^; Cecília Correia^1^; Margarida Reis-Lima^1^

^1^Unidade de Citogenética, SYNLABHEALTH Genética Médica, Porto; ^2^Unidade Medicina Fetal, Hospital de S. Teotónio, Viseu

**Introduction:** Patients with chromosome 2q37 deletion syndrome or brachydactyly-mental retardation syndrome (BDMR) (OMIM 600430), or Albright's hereditary osteodytrophy-like (AHO-like) syndrome show a highly variable spectrum of clinical manifestations, that can result from different deletion sizes and gene content. This syndrome has been associated with deletion or heterozygous mutation in the *HDAC4* gene.

Common clinical features include type E brachydactyly and facial dysmorphism, obesity and autism spectrum disorders.

Short stature, mild to moderate intellectual disability and behavioural problems have also been described.

This syndrome has rarely been reported in prenatal diagnosis, and has been associated with central nervous system (CNS) abnormalities and intrauterine growth restriction (IUGR), among others.

**Methodology:** A 37-year-old healthy pregnant woman was referred for amniocentesis at 21 weeks of gestation, due to fetal CNS malformations (slight ventriculomegalia, braquicephaly) and increased nuchal translucency in 1st trimester.

Aneuploidy screening, CGH microarray (60K) and karyotype analysis (GTL bands) were performed.

**Results:** Microarray CGH-array analysis revealed a pathogenic copy number loss at chromosome 2q37.1q37.3. Fetal karyotype showed an unbalanced chromosome rearrangement involving chromosomes 2 and 7, with loss of the distal 2q37 segment: 46,XX,der(2)t(2;7)(q37; q36),del(7)(q36).

Parental karyotypes revealed a normal constitution.

**Discussion:** Deletion of the 2q37 locus is one of the most commonly observed anomalies involving subtelomeric regions described in the literature. However, it is not frequently described in prenatal diagnosis, where association with CNS malformations and IUGR were described.

The results obtained relate to the ultrasound findings observed in this fetus.

In the present case, the use of array-CGH and karyotype analysis provided a better understanding of the chromosome imbalance, therefore stressing that in many circumstances it is very important to rely on them as complementary techniques.

Genotype-phenotype correlations are crucial for genetic counselling and the orientation of medical decisions.

**P54 –** AUTOSOMAL DOMINANT INTELLECTUAL DISABILITY 43 – NOT ALWAYS *DE NOVO*.

Maria Abreu^1^; Sónia Figueiroa^2^; Rita Cerqueira^3^; Jorge Pinto Basto^3^; Ana Maria Fortuna^4^; Cláudia Falcão Reis^5^

^1^Medical Genetics Department, Centro de Genética Médica Jacinto de Magalhaães (CGMJM), Centro Hospitalar Universitário do Porto (CHUP); ^2^Division of Pediatric Neurology, Department of Child and Adolescent, Centro Hospitalar Universitário do Porto; ^3^Laboratório de Diagnóstico Molecular e Genómica Clínica, CGC Genetics, Unilabs; ^4^Medical Genetics Department, Centro de Genética Médica Jacinto de Magalhães (CGMJM), Centro Hospitalar Universitário do Porto; Unit for Multidisciplinary Research in Biomedicine (UMIB), Instituto de Ciências Biomédicas Abel Salazar; ^5^Medical Genetics Department, CGMJM, CHP; Life and Health Sciences Research Institute (ICVS), School of Medicine, University of Minho, Braga, Portugal; ICVS/3B's - PT Government Associate Laboratory, Braga/Guimarães, Portugal

**Introduction:** Moderate to severe intellectual disability (previously known as mental retardation) with autosomal dominant inheritance (MRD) is very often caused by de novo variants. Pathogenic variants in MRD disorders with complete penetrance are frequently presumed to be de novo in the absence of segregation studies. In those instances, a theoretical recurrence risk estimated at 1% or lower is given to healthy parents.

MRD43 (# 616977) is an intellectual disability syndrome with neurological manifestations associated with loss-of- function *HIVEP*2 heterozygous variants.

**Clinical case:** A 33-mo boy referred to Genetics for moderate developmental delay, hypotonia, wide-based gait, dysmorphic features, strabismus, and hypermetropia. He was the only child to healthy non-consanguineous parents with unremarkable family history. Array-CGH and general biochemistry were inconclusive. Brain MRI showed mild encephalic white matter reduction and corpus callosum hypoplasia. WES-based NGS panel with 1502 developmental delay/ID genes disclosed a previously described heterozygous nonsense pathogenic *HIVEP2* variant [c.2827C>T p.(Arg943∗)], establishing an MRD43 diagnosis. Segregation studies showed the variant was present in the mother with somatic mosaicism in 15% of peripheral blood cells. After genetic counselling with a maximum recurrence risk of 50%, the couple opted for PGD and preliminary haplotype studies are ongoing.

**Discussion:** To our knowledge, this description of an inherited *HIVEP2* pathogenic variant is unique in the literature. The maternal mosaicism was well tolerated despite considerable expression in the blood sample and, likely, in the gonadal tissue. This clinical case highlights that a previously reported pathogenic variant in an MDR gene can in fact be inherited, dramatically increasing the recurrence risk and significantly impacting parents’ reproductive options. Parental testing should be mandatory for pathogenic variants in this context, especially when parents consider further family planning.

**P55 –** PRENATAL DIAGNOSIS OF CONGENITAL HEART DISEASE IN A FETUS WITH A 8p23.1 INTERSTITIAL DELETION

Laurentino R. Simão^1^; Bárbara S. Marques^1^; Sílvia S. Serafim^1^; Cristina M. Ferreira^1^; Ana R. Tarelho^1^; Ana C. Alves^1^; Filomena T. Brito^1^; Marisa D. Silva^1^; Neuza S. Silva^1^; Sónia I. Pedro^1^; Inês S. Carvalho^2^; Ana T. Martins^3^; Álvaro E. Cohen^3^; Hildeberto O. Correia^1^

^1^Instituto Nacional de Saúde Doutor Ricardo Jorge, I.P., Lisboa; ^2^Serviço de Genética Médica, Hospital D. Estefânia, C. H. Universitário Lisboa Central, EPE, Lisboa; ^3^Centro de Diagnóstico Pré-Natal, Maternidade Dr. Alfredo da Costa, C. H. Universitário Lisboa Central, EPE, Lisboa

**Introduction:** Congenital heart disease (CHD) is the most common form of birth defects. The incidence of CHD is about 0.8% to 1% in live-born, full-term births, and it is ten times higher in preterm infants (8.3%). The atrioventricular septum defect (AVDS) is the most common CHD detectable in utero. AVSD is known to occur in either a nonsyndromic (isolated) form or, more commonly, as part of a malformation syndrome.

**Methodology:** A 30-year-old woman at 12 weeks of gestation was referred for prenatal diagnosis due to fetal AVSD.

Chromosomal microarray analysis (CMA) was carried out after a normal molecular rapid aneuploidy test result.

**Results:** CMA identified, in a male fetus, a 3.11 Mb interstitial deletion at 8p23.1 - arr[GRCh37] 8p23.1(8824857_11935465)x1.

This region encompasses 17 OMIM genes including *GATA4*. This protein is thought to regulate genes involved in embryogenesis and in myocardial differentiation and function.

Parental testing was requested and CMA was performed revealing that the deletion is *de novo*.

**Discussion:** Deletions and mutations of the *GATA4* gene are associated with cardiac septal defects.

This deletion has a pathogenic clinical significance.

The AVSD found in the fetus can be explained by the observed genomic change.

Interstitial deletions of 8p23.1 are associated with a variable spectrum of anomalies that include congenital heart malformations. The prevalence is unknown but 8p23.1 deletions are rare. Most 8p deletions occurs *de novo*.

The accuracy of cardiac defects in obstetric ultrasound and the identification of the genetic cause provide more knowledge for the genetic counseling. The parents opted to terminate the pregnancy.

**P56 –** THE GENETIC ANALYSIS OF PORTUGUESE PATIENTS WITH CEREBELLAR ATAXIA, NEUROPATHY, VESTIBULAR AREFLEXIA SYNDROME (CANVAS): A FREQUENT AND GENETICALLY COMPLEX CLINICAL ENTITY

Ana Lopes^1^; Maria João Malaquias^2^; Luís Braz^3^; Ana Aires^3^; Ana Luísa Sousa^4^; Ana Paula Sousa^5^; Catarina Cruto^6^; Cristina Alves^2^; Cristina Costa^7^; Cristina Rosado Coelho^8^; Eva Brandão^4^; Goreti Nadais^9^; Joana Guimarães^3^; Nuno Vila-Chã^2^; Pedro Abreu^3^; Raquel Barbosa^10^; Raquel Samões^2^; Ricardo Taipa^11^; Rui Araújo^3^; Teresa Pimentel^12^; Vítor Tedim Cruz^6^; André Caetano^10^; Margarida Calejo^6^; Paula Salgado^6^; Bravo Marques^13^; Joana Damásio^14^; Daniela Garcez^12^; Sara Morais^1^; João Parente Freixo^1^; Marina Magalhães^15^; Jorge Oliveira^1^

^1^Centro de Genética Preditiva e Preventiva (CGPP), Instituto de Biologia Celular e Molecular (IBMC), Instituto de Investigação e Inovação em Saúde (i3S), Universidade do Porto, Porto; ^2^Serviço de Neurologia, Centro Hospitalar Universitário do Porto, Porto; ^3^Serviço de Neurologia, Centro Hospitalar Universitário de São João, Porto; Departamento Neurociências Clínicas e Saúde Mental da Faculdade de Medicina da Universidade do Porto; ^4^Centro Hospitalar de Entre Douro e Vouga, Santa Maria da Feira, Aveiro; ^5^Serviço de Neurofisiologia, Centro Hospitalar Universitário do Porto, Porto; ^6^Serviço de Neurologia, Unidade Local de Saúde de Matosinhos, Porto; ^7^Serviço de Neurologia, Hospital Prof. Doutor Fernando da Fonseca, Lisboa; ^8^Serviço de Neurologia, Centro Hospitalar de Setúbal, Setúbal; ^9^Serviço de Neurologia, Centro Hospitalar Universitário de São João, Porto; ^10^Serviço de Neurologia, Centro Hospitalar de Lisboa Ocidental, Lisboa; ^11^Unidade de Neuropatologia, Centro Hospitalar Universitário do Porto, Porto; ^12^Serviço de Neurologia, Instituto Português de Oncologia de Lisboa Francisco Gentil, Lisboa; ^13^Fundação Champalimaud; ^14^Centro de Genética Preditiva e Preventiva (CGPP), Instituto de Biologia Celular e Molecular (IBMC), Instituto de Investigação e Inovação em Saúde (i3S), Universidade do Porto, Porto; Serviço de Neurologia, Centro Hospitalar Universitário do Porto, Porto; ^15^Serviço de Neurologia, Centro Hospitalar Universitário do Porto, Porto; Serviço de Neurologia, Hospital Padre Américo, Centro Hospitalar do Tâmega e Sousa, Penafiel, Porto

**Introduction:** In 2019, Cortese *et al.* established that diallelic expansion of an intronic (AAGGG)n in *RFC1* (400_2,000 repeats), is the genetic cause of the cerebellar ataxia, neuropathy, vestibular areflexia syndrome (CANVAS). That tract is very heterogeneous in the general population. Four main allelic variants were described: the (AAAAG)11 reference allele (freq. = 0.755); expanded AAAAG or AAAGG repeats (freq. = 0.130 and 0.079); and the pathogenic (AAGGG)n expansion (freq. = 0.007)- this relatively high frequency suggests that CANVAS may represent a considerable fraction of late- onset ataxias.

**Methodology:** Genetic analysis was based on the approach described by Cortese *et al.* (2019): (1). a fluorescently labelled PCR was used to amplify the repeat's region; (2).it was multiplexed with a set of control primers to check DNA amplification. Presence of *RFC1* PCR products precludes a diagnosis of CANVAS, although the patient may still be a carrier. (3) Three specific repeat-primed PCRs (RP-PCRs) were performed, each targeting one of the known pentanucleotides - presence of the continuous stutter peak profile in the AAGGG-specific RP-PCR, and absence of similar results in the other two PCRs, is compatible with the diagnosis of CANVAS.

**Results:** A total of 43 CANVAS patients were ascertained. Most of them had already been tested for other ataxias, through targeted approaches and/or NGS ataxia gene panels. Until now, 24 patients (from 20 families) have been found with the diallelic (AAGGG)n expansion; 4 were carriers. Age of onset was 54.8 ± 10.3 years. The most frequent symptoms at onset were unsteady gait (n = 19) and sensory complaint (n = 9). A sequencing technique is still being implemented to confirm the presence of the (AAGGG)n expansion.

**Discussion:** This is the first report of genetically characterized CANVAS in Portugal. As further cases are studied, this cohort could expand significantly. Sizing the expansion, although technically challenging, is relevant for genotype-phenotype correlations and clinical management. Testing the (AAGGG)n expansion in undiagnosed patients with late-onset ataxia (especially those with the typical clinical triad) is highly recommended.

**P57 –** BEHAVIOURAL DISORDERS IN TATTON- BROWN-RAHMAN SYNDROME: A CASE REPORT

João Rodrigues Alves^1^; Juliette Dupont^1^; Cristina Rebordão^2^; Ana Berta Sousa^1^

^1^Serviço de Genética Médica, Hospital de Santa Maria, Centro Hospitalar Universitário Lisboa Norte, Centro Académico de Medicina de Lisboa, Lisboa; ^2^Serviço de Psiquiatria e Saúde Mental da Infância e Adolescência, Hospital Pulido Valente, Centro Hospitalar Universitário Lisboa Norte, Centro Académico de Medicina de Lisboa, Lisboa

**Introduction:** Tatton-Brown-Rahman Syndrome (TBRS) is caused by mutations in *DNMT3A,* and characterized by intellectual disability (ID), distinctive facial appearance and overgrowth. Studies regarding its cognitive and behavioural profiles have revealed a better performance in verbal tasks and a high prevalence of autism spectrum disorder (ASD).

**Methodology:** Clinical data were collected from the patient's medical records and clinical observations encompassing a period of eight years.

**Results:** We report a 19-year-old male with unremarkable family history, pregnancy and delivery. Somatometry went above the 97th centile postnatally. Skeletal findings comprised macrocephaly, disproportionate tall stature, thoracolumbar kyphosis, vertebral fusion, cubitus valgus, arachnodactyly and hyperlaxity. Progressive coarsening of facial features, heavy horizontal eyebrows, deep-set eyes and narrow palpebral fissures were noted. He was diagnosed with moderate ID, ASD and a disruptive, impulse-control and conduct disorder. Mood stabilizers were effective in decreasing disruptive episodes, which were characterized by irritability, temper tantrums, aggressive and defiant behaviour. Psychotic symptoms, including auditory hallucinations and episodes of euphoria were noted, although not meeting criteria for psychosis or a mood disorder. Genetic testing with whole exome sequencing identified a previously unreported *de novo* variant in *DNMT3A*:c.1657_1659del, p.(Asn553del).

**Discussion:***DNMT3A* encodes a DNA methyltransferase, which is essential for establishing methylation during embryogenesis. The variant identified in this patient is located in a critical ATRX-Dnmt3-Dnmt3L functional domain. The patient's skeletal and dysmorphic features, as well as ID and ASD are typical of TBRS. However, psychotic symptoms and disruptive behaviour have been reported infrequently and their course and management overlooked. Although intensive clinical follow-up is not usually needed in patients with TBRS, we highlight that disruptive behaviour and psychiatric complications may lead to a heavy disease burden to patients and their families if not appropriately identified and treated.

**P58 –** A CLINICAL CASE OF MITOCHONDRIAL DISORDER: BUT NOT ONLY

Sofia Nunes^1^; Manuela Grazina^2^; Fernando Pita^3^; Jorge Sequeiros^4^; Teresinha Evangelista^5^; Cristina Costa^6^; Mafalda Melo^1^; Rui Gonçalves^1^

^1^Genetic Service, Pediatric Department, Centro Hospitalar Universitário de Lisboa Central, EPE; ^2^Center for Neuroscience and Cell Biology, Centro Hospitalar Universitário de Coimbra, EPE; ^3^Neurology Service, Hospital Garcia de Orta; ^4^Centro de Genética Preditiva e Preventiva, Instituto de Biologia Molecular e Celular, CGPP-IBMC; ^5^Neuropathology Laboratory, Neurology Service, Centro Hospitalar Lisboa Norte, EPE; ^6^Neurology Service, Hospital Professor Doutor Fernando Fonseca, EPE

**Introduction:** Mitochondrial disorders are a heterogeneous group of disorders caused by primary dysfunction of the respiratory chain. Mitochondrial myopathies frequently present with multi-system dysfunction, an extensive variability of phenotypes and genetic etiologies, as well as a generally poor genotype–phenotype correlation.

**Case Report:** Here we report a case of a 30 year-old female referred to our clinic by ataxia with symptom onset at 22 years of age. Family history was inconclusive for hereditary pathologies.

At clinical observation, she had appendicular and axial ataxia, hyperreflexia, dysarthria, decreased muscle strength of the four limbs, vertical nystagmus with occasional diplopia. A progressive worsening of the symptoms was noted.

Regarding complementary diagnostic tests, we highlight: normal echocardiogram and electrocardiogram; normal analytical assessment of liver, renal and thyroid function; normal level of vitamin E; normal metabolic investigation; brain MRI revealed cerebellar atrophy with symmetrical involvement of the vermis and hemispheres; muscle biopsy revealed aspects that may be compatible with myofibrillar myopathy.

As a differential diagnosis, we considered the groups of mitochondrial myopathies and spinocerebellar ataxias.

Genetic study of mitochondrial DNA in muscle tissue cells revealed a 4977 bp deletion that includes the genes *ATPase8*, *ATPase6*, *COXIII*, *tRNAGly*, *ND3*, *tRNAA rg*, *ND4L*, *ND4*, *tRNAHis*, *tRNASer2*, *tRNALeu2* and *ND5*, compatible with mitochondrial myopathy. Additionally, Next Generation Sequencing (NGS) panel identified two pathogenic variants in *ANO10* gene, in compound heterozygosity, compatible with the diagnosis of autosomal recessive spinocerebellar ataxia type 10.

The patient started treatment with coenzyme q10, levocarnitine, creatine and a multivitamin complex.

**Conclusion:** We report a case of overlapping mitochondrial myopathy and autosomal recessive spinocerebellar ataxia type 10.

We emphasize the importance of a multidisciplinary approach, especially in the diagnosis of rare diseases and highlight the importance of a thorough diagnostic investigation in order to be able to identify situations of overlapping diagnoses.

**P59 –** A NOVEL CASE OF AUTOSOMAL RECESSIVE CUTIS LAXA TYPE 1C: CASE REPORT AND LITERATURE REVIEW

Mafalda S. Melo^1^; Susana L. Ferreira^1^; Sofia Nunes^1^; Maria Sacras^2^; Fátima Abreu^3^; Tiago Rito^4^; Salomé Almeida^5^; Teresa Kay^1^; Diana Antunes^1^

^1^Serviço de Genética Médica, Hospital Dona Estefânia, Centro Hospitalar e Universitário de Lisboa Central, Lisboa, Portugal; ^2^Serviço de Cirurgia Pediátrica, Hospital Dona Estefânia, Centro Hospitalar e Universitário de Lisboa Central, Lisboa, Portugal; ^3^Unidade de Pneumologia Pediátrica, Hospital Dona Estefânia, Centro Hospitalar e Universitário de Lisboa Central, Lisboa, Portugal; ^4^Serviço de Cardiologia Pediátrica, Hospital Santa Marta, Centro Hospitalar e Universitário de Lisboa Central, Lisboa, Portugal; ^5^Centro de Investigação, Centro Hospitalar e Universitário de Lisboa Central, Lisboa, Portugal

**Background:** Autosomal recessive cutis laxa type 1C (ARCL1C, MIM #613177) is a rare connective tissue disorder caused by *LTBP4* mutations. ARCL1C presents with cutis laxa, early childhood-onset pulmonary emphysema, peripheral pulmonary artery stenosis, and other evidence of a generalized connective disorder such as hernias. To date, only 19 patients have been reported.

**Case report:** A 4-month-old boy was referred to our clinic for multiple congenital anomalies evaluation. He was the child of a consanguineous and healthy Cape Verdean couple. Clinical history included neonatal hypotonia, congenital heart disease characterized by moderate peripheral pulmonary artery stenosis, ostium secundum atrial septal defect and mild aortic valve insufficiency, diaphragmatic hernia, and inguinal hernia. At our observation, he had loose skin with sagging cheeks, sloping forehead, periorbital fullness, depressed nasal bridge with anteverted nares, everted lower lip and micrognathia. A clinical diagnosis of cutis laxa was given due to the presence of the dermatological hallmark. WES-based virtual panel revealed a homozygous splice site variant in *LTBP4* gene [NM_003573.2:c.780+2T>G p.?], classified as pathogenic, establishing the diagnosis of ARCL1C.

**Discussion:** This specific variant was only reported in one case. The case was a Cape Verdean girl who had a similar presentation and died by the age of two due to pulmonary emphysema. Up to the moment, our patient did not show signs of respiratory distress. However, multidisciplinary evaluations are planned including periodic assessment of pulmonary function and imaging of gastrointestinal and urinary tracts. Also, the patient developed neck abscesses, which we hypothesized it could be a new ARCL1C feature.

**Conclusion:** Data from patients are needed to better characterize the *LTBP4*-related phenotype and define clinical diagnostic criteria, genotype-phenotype correlations, and prognosis. By reporting cervical abscesses as a possible additional feature related to *LTBP4* mutations, this case further broadens the clinical spectrum of ARCL1C. Additionally, it enabled an appropriate surveillance and genetic counselling to the patient and family.

**P60 –** INTELLECTUAL DISABILITY, HYPOTONIA AND PALATE ABNORMALITIES – A CASE REPORT OF AU-KLINE SYNDROME

Raquel Gouveia Silva^1^; Oana Moldovan^1^; Ana Medeira^1^; Ana Berta Sousa^1^

^1^Serviço de Genética, Departamento de Pediatria, Hospital de Santa Maria, Centro Hospitalar Universitário Lisboa Norte, Centro Académico de Medicina de Lisboa, Lisboa, Portugal

**Introduction:***HNRNPK* gene encodes the heterogeneous nuclear ribonucleoprotein K, which plays a role in cellular regulation and bone homeostasis. *HNRNPK* haploinsufficiency causes Au-Kline syndrome (AKS) characterized by moderate to severe intellectual disability (ID), hypotonia, and typical craniofacial features. Congenital heart disease, hydronephrosis, palate abnormalities, and oligodontia are reported in most patients. Additional findings include visual anomalies, variable autonomic dysfunction, urogenital and skeletal problems. We are aware of at least 25 patients worldwide, of whom 12 reported in the literature (9 bearing point mutations and 3 with microdeletions in 9q21.32 encompassing the *HNRNPK* gene, all *de novo*).

**Methodology:** We describe an 18-year-old girl, the only child of a non-consanguineous couple. Family history was irrelevant. After birth, hypotonia, soft cleft palate with bifid uvula, dysplastic mitral valve and bicuspid aortic valve, and congenital hip dislocation were noticed. She evolved with moderate ID, significant motor delay, language impairment with barely noticeable sentences, stereotypies, short stature, and dysmorphisms. She had no autonomy in activities of daily living. Other relevant features included hypermetropia and ocular angiosclerosis, bilateral ureterohydronephrosis, leukopenia with recurrent respiratory infections, agenesis of the maxillary lateral incisors, joint laxity, scoliosis, anteversion of the femoral necks, external tibial torsion, and high pain tolerance.

**Results:** Solo whole exome sequencing was performed, after extensive investigation, revealing the presence of a heterozygous frameshift likely pathogenic variant in *HNRNPK* gene.

**Discussion:** Although the prevalence of AKS is yet to be determined, it should be considered in the differential diagnosis of overlapping conditions, namely Kabuki syndrome. There are no significant phenotypic differences between point mutations and microdeletions, supporting *HNRNPK* haploinsufficiency as the primary mechanism. Further case reporting and data sharing are essential to better delineate this syndrome and improve patient management.

**P61 –** GENETIC AND CLINICAL REPORT OF TEN ADDITIONAL PORTUGUESE PATIENTS WITH *PNKP*-RELATED ATAXIA (AOA4)

Susana Sousa^1^; João Parente-Freixo^1^; Isabel Alonso^1^; Paulo Silva^1^; Ana Moreira^2^; Leandro Valdemar^3^; Miguel Pereira^4^; Carolina Soares^5^; Maria José Rosas^5^; Miguel Leão^6^; Laura Azurara^7^; Sofia Rocha^8^; José Carlos Ferreira^7^; Joana Damásio^9^; Jorge Sequeiros^1^; Jorge Oliveira^1^

^1^CGPP – Centro de Genética Preditiva e Preventiva, IBMC – Instituto de Biologia Molecular e Celular, i3S – Instituto de Investigação e Inovação em Saúde, Universidade do Porto, Portugal; ^2^Serviço de Neurologia, Hospital de Dona Estefânia, Centro Hospitalar Universitário de Lisboa Central, EPE, Lisboa, Portugal; ^3^Serviço de Neurologia, Hospital do Divino Espírito Santo da Ilha Terceira, EPE, Ponta Delgada, Portugal; ^4^Serviço de Neurologia, Centro Hospitalar e Universitário de Coimbra, EPE, Coimbra, Portugal; ^5^Consulta de Doenças do Movimento, Centro Hospitalar Universitário de São João, EPE, Porto, Portugal; ^6^Serviço de Genética Médica, Centro Hospitalar Universitário de São João/Faculdade de Medicina da Universidade do Porto, Portugal; ^7^Serviço de Neurologia Pediátrica, Hospital de São Francisco Xavier, Centro Hospitalar de Lisboa Ocidental, EPE, Lisboa, Portugal; ^8^Serviço de Neurologia, Hospital de Braga, Portugal; ^9^CGPP – Centro de Genética Preditiva e Preventiva, IBMC – Instituto de Biologia Molecular e Celular, i3S – Instituto de Investigação e Inovação em Saúde, Universidade do Porto, Portugal; Centro Hospitalar Universitário do Porto, Hospital de Santo António,

**Introduction:** Ataxia with oculomotor apraxia (AOA), the second most frequent cause of recessive ataxia in Portugal, is characterized by childhood-onset cerebellar ataxia, sensorimotor axonal neuropathy and oculomotor apraxia. AOA type 4 (AOA4) displays a varying degree of cognitive impairment and dystonia. In 2015, our group demonstrated that diallelic variants in the polynucleotide kinase 3’- phosphatase (*PNKP*) gene cause AOA4.

**Objectives:** Report additional AOA4 patients, identified during routine diagnostics; and update the mutational spectrum of *PNKP* gene.

**Methods:** We reviewed the genetic and clinical data from a sequential series of patients found to have pathogenic variants in *PNKP* at our laboratory, from 2015 to 2019. Genetic analysis was performed by conventional (Sanger) sequencing of the *PNKP* gene or through a next-generation sequencing (NGS) gene panel (based on whole-exome sequencing) for recessive ataxias.

**Results:** A total of 10 Portuguese AOA patients (7 families) were found to be homozygotes (2 families) or compound heterozygotes (5 families), harbouring 4 known *PNKP*pathogenic variants: c.1221_1223del (p.Thr408del) and c.1123G > T (p.Gly375Trp) (each present in 4 families), c.1315_1330delinsGGGGACG (p.Arg439_Pro444delinsGlyAspAla) and c.1029+2T > C (r.spl) (each present in 1 family); and 2 novel variants: c.1510del(p.Arg504Glyfs∗) and c.1282_1283insACAAACCCAGAC (p.Ala428delinsAspLysProArgPro) – the pathogenicity of this insertion is being confirmed. Interestingly, all the 21 Portuguese patients identified so far have, in at least one of the disease's alleles, the variant p.Thr408del or p.Gly375Trp, providing a common mutational link between the 11 different genotypes in this cohort.

**Discussion:** This study expands the number of AOA4 patients genetically characterized by our group, from the 11 reported in 2015 to a total of 21. Furthermore, it confirms AOA4 as being amongst the most frequent subtypes of recessive ataxia in Portugal. Considering the limited number of additional patients from other populations reported in literature (n = 8), this cohort is paramount to further characterize AOA4 as a clinical entity.

**P62 –** NOVEL GENOTYPIC AND PHENOTYPIC FEATURES OF *CCDC22*-RELATED RITSCHER- SCHINZEL SYNDROME

Nuno Maia^1^; Ana Maria Fortuna^2^; Joana Damásio^3^; Rosário Santos^1^; Manuel Melo-Pires^4^; Arjan PM de Brouwer^5^; Paula Jorge^1^

^1^Unidade de Genética Molecular, Centro de Genética Médica Doutor Jacinto Magalhães (CGM), Centro Hospitalar Universitário do Porto (CHUP); Unidade Multidisciplinar de Investigação Biomédica (UMIB), Instituto de Ciências Biomédicas Abel Salazar (ICBAS), UP; ^2^Unidade de Genética Médica, Centro de Genética Médica Doutor Jacinto Magalhães (CGM), Centro Hospitalar Universitário do Porto (CHUP); Unidade Multidisciplinar de Investigação Biomédica (UMIB), Instituto de Ciências Biomédicas Abel Salazar (ICBAS), UP; ^3^Serviço de Neurologia, Centro Hospitalar Universitário do Porto (CHUP); Unidade Multidisciplinar de Investigação Biomédica (UMIB), Instituto de Ciências Biomédicas Abel Salazar (ICBAS), UP; ^4^Serviço de Neuropatologia, Centro Hospitalar e Universitário do Porto (CHUP); ^5^Department of Human Genetics, Donders Institute for Brain, Cognition and Behaviour, Radboud University Nijmegen, Nijmegen, The Netherlands.

**Background:***CCDC22* is implicated in the X-linked Ritscher-Schinzel syndrome 2 (RTSC2), an intellectual disability (ID) syndrome showing craniofacial, heart and brain abnormalities. RTSC2 has been described in eight patients (two unrelated families), showing missense *CCDC22* variants affecting N- and C-terminal amino acid residues. Herein, we describe the clinical and cascade genetic investigations of two affected brothers with a novel *CCDC22* variant.

**Methods:** After a negative first-tier genetic investigation, exome sequencing (ES) and CNV analysis were performed in the youngest sibling. Sanger sequencing and MLPA were used to confirm or exclude putative pathogenic variants.

**Results:** The two brothers, born to first-degree consanguineous parents, and with three maternal uncles showing mild ID, present facial dysmorphic features, brachydactyly, dysarthria, brisk deep tendon reflexes, limb rigidity and broad base gait. The oldest presented a delay in motor acquisitions and both have ID, with poor school achievements. Brain MRI revealed T2∗ hypointensities of the pallidum, and a small cystic formation of the vermis in one sibling. CNV analysis revealed a duplication in 17q21, excluded by MLPA. Sanger sequencing confirmed the presence of the *CCDC22* missense variant c.700C > T, p.(R234C) in both affected brothers, in the mother in heterozygosity, and absent in the healthy sister. Other familial studies are ongoing.

**Discussion:** The missense variant c.700C > T was considered likely pathogenic due to its rarity (2.42e-5 in gnomAD) and the large physicochemical difference between arginine and cysteine. Despite the scarcity of clinical information, previously reported RTSC2 patients were reviewed in terms of phenotype and genotype. Interestingly, no cardiac impairment was noticed in our patients, which might be explained by the fact that a distinct protein region is affected. Overall, this study further widens the genotypic and phenotypic spectrum of the very rare condition RTSC2. Nevertheless, the deleterious effect of this variant requires further investigation, to ultimately contribute towards deciphering the phenotypic outcomes of specific *CCDC22* variants.

**P63 –** CHROMOSOME 10q23 DELETION SYNDROME – CASE REPORT AND LITERATURE REVIEW

Daniela Oliveira^1^; Isabel Dinis^2^; Cláudia Piedade^3^; Ricardo Ferreira^4^; Maria Francelina Lopes^5^; Jorge Saraiva^6^; Sofia Maia^7^

^1^Medical Genetics Unit, Hospital Pediátrico, Centro Hospitalar e Universitário de Coimbra, Coimbra, Portugal; ^2^Pediatric Endocrinology Unit, Hospital Pediátrico, Centro Hospitalar e Universitário de Coimbra, Coimbra, Portugal; ^3^Pediatric Surgery Unit, Hospital Pediátrico, Centro Hospitalar e Universitário de Coimbra, Coimbra, Portugal; ^4^Pediatric Gastroentherology Unit, Hospital Pediátrico, Centro Hospitalar e Universitário de Coimbra, Coimbra, Portugal; ^5^Pediatric Surgery Unit, Hospital Pediátrico, Centro Hospitalar e Universitário de Coimbra, Coimbra, Portugal; University Clinic of Surgery, Faculty of Medicine, University of Coimbra, Portugal; Clinical Academic Center of Coimbra, Coimbra, Portugal; ^6^Medical Genetics Unit, Hospital Pediátrico, Centro Hospitalar e Universitário de Coimbra, Coimbra, Portugal; Clinical Academic Center of Coimbra, Coimbra, Portugal; University Clinic of Pediatrics, Faculty of Medicine, University of Coimbra, Portugal; ^7^Medical Genetics Unit, Hospital Pediátrico, Centro Hospitalar e Universitário de Coimbra, Coimbra, Portugal; University Clinic of Genetics, Faculty of Medicine, University of Coimbra, Portugal

**Context:** Chromosome 10q23 Deletion Syndrome is a rare entity defined by a germline chromosomal deletion that encompasses *PTEN* and *BMPR1A* genes associated with *PTEN* Hamartoma Tumor Syndrome (PHTS) and Juvenile Polyposis Syndrome (JPS), respectively. Patients usually have severe juvenile polyposis with gastrointestinal bleeding, refractory anemia and protein-losing enteropathy. Other associated features include macrocephaly, thyroid disease, congenital heart defects, developmental delay and an increased malignancy risk. There are 26 patients described in the literature with a limited follow-up period.

**Case Report:** We describe a 16-year-old male who was diagnosed at the age of 1 year with patent ductus arteriosus and interatrial communication, post-natal short stature, macrocephaly, mild developmental delay and minor dysmorphisms. He later developed a refractory anemia due to recurrent hematochezia, chronic diarrhea with hypoalbuminemia, rectal prolapse and abdominal distension. Array-CGH analysis detected a *de novo* 3.34Mb deletion at 10q23.1q23.31 and allowed us to establish the diagnosis at the age of 6 years. A follow-up program was implemented with gastrointestinal surveillance and thyroid, abdominal and pelvic ultrasound. At the age of 7 years he was submitted to a subtotal colectomy and multiple endoscopic polypectomy, which lead to significant clinical improvement. The histological study revealed juvenile polyposis. At the age of 15 years a total thyroidectomy was performed and histological analysis unveiled a papillary microcarcinoma.

**Discussion:** Reported patients with Chromosome 10q23 Deletion Syndrome have variable phenotypes with severe gastrointestinal symptoms, prominent extraintestinal features and an increased risk of colorectal, thyroid and ovary tumors. The report of long term follow-up of patients such as ours is crucial for the development and publication of specific surveillance and treatment guidelines. In the absence of these, we proposed for this patient a follow-up plan based on the adaptation of current recommendations for both PHTS and JPS.

**P64** – A NOVEL MISSENSE VARIANT CAUSING *GATAD2B*-ASSOCIATED NEURODEVELOPMENTAL DISORDER (GAND)

Ana Grangeia^1^; Rita Quental^1^; Ana L. Leite^2^; Ana C. Maia^2^; Miguel Leão^1^

^1^Serviço de Genética, Centro Hospitalar Universitário de São João; ^2^Serviço de Pediatria, Centro Hospitalar de Vila Nova de Gaia/Espinho

**Introduction:** Pathogenic variants in the *GATAD2B* gene have been associated with a neurodevelopmental disorder (GAND, MIM 615074) characterized by childhood hypotonia, severe intellectual disability (ID), macrocephaly and dysmorphic features. Since its first description in 2013, only seven subjects were identified with *GATAD2B* missense variants that localized within the two conserved region domains (CR1 or CR2) of the GATAD2B protein.

Here, we report a 12-year-old boy with macrocephaly and ID with a novel *GATAD2B* missense variant.

**Results:** Our male patient was born at term after an uneventful pregnancy. During the first two years of life, height and weight followed the 50th with posterior growth deceleration by age of 5. The height dropped to the 5th percentile and he had a bone age delay of 4 years. All developmental milestones were abnormal from early infancy. His IQ was 56. Hearing loss, hypermetropia, constipation, obsessions and severe developmental co- ordination disorder were also noted. Brain MRIs was normal. At age 10 he presented ID, normal weight, height below the 5th percentile, head circumference in 98th percentile, scoliosis and *pectus carinatum*. Fragil X, aCGH and *FOXP1* gene sequencing analyses were normal. *Trio-based WES r*evealed a *de novo* missense variant in *GATAD2B* (NM_020699: c.1136T > C; p.(Phe379Ser)). This variant is not reported in the population GnomAD or disease ClinVar databases and bioinformatic analysis predicted the variant as disease- causing.

**Discussion:** A recent study proposed that missense variants within GATAD2B CR domains disrupted assembly of nucleosome remodelling and deacetylase (NuRD) complex proteins, explaining the clinical overlap with other disorders associated with the NuRD complex (*CHD3* and *CHD4* genes) like hypotonia, intellectual disability, childhood apraxia of speech and macrocephaly (1). Here we reported a patient with ID and macrocephaly with a novel missense variant located within the CR2 of the GATAD2B protein. His clinical features are in accordance with reported GAND's clinical phenotype.

**References:** 1. Genetics in Medicine (2020) https://doi.org/10.1038/s41436-019-0747-z.

## Correction

In abstract P62, the affiliations for Ana Maria Fortuna and Joana Damasio were incorrect. Ana Maria Frotuna's has been corrected from 3 to 2. Joana Damasio's has been corrected from 2 to 3.

